# Hybridization Probes Featuring a Pyrenylpyridine *C*‐Nucleoside or Its Palladacycle as a Fluorescent Sensor Moiety

**DOI:** 10.1002/cbic.202500474

**Published:** 2025-07-21

**Authors:** Dattatraya Uttam Ukale, Tuomas Lönnberg

**Affiliations:** ^1^ Department of Chemistry University of Turku Henrikinkatu 2 20100 Turku Finland

**Keywords:** base pairing, fluorescence, hybridization, oligonucleotides, palladacycle

## Abstract

A *C*‐nucleoside analog having pyren‐1‐ylpyridine as the base moiety has been synthesized and incorporated in the middle of a short oligodeoxynucleotide. A portion of this oligonucleotide is cyclopalladated at the modified residue, and the potential of both the metal‐free and the palladacyclic oligonucleotide as hybridization probes for single‐nucleotide polymorphism genotyping is assessed by melting studies on relevant duplexes using various techniques. Conventional ultraviolet (UV) melting profiles at 260 nm reveal considerable destabilization of the palladacyclic duplexes relative to their metal‐free counterparts. Circular dichroism melting temperatures are higher than their UV counterparts, especially with the palladacyclic duplexes. Cyclopalladation markedly reduces the fluorescence emission of the pyrenylpyridine moiety, but both the metal‐free and the palladacyclic oligonucleotide exhibit a qualitatively similar pattern of increased fluorescence on hybridization with a complementary sequence, consistent with the pyrene ring being “pushed out” of the base stack. Emission at low temperature is dependent on the nucleobase paired with the pyrenylpyridine base surrogate with both of the modified oligonucleotides. This discrimination is stronger with the palladacyclic oligonucleotide, possibly owing to Pd(II)‐mediated base pairing.

## Graphical abstract

When placed in the middle of a double helix as a nucleobase surrogate, pyren‐1‐ylpyridine and its palladacyclic derivative show fluorescence emission sensitive to the identity of the canonical nucleobase opposite to it. Emission of the palladacycle is weaker but the base discrimination stronger, possibly because of Pd(II)‐mediated base pairing. More emissive metallacycles can find use in genotyping single‐nucleotide polymorphisms.

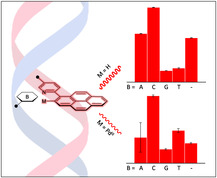

## 1 Introduction

Organometallic modification can furnish oligonucleotides with interesting properties hardly attainable through other means.^[^ ^1^, ^2^, ^3^, ^4^ ^]^ Most of the research in this relatively new field has focused on either redox labels^[^ ^5^, ^6^, ^7^, ^8^, ^9^ ^]^ or metal‐mediated base pairing of organometallic nucleobase analogs.^[^ ^10^, ^11^ ^]^ Outside the realm of nucleic acid chemistry, metallacyclic complexes of the platinum group metals have been studied for their luminescent properties,^[^ ^12^, ^13^, ^14^ ^]^ and recently such structures have also been incorporated into oligonucleotides.^[^ ^15^ ^]^

We have been interested in metal‐mediated base pairing as a tool to discriminate between the canonical (as well as some noncanonical) nucleobases,^[^ ^16^, ^17^, ^18^, ^19^, ^20^, ^21^, ^22^, ^23^, ^24^, ^25^ ^]^ ideally allowing identification of single‐nucleotide polymorphisms (SNPs) using only a single oligonucleotide hybridization probe. Artificial arylmercury nucleobase analogs have been particularly promising in this regard, but so far differentiation between the base‐pairing partners has still been based on overall stability of the double helix formed between the probe and the target, quantified as either melting temperature or fluorescence emission from a molecular beacon. As duplex stability is the complex product of many more interactions than just base pairing at the polymorphic site, another signal to complement this data, preferably arising from the base pairing interaction itself, would be desirable. Fluorescence emission from a modified nucleobase is already widely used to distinguish between secondary structures^[^ ^26^, ^27^, ^28^, ^29^, ^30^, ^31^, ^32^, ^33^, ^34^ ^]^ and applications to SNP genotyping through hydrogen‐bonded base pairing^[^ ^35^, ^36^, ^37^, ^38^, ^39^, ^40^, ^41^ ^]^ base have also been reported. Metal‐mediated base pairing of fluorescent nucleobase analogs has also been reported^[^ ^42^, ^43^, ^44^, ^45^ ^]^ but, to the best of our knowledge, not yet studied in the context of SNP genotyping.

The present study describes our first attempts to combine fluorescent properties and metal‐mediated base pairing in a single organometallic nucleobase analog and assessment of the potential of a corresponding oligonucleotide as a hybridization probe for SNP genotyping. We reasoned that the fusion of pyrene, a known fluorophore, and phenylpyridine palladacycle, studied previously in the context of Pd(II)‐mediated base pairing,^[^ ^46^, ^47^ ^]^ would be an interesting structure to start this journey.

## 2 Results and Discussion

### 2.1 Building Block Synthesis

**Scheme 1:**
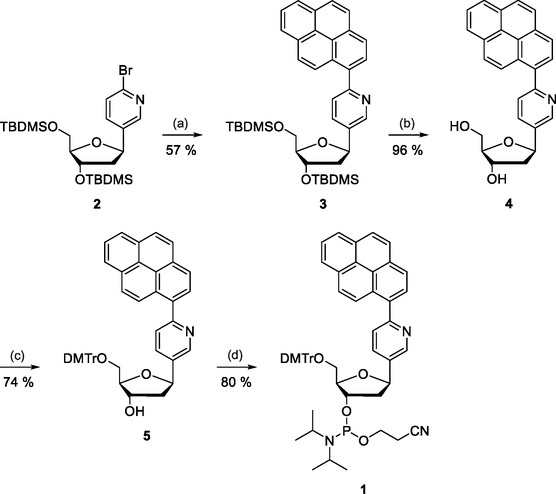
Synthesis of the pyrenylpyridine *C*‐nucleoside building block **1**. Reagents and conditions: a) pyrene‐1‐boronic acid, Pd(PPh_3_)_4_, K_2_CO_3_, H_2_O, MeOH, Ar atmosphere, reflux, 15 h; b) Et_3_N•3HF, THF, 25 °C, 16 h; c) DMTrCl, pyridine, 25 °C, 48 h; and d) 2‐cyanoehtyl‐*N*,*N*‐diisopropylchlorophosphoramidite, Et_3_N, CH_2_Cl_2_, N_2_ atmosphere, 25 °C, 150 min.

Synthesis of the pyrenylpyridine *C*‐nucleoside phosphoramidite building block **1** is outlined in **Scheme** **1**. First, a pyrenyl group was introduced to the protected bromopyridine *C*‐nucleoside **2** by Suzuki coupling. The silyl protections of the resulting intermediate **3** were then removed by conventional fluoride treatment. Reprotection of the exposed 5′‐hydroxy group of intermediate **4** as a 4,4′‐dimethoxytrityl ether afforded intermediate **5** and phosphitylation of the 3′‐hydroxy group the desired phosphoramidite building block **1**.

### 2.2 Oligonucleotide Synthesis

**Scheme 2:**
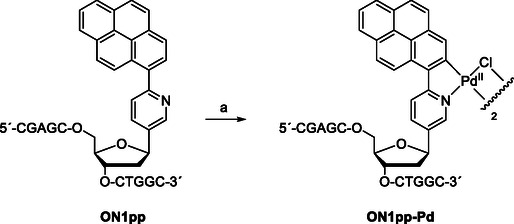
Cyclopalladation of oligonucleotide **ON1pp**. Reagents and conditions: a) Li_2_PdCl_4_, NaOAc, H_2_O, 55 °C, 16 h.

**Table** **1** summarizes the sequences of the oligonucleotides used in the present study. The modified oligonucleotide **ON1pp** was synthesized on an automated DNA/RNA synthesizer. Standard phosphoramidite strategy was followed otherwise, but coupling time of the pyrenylpyridine *C*‐nucleoside building block **1** was extended to 300 s, resulting in a near‐quantitative yield. After chain assembly, the oligonucleotide was released from the solid support and deprotected by conventional ammonolysis. Cyclopalladation of **ON1pp** was accomplished by overnight incubation with an excess of lithium tetrachloropalladate in aqueous sodium acetate (**Scheme** **2**). **ON1pp** and its cyclopalladated derivative **ON1pp‐Pd** were both purified by reversed‐phase high performance liquid chromatography (RP‐HPLC), characterized by electrospray ionization time‐of‐flight mass spectrometry (ESI‐TOF‐MS) and quantified by ultraviolet (UV) spectrophotometry. The cyclopalladation reaction is known to afford a chlorido‐bridged dimer as the immediate product but—in line with previous reports—only a single **ON1pp‐Pd** oligonucleotide with no exchangeable ligands was detected by mass spectrometry (MS). Evidently, the dimer dissociates during chromatographic purification, as reported previously for other palladacyclic oligonucleotides.^[^ ^46^, ^47^, ^48^, ^49^, ^50^ ^]^ The identity of the exchangeable ligands displacing the chlorido bridges remains elusive, but likely candidates include triethylamine and acetate ion from the HPLC elution buffer.

**Table 1:** Oligonucleotides used in this study.

Oligonucleotide	Sequence
**ON1pp**	5′‐CGAGCPpCTGGC‐3′a)
**ON1pp‐Pd**	5′‐CGAGCPp^Pd^CTGGC‐3′
**ON2a**	5′‐GCCAGAGCTCG‐3′
**ON2c**	5′‐GCCAGCGCTCG‐3′
**ON2g**	5′‐GCCAGGGCTCG‐3′
**ON2t**	5′‐GCCAGTGCTCG‐3′
**ON2s**	5′‐GCCAGSGCTCG‐3′

a) “Pp” and “PpPd” refer to the pyren‐1‐ylpyridine *C*‐nucleoside residue and its palladacyclic derivative and “S” to an abasic site (2‐(hydroxymethyl)tetrahydrofuran‐3‐ol spacer). The variable residues paired with each other on formation of a duplex are underlined.

### 2.3 UV Melting Profiles

Besides the usual absorption band at 260 nm, the UV spectra of both of the modified oligonucleotides (Figure S14 in the Supporting Information) featured another absorption band around 355 nm and that of **ON1pp‐Pd** also a weak one around 425 nm. The latter two stem solely from the pyrenylpyridine moiety and thus allow investigation of thermal transitions in the vicinity of this modification, in addition to the overall duplex melting. To this end, oligonucleotides **ON1pp** and **ON1pp‐Pd** were first allowed to hybridize with the complementary oligonucleotides **ON2a**, **ON2c**, **ON2g**, **ON2t**, and **ON2s**, pairing the pyrenylpyridine base with each of the canonical nucleobases or an abasic spacer. Melting profiles of the duplexes were then acquired at pH = 7.4 (20 mM cacodylate buffer) and *I* = 0.10 M (adjusted with NaClO_4_) by recording the absorbance at 260 and 355 nm and, in the case of **ON1pp‐Pd**, also at 425 nm.

**Figure 1:**
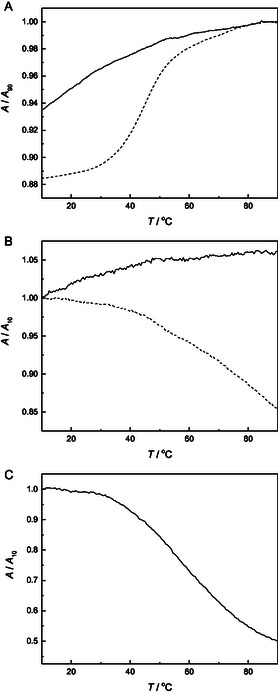
UV melting profiles of duplexes **ON1pp**•**ON2c** (dashed line) and **ON1pp‐Pd**•**ON2c** (solid line), recorded at A) 260, B) 355, and C) 425 nm; [oligonucleotides] = 1.0 μM; pH = 7.4 (20 mM cacodylate buffer); *I* = 0.10 M (adjusted with NaClO_4_).

At 260 nm, melting profiles of the duplexes formed by the metal‐free oligonucleotide **ON1pp** were sigmoidal and monophasic (the one for **ON1pp**●**ON2c** is presented in **Figure** **1**A as an illustrative example and the others in Figure S15—S24 in the Supporting Information). The melting temperatures ranged from 38 to 43 °C, typical for 11‐mer DNA heteroduplexes with an 80% GC content and a single intrachain mismatch. Notably, these values were only slightly higher than those previously reported for respective duplexes containing a phenylpyridine *C*‐nucleoside as the modified residue.^[^ ^46^ ^]^ In other words, expansion of the phenyl ring to a pyrene ring did not markedly stabilize the duplex, suggesting that it is not intercalated within the base stack. Hybridization affinity of the cyclopalladated oligonucleotide **ON1pp‐Pd** was much lower, with melting temperatures of the corresponding duplexes falling between 9 °C and 33 °C and none of the renaturation curves leveling off even at 10 °C. We have previously observed similar destabilization on cyclopalladation of related phenylpyridine‐modified oligonucleotides^[^ ^46^, ^47^ ^]^ and attributed it to incompatible geometries of the putative Pd(II)‐mediated base pair and the double helix. However, in the present case the effect was much more pronounced.

At 355 nm, the metal‐free and palladated duplexes exhibited strikingly different melting profiles (Figure 1B), the former with major hypochromicity and the latter with minor hyperchromicity on increasing temperature. The only exception from this pattern was **ON1pp‐Pd**●**ON2s**, placing the palladacyclic moiety opposite to an abasic site. The melting profile of this duplex resembled those of the metal‐free ones, tempting one to speculate that the drastically different results on the other palladated duplexes are due to Pd(II)‐mediated base pairing. Unfortunately, none of the melting profiles (Figure S25—S34 in the Supporting Information) showed sufficiently clear sigmoidicity to allow reliable determination of a melting temperature.

UV melting experiments at 425 nm were only carried out on duplexes formed by the palladated oligonucleotide **ON1pp‐Pd**, as the metal‐free counterpart **ON1pp** did not absorb at this wavelength. The melting profiles (Figure S35—S39 in the Supporting Information) were remarkably similar, with monophasic sigmoidal hypochromicity on increasing temperature. Melting temperatures in most cases fell between 56 °C and 57 °C and within error limits of each other. **ON1pp‐Pd**●**ON2c** gave a somewhat lower value, ≈54 °C, but still much higher than any of the melting temperatures determined at 260 nm. In other words, the pyrenylpyridine palladacycle moiety is involved in some transition that takes place over a temperature range where the duplex is already largely denatured. Stacking within single‐stranded **ON1pp‐Pd** (in contrast to its duplexes) seems possible in light of previous reports on interactions of small‐molecule palladacycles with DNA.^[^ ^51^, ^52^, ^53^, ^54^, ^55^ ^]^ If stacking of the pyrenylpyridine palladacycle is, indeed, favorable within single‐stranded **ON1pp‐Pd** but impossible within its duplexes, the loss of this interaction on hybridization could at least partly explain the unusually low melting temperatures at 260 nm.

### 2.4 Fluorescence Melting Profiles

To establish appropriate excitation and emission wavelengths for fluorescence‐based melting studies, the excitation and emission spectra were first recorded for the relatively abundant pyrenylpyridine *C*‐nucleoside **4** (rather than the precious oligonucleotides **ON1pp** and **ON1pp‐Pd**) in MeOH (Figure S40 in the Supporting Information), yielding *λ*_ex_ = 345 nm and *λ*_em_ = 425 nm. Prior to the melting experiments, these values were further elaborated on the single‐stranded oligonucleotide **ON1pp** (data not shown). The emission maximum was very similar to the one determined for **4** (419 nm), but the excitation maximum had shifted to 365 nm. Comparable red‐shift on going from a dimer to an oligonucleotide system has been reported previously^[^ ^56^ ^]^ and attributed to the fluorophore being sandwiched between neighboring nucleobases.^[^ ^57^ ^]^ Accordingly, an excitation wavelength of 365 nm was used in all of the fluorescence melting experiments. Sample preparation was otherwise identical to the UV melting experiments, but concentration of the oligonucleotides was reduced to 50 nM. In addition to the various duplexes, single‐stranded **ON1pp** and **ON1pp‐Pd** were also included in the study. Comparison of the absorption and emission spectra of compound **4** and oligonucleotides **ON1pp** and **ON1pp‐Pd** is presented in Figure S41–S43 of the Supporting Information.

**Figure 2:**
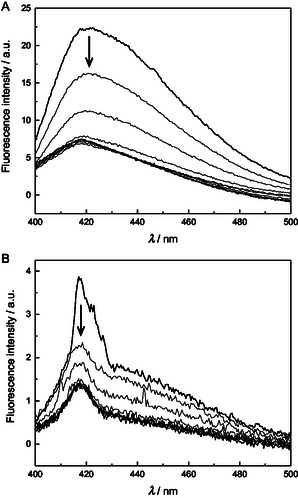
Emission spectra for duplexes A) **ON1pp**●**ON2c** and B) **ON1pp‐Pd**●**ON2c**, recorded at 10 °C intervals between 10 and 90 °C; [oligonucleotides] = 50 nm; pH = 7.4 (20 mM cacodylate buffer); *I* = 0.10 M (adjusted with NaClO_4_); *λ*_ex_ = 365 nm. The spectra obtained at the extreme temperatures are represented by thicker lines and the direction of change on increasing temperature indicated by arrows.

At the low end of the temperature range (10 °C), all of the metal‐free duplexes, as well as single‐stranded **ON1pp**, showed prominent fluorescence emission with a maximum around 419 nm (spectra for **ON1pp**●**ON2c** are presented in **Figure** **2**A as an illustrative example and the others in Figure S44–S49 in the Supporting Information). For each spectrum, the maximum emission intensity was obtained as the height of a bigaussian peak function fitted to the experimental data. The emission exhibited a considerable decrease on increasing the temperature from 10 °C to 50 °C, followed by a slight recovery at higher temperatures. In contrast to the UV melting profiles recorded at 260 nm, none of the curves (Figure S56 in the Supporting Information) showed appreciable saturation at the low end of the temperature range, precluding determination of melting temperatures.

Emission spectra of the palladated duplexes and the single‐stranded **ON1pp‐Pd** were strikingly different from those of the metal‐free counterparts (spectra for **ON1pp‐Pd**●**ON2c** are presented in Figure 2B as an illustrative example and the others in Figure S50–S55 in the Supporting Information). The intensity was much lower and the sum of two bigaussian functions was required for an adequate fitting of the experimental data, suggesting the presence of two different emissive species. Perhaps the most likely explanation is that the palladacyclic oligonucleotide **ON1pp‐Pd** contains a small amount of its metal‐free counterpart **ON1pp** as an impurity. Temperature‐dependence of the emission (Figure S57 in the Supporting Information) was qualitatively similar to that of the metal‐free oligonucleotides, except that the recovery at high temperatures was in most cases too modest to be seen clearly and emission of single‐stranded **ON1pp‐Pd** was largely insensitive to changes in temperature.

Intercalation of a pyrene derivative into double‐helical DNA leads to quenching of its fluorescence, owing to reduced polarity of the environment.^[^ ^58^, ^59^, ^60^ ^]^ In the case of oligonucleotides bearing a pyrene moiety at the end of a flexible linker, this phenomenon manifests itself as hybridization‐induced decrease of fluorescence emission.^[^ ^56^, ^61^, ^62^ ^]^ In contrast, oligonucleotides bearing a pyrene moiety directly appended to a nucleobase are often more emissive within a double helix than as single strands, as intercalation without disruption of the base stack is not possible.^[^ ^34^, ^35^, ^63^, ^64^ ^]^ Based on the observed thermal decrease of fluorescence emission, as well as the structure of the *C*‐nucleoside **4**, the latter scenario seems likely also in the present case. In other words, the relatively low emission at the high end of the temperature range would correspond to single‐stranded **ON1pp** or **ON1pp‐Pd** and the relatively high (and sequence‐dependent) emission at the low end of the temperature range to a double helix formed on hybridization with one of the complementary oligonucleotides. Apart from the modest but measurable temperature‐dependence of the fluorescence emission of single‐stranded **ON1pp**, all results are consistent with this interpretation. However, it should be pointed out that, based on the UV melting profiles recorded at 260 nm, the palladated duplexes (unlike their metal‐free counterparts) are not fully formed even at 10 °C. Accordingly, the measured emissions in these cases actually represent an average between those of the single‐ and double‐stranded species.

**Figure 3:**
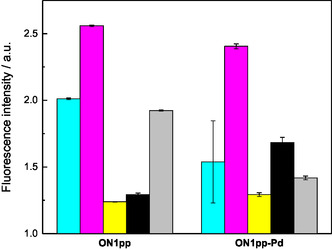
Relative fluorescence intensities of the duplexes formed by oligonucleotides **ON1pp** and **ON1pp‐Pd** with the complementary oligonucleotides **ON2a** (cyan), **ON2c** (magenta), **ON2g** (yellow), **ON2t** (black), and **ON2s** (gray) at 10 °C; [oligonucleotides] = 50 nm; pH = 7.4 (20 mM cacodylate buffer); *I* = 0.10 M (adjusted with NaClO_4_); *λ*_ex_ = 365 nm; and *λ*_em_ = 418 nm. The error bars represent standard error of fitting one (**ON1pp**) or two (**ON1pp‐Pd**) bigaussian functions to the emission spectra recorded at 10 °C.

Fluorescence emissions of all duplexes at 10 °C are summarized in **Figure** **3**. The duplex placing cytosine opposite to the modified residue stands out in both series and especially with **ON1pp‐Pd**. Likewise, pairing of either pyrenylpyridine or its palladacycle with guanine resulted in the lowest emission. Overall, hybridization with the palladacyclic oligonucleotide **ON1pp‐Pd** offered somewhat better discrimination between the canonical nucleobases. Based on qualitative differences between the UV melting profiles obtained at 355 nm, Pd(II)‐mediated base pairing might be involved but the data at hand does not allow a meaningful proposal of specific binding modes.

Fluorescence quantum yields of the duplexes were determined by comparison of the absorbancies and integrated emission spectra of the samples with those of a known fluorophore (pyrene). Low absorbance of the duplex samples makes the values rather uncertain but a pattern of higher quantum yields for the metal‐free duplexes (especially **ON1pp●ON2a** and **ON1pp●ON2c**) is nonetheless evident. All quantum yields are summarized in Table S1 in the Supporting Information.

### 2.5 CD Melting Profiles

The increase of fluorescence on hybridization, observed with both **ON1pp** and **ON1pp‐Pd**, suggests that the pyrenylpyridine moiety is not neatly intercalated within the base stack buy might actually distort its geometry. On the other hand, formation of a double helix – even a distorted one – could induce chirality to the pyrenylpyridine moiety. These possibilities were explored through CD spectropolarimetric measurements over the same temperature range as used in the UV and fluorescence melting studies (10–90 °C). The samples were identical to those of the UV melting experiments.

**Figure 4:**
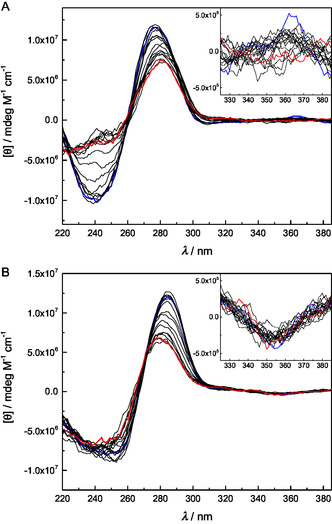
CD spectra of duplexes A) **ON1pp**•**ON2c** and B) **ON1pp‐Pd**•**ON2c**, recorded at 5 °C intervals between 10 and 90 °C; [oligonucleotides] = 1.0 μM; pH = 7.4 (20 mM cacodylate buffer); *I* = 0.10 M (adjusted with NaClO_4_). The spectra obtained at 10 °C and 90 °C are represented by thicker blue and red lines, respectively. The inset shows a magnification of the minor Cotton effect assigned to the pyrenylpyridine moiety.

At 10 °C, CD spectra of the metal‐free duplexes were characteristic of B‐type double helices, with prominent negative and positive Cotton effects at 240 and 275 nm, respectively (spectra for **ON1pp**●**ON2c** are presented in **Figure** **4**A as an illustrative example and the others in Figure S58–S62 in the Supporting Information). In most cases, a weak positive Cotton effect around 360 nm could also be observed, consistent with induced chirality of the pyrenylpyridine moiety. The spectra of the palladacyclic duplexes were roughly similar, but the major negative Cotton effect extended further toward shorter wavelengths; the major positive one had red‐shifted to 285 nm and the minor one around 360 nm was negative (spectra for **ON1pp‐Pd**●**ON2c** are presented in Figure 4B as an illustrative example and the others in Figure S63–S67 in the Supporting Information). With all samples, both of the major Cotton effects diminished on increasing temperature, indicating unwinding of the double helix. This diminution was more prominent with the palladacyclic duplexes than with their metal‐free counterparts, especially in the case of the positive Cotton effect. Interestingly, the minor Cotton effect assigned to the pyrenylpyridine moiety exhibited opposite trends on increasing temperature, decreasing and eventually disappearing with the metal‐free duplexes but remaining relatively unchanged with the palladacyclic ones.

**Figure 5:**
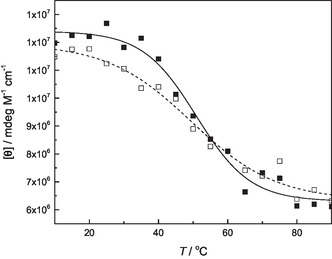
CD melting profiles of duplexes **ON1pp**●**ON2c** (□ and dashed line) and **ON1pp‐Pd**●**ON2c** (▪ and solid line); [oligonucleotides] = 1.0 μM; pH = 7.4 (20 mM cacodylate buffer); *I* = 0.10 M (adjusted with NaClO_4_); *λ* = 275 (for **ON1pp**●**ON2c**) / 285 (for **ON1pp‐Pd**●**ON2c**) nm.

Plotting the molar ellipticity of the major positive Cotton effect as a function of temperature afforded monophasic sigmoidal curves with all duplexes (those for **ON1pp**●**ON2c** and **ON1pp‐Pd**●**ON2c** are presented in **Figure** **5** as an illustrative example and the others in Figure S68—S77 in the Supporting Information). In contrast to the UV melting profiles obtained at 260 nm, the melting temperatures of these curves were similar between the metal‐free and palladacyclic duplexes, ranging from 42 °C to 55 °C. With the metal‐free duplexes, the CD melting temperatures were 3–14 °C higher than their UV counterparts—a significant difference but perhaps understandable given that the two methods are based on different (albeit related) phenomena. With the palladacyclic duplexes, however, the difference is much greater and the two melting temperatures may not even stem from the same transformation. Accordingly, the UV melting at 260 nm would mainly reflect dissociation of the duplex and the CD melting loss of helicity of the constituent single strands. Finally, it is interesting to note that CD melting temperatures of the palladacyclic duplexes align reasonably well with the UV melting temperatures determined at 425 °C (a summary of all melting temperatures is provided in Table S1 in the Supporting Information).

The magnitude and sign of the induced Cotton effect of pyrene varies depending on its position within the double helix.^[^ ^65^ ^]^ For example, intercalation has been reported to give rise to both positive^[^ ^66^ ^]^ and negative^[^ ^56^, ^67^ ^]^ Cotton effects depending on the way the pyrene moiety is tethered to the oligonucleotide and thus its orientation relative to the base pairs.^[^ ^68^ ^]^ Similarly, both negative and positive induced Cotton effects have also been attributed to pyrene moieties residing in the minor groove.^[^ ^69^, ^70^ ^]^ In the present case, the major groove would appear a more likely location given the structure of the *C*‐nucleoside **4**, but, to the best of our knowledge, no CD data for such a system have been reported. In any case, the persistence of the weak induced Cotton effect around 360 nm over the entire temperature range suggests that the conformation of the pyrenylpyridine palladacycle moiety is largely similar in single‐stranded **ON1pp‐Pd** and its duplexes with the various complementary oligonucleotides. With the metal‐free oligonucleotide **ON1pp**, on the other hand, the corresponding signal was gradually lost on increasing temperature. The different structures of the base surrogate of the *C*‐nucleoside residue provide a possible explanation for this difference. In pyren‐1‐ylpyridine, rotation around the bond connecting the two rings is inherently free but can be hindered by the double‐helical environment, giving rise to induced CD. Thermal denaturation of the double helix would lift these constraints, resulting in free rotation of the pyrene moiety and thus loss of the induced CD. Cyclopalladation of pyren‐1‐ylpyridine locks the mutual orientation of the two rings, making it less sensitive to such external stimuli.

## 3 Conclusion

When placed in the middle of a short oligodeoxynucleotide, a pyren‐1‐ylpyridine C‐nucleoside does not promote hybridization with complementary sequences and its palladacyclic derivative actually retards it. This finding, along with the increased fluorescence emission of both the metal‐free and palladacyclic duplexes, argues against intercalation of the pyrene moiety within the double helix. However, fluorescent emission of the duplexes showed a clear dependence on the nucleobase opposite to the pyrenylpyridine residue and especially the corresponding palladacycle. In the latter case, Pd(II)‐mediate base pairing might be involved. While its origin eludes the present study, the observed fluorescence‐based discrimination between canonical nucleobases is encouraging and makes metallacyclic nucleobase surrogates an interesting candidate as a sensor moiety for identification of SNPs.

## 4 Experimental

4.1

4.1.1

##### General methods

All solvents involved in organic synthesis were of reagent grade and dried over activated 3 or 4 Å molecular sieves. The reactions were monitored by thin layer chromatography (TLC) performed on Merck 60 (silica gel F254) plates. TLC plates were visualized by exposure to ultraviolet light. Purification of the products was accomplished using flash column chromatography on silica gel (230–400 mesh). ^1^H, ^13^C and ^31^P NMR spectra were recorded in deuterated solvents on Bruker BioSpin 500 or 600 MHz spectrometers. Chemical shifts (δ, ppm) are quoted relative to the internal residual solvent signals (for ^1^H and ^13^C) or external orthophosphoric acid (for ^31^P). Mass spectra were recorded on a Bruker micrOTOF‐Q mass spectrometer. Freshly distilled triethylamine was used for preparation of the HPLC elution buffers.

##### 6‐(Pyren‐1‐yl)‐3‐[3,5‐di‐O‐(Tert‐Butyldimethylsilyl)‐2‐Deoxy‐β‐D‐Erythro‐Pentofuranosyl]pyridine (**3**)

Compound **2** (1.101 g, 2.19 mmol, 1 eq.), K_2_CO_3_ (756 mg, 5.48 mmol, 2.5 eq.) Pd(PPh_3_)_4_ (253 mg, 0.21 mmol, 0.1 eq.), and PyB(OH)_2_ (1.34 g, 5.48 mmol, 2.5 eq.) were suspended in a mixture of MeOH (16.7 mL) and H_2_O (3.3 mL). The mixture was refluxed for 15 h under an argon atmosphere, after which it was filtered through a plug of celite and concentrated under reduced pressure. The residue was purified by silica gel flash chromatography (EtOAc:hexane, stepwise gradient from 0:100 to 70:30, *v*/*v*), affording 786 mg (57%) of the desired product **3**. ^1^H NMR (500 MHz, CDCl_3_): δ = 8.88 (d, *J* = 1.8 Hz, 1H, pyridine‐H2), 8.42 (d, *J* = 9.3 Hz, 1H, pyrene‐H10), 8.28 (d, *J* = 7.9 Hz, 1H, pyrene‐H3), 8.23 (d, *J* = 7.6 Hz, 1H, pyrene‐H6), 8.21 (d, *J* = 7.6 Hz, 1H, pyrene‐H8), 8.19 (d, J = 7.9 Hz, 1H, pyrene‐H2), 8.14 (br, 2H, pyrene‐H4 & ‐H5), 8.10 (d, *J* = 9.3 Hz, 1H, pyrene‐H9), 8.05 (t, *J* = 7.6 Hz, 1H, pyrene‐H7), 7.98 (dd, *J*_*1*_ = 8.0 Hz, *J*_*2*_ = 2.1 Hz, 1H, pyridine‐H4), 7.73 (d, *J* = 8.0 Hz, 1H, pyridine‐H5), 5.36 (dd, *J*_1_ = 10.4 Hz, *J*_2_ = 5.3 Hz, 1H, H1′), 4.57 (m, 1H, H3′), 4.10 (m, 1H, H4′), 3.88 (dd, *J*_*1*_ = 10.8 Hz, *J*_*2*_ = 3.7 Hz, 1H, H5′), 3.75 (dd, *J*_*1*_ = 10.8 Hz, *J*_*2*_ = 5.7 Hz, 1H, H5”), 2.32 (ddd, *J*_1_ = 12.6 Hz, *J*_2_ = 5.4 Hz, *J*_3_ = 1.1 Hz, 1H, H2′), 2.92 (ddd, *J*_1_ = 12.7 Hz, *J*_2_ = 10.7, *J*_3_ = 5.4 Hz, 1H, H2”), 1.00 (s, 9H, Si‐CCH_3_), 0.97 (s, 9 H, Si‐CCH_3_), 0.19—0.15 (m, 12H, Si‐CH_3_). ^13^C NMR (125 MHz, CDCl_3_): δ = 158.7 (pyridine‐C6), 147.9 (pyridine‐C2), 136.0 (pyridine‐C3), 135.6 (pyrene‐C1), 134.3 (pyridine‐C4), 131.42 (pyrene‐C3a), 131.39 (pyrene‐C5a), 130.9 (pyrene‐C8a), 128.7 (pyrene‐C10a), 128.0 (pyrene‐C9), 127.9 (pyrene‐C4 or ‐C5), 127.6 (pyrene‐C2), 127.4 (pyrene‐C5 or ‐C4), 126.0 (pyrene‐C7), 125.4 (pyridine‐C5), 125.3 (pyrene‐C6), 125.1 (pyrene‐C3a^1^), 125.0 (pyrene‐C8), 124.92 (pyrene‐C10), 124.86 (pyrene‐C5a^1^), 124.8 (pyrene‐C3), 88.4 (C4′), 77.9 (C1′), 74.5 (C3′), 63.9 (C5′), 44.2 (C2′), 26.0 (Si‐CCH_3_), 25.9 (Si‐CCH_3_), 18.4 (Si‐CCH_3_), 18.1 (Si‐CCH_3_), −4.5 (Si‐CH_3_), −4.6 (Si‐CH_3_), −5.3 (Si‐CH_3_), −5.4 (Si‐CH_3_). HRMS (ESI^+^‐TOF): *m*/*z* calcd for [C_38_H_49_KNO_3_Si_2_]: 662.2883; found: 662.2897 [M + K]^+^.

##### 6‐(Pyren‐1‐yl)‐3‐(2‐Deoxy‐β‐D‐Erythro‐Pentofuranosyl)pyridine (**4**)

Et_3_N·3HF (698 μL, 4.1 mmol, 5 eq.) was added to a solution of compound **3** (510 mg, 0.81 mmol, 1 eq.) in dry THF (5 mL). The mixture was stirred at room temperature for 16 h, after which solvents were removed under reduced pressure. The residue was dissolved in MeOH (5 mL), and 1 M aq. NaOH (13 mL) was added to neutralize the mixture. The solvents were removed under reduced pressure and the residue purified by silica gel flash chromatography (MeOH:CH_2_Cl_2_, 1:9, *v*/*v*), affording the desired product **4** (311 mg, 96%) as a white solid. ^1^H NMR (500 MHz, CD_3_OD): δ = 8.81 (d, *J* = 1.5 Hz, 1H, pyridine‐H2), 8.26 (d, *J* = 7.9 Hz, 1H, pyrene‐H3), 8.22 (d, *J* = 7.6 Hz, 1H, pyrene‐H6), 8.19 (d, *J* = 7.6 Hz, 1H, pyrene‐H8), 8.15 (d, *J* = 9.2 Hz, 1H, pyrene‐H10), 8.13—8.09 (m, 2H, pyrene‐H4 & ‐H5), 8.08—8.04 (m, 3H, pyridine‐H4 & pyrene‐H2 & ‐H9), 8.02 (t, *J* = 7.6 Hz, 1H, pyrene‐H7), 7.71 (d, *J* = 8.1 Hz, 1H, pyridine‐H5), 5.34 (dd, *J*_1_ = 10.5 Hz, *J*_2_ = 5.5 Hz, 1H, H1′), 4.44 (m, 1H, H3′), 4.06 (m, 1H, H4′), 3.77 (m, 2 H, H5′ & H5”), 2.38 (ddd, *J*_1_ = 13.0 Hz, *J*_2_ = 5.6 Hz, *J*_3_ = 1.3 Hz, 1H, H2′), 2.11 (ddd, *J*_1_ = 13.0 Hz, *J*_2_= 10.7 Hz, *J*_3_ = 5.8 Hz, 1H, H2”). ^13^C NMR (125 MHz, CD_3_OD): δ = 158.2 (pyridine‐C6), 146.8 (pyridine‐C2), 136.8 (pyridine‐C3), 135.2 (pyridine‐C4), 134.7 (pyrene‐C1), 131.6 (pyrene‐C3a), 131.4 (pyrene‐C5a), 130.8 (pyrene‐C8a), 128.5 (pyrene‐C10a), 127.8 (pyrene‐C9), 127.7 (pyrene‐C4 or ‐C5), 127.1 (pyrene‐C2), 126.9 (pyrene‐C5 or ‐C4), 126.0 (pyrene‐C7), 125.6 (pyridine‐C5), 125.2 (pyrene‐C6), 124.9 (pyrene‐C8), 124.6 (pyrene‐C3a^1^), 124.40 (pyrene‐C5a^1^), 124.38 (pyrene‐C3), 124.1 (pyrene‐C10), 88.2 (C4′), 77.7 (C1′), 73.1 (C3′), 62.6 (C5′), 43.5 (C2′). HRMS (ESI^+^‐TOF): *m*/*z* calcd for [C_26_H_21_NNaO_3_]: 418.1414; found: 418.1455 [M + Na]^+^.

##### 66‐(Pyren‐1‐yl)‐3‐[5‐O‐(4,4′‐Dimethoxytrityl)‐2‐Deoxy‐β‐D‐Erythro‐Pentofuranosyl]pyridine (**5**)

Compound **4** (245 mg, 6.18 mmol, 1 eq.) was dissolved in anhydrous pyridine (5 mL). DMTrCl (230 mg, 6.80 mmol, 1.1 eq.) was added and the resulting mixture stirred at room temperature for 48 h, after which it was concentrated under reduced pressure. The residue was dissolved in CH_2_Cl_2_ (80 mL) and washed with saturated aq. NaHCO_3_ (100 mL). The organic layer was dried with Na_2_SO_4_ and evaporated to dryness. The was residue purified on a silica gel column (Et_3_N:EtOAc:hexane, 2:58:40, *v*/*v*/*v*) affording 320 mg (74%) of the desired product **5**. ^1^H NMR (500 MHz, CDCl_3_): δ = 8.86 (d, *J* = 1.8 Hz, 1H, pyridine‐H2), 8.40 (d, *J* = 9.3 Hz, 1H, pyrene‐H10), 8.28 (d, *J* = 7.9 Hz, 1H, pyrene‐H3), 8.23 (d, *J* = 7.6 Hz, 1H, pyrene‐H6), 8.21 (d, *J* = 7.5 Hz, 1H, pyrene‐H8), 8.17 (d, *J* = 7.9 Hz, 1H, pyrene‐H2), 8.13 (m, 2 H, pyrene‐H4 & ‐H5), 8.09 (d, *J* = 9.3 Hz, 1H, pyrene‐H9), 8.04 (t, *J* = 7.6 Hz, 1H, pyrene‐H7), 7.93 (dd, *J*_*1*_ = 8.0 Hz, *J*_*2*_ = 2.1 Hz, 1H, pyridine‐H4), 7.71 (d, *J* = 8.0 Hz, 1H, pyridine‐H5), 7.51 (m, 2 H, Ph‐H2 & ‐H6), 7.39 (m, 4 H, MeOPh‐H2 & ‐H6), 7.32 (m, 2 H, Ph‐H3 & ‐H5), 7.24 (m, 1H, Ph‐H4), 6.87 (m, 4 H, MeOPh‐H3 & ‐H5), 5.37 (dd, *J*_*1*_ = 10.1 Hz, *J*_*2*_ = 5.6 Hz, 1H, H1′), 4.55 (m, 1H, H3′), 4.19 (m, 1H, H4′), 3.80 (s, 6H, OCH_3_), 3.44 (dd, *J*_*1*_ = 9.9 Hz, *J*_*2*_ = 4.5 Hz, 1H, H5′), 3.36 (dd, *J*_*1*_ = 9.9 Hz, *J*_*2*_ = 5.4 Hz, 1H, H5”), 2.43 (ddd, *J*_1_ = 13.0 Hz, *J*_2_ = 5.6 Hz, *J*_3_ = 1.5 Hz, 1H, H2′), 2.21 (ddd, *J*_1_ = 13.0 Hz, Hz, *J*_2_ = 10.2, *J*_3_ = 5.9 Hz, 1H, H2”). ^13^C NMR (125 MHz, CDCl_3_): δ = 158.8 (pyridine‐C6), 158.6 (MeOPh‐C4), 147.9 (pyridine‐C2), 144.8 (Ph‐C1), 136.0 (MeOPh‐C1), 135.6 (pyrene‐C1), 135.5 (pyridine‐C3), 134.2 (pyridine‐C4), 131.4 (pyrene‐C3a & ‐C5a), 130.9 (pyrene‐C8a), 130.13 (MeOPh‐C2 & ‐C6), 130.11 (MeOPh‐C2 & ‐C6), 128.6 (pyrene‐C10a), 128.2 (Ph‐C2 & ‐C6), 128.0 (pyrene‐C9 & pyrene‐C4 or ‐C5), 127.9 (Ph‐C3 & ‐C5), 127.6 (pyrene‐C2), 127.4 (pyrene‐C5 or ‐C4), 126.9 (Ph ‐C4), 126.0 (pyrene‐C7), 125.41 (pyridine‐C5), 125.35 (pyrene‐C6), 125.1 (pyrene‐C3a^1^ & pyrene‐C8), 124.9 (pyrene‐C10), 124.84 (pyrene‐C5a^1^), 124.78 (pyrene‐C3), 113.2 (MeOPh‐C3 & ‐C5), 86.6 (C4′), 86.4 (Ar_3_C), 77.8 (C1′), 74.7 (C3′), 64.5 (C5′), 55.2 (OCH_3_), 45.8, 43.7 (C2′). HRMS (ESI^+^‐TOF): *m*/*z* calcd for [C_47_H_39_NNaO_5_]: 720.2720; found: 720.2713 [M + Na]^+^.

##### 6‐(Pyren‐1‐yl)‐3‐{3‐O‐[(2‐Cyanoethoxy)‐(N,N‐Diisopropylamino)phosphinyl]‐5‐O‐(4,4′‐Dimethoxytrityl)‐2‐Deoxy‐β‐D‐Erythro‐Pentofuranosyl}pyridine (**1**)

Compound **5** (154 mg, 2.30 mmol, 1 eq.) was dissolved in anhydrous CH_2_Cl_2_ (1.5 mL). Et_3_N (150 μL, 11.6 mmol, 5 eq.) and 2‐cyanoethyl‐*N*,*N*‐diisopropylchlorophosphoramidite (57 μL, 2.42 mmol, 1.1 eq.) were added and the reaction mixture stirred under N_2_ at room temperature for 150 min. The reaction was quenched by addition of saturated aq. NaHCO_3_ (50 mL) and the resulting mixture extracted with CH_2_Cl_2_ (50 mL). The organic layer was dried over Na_2_SO_4_ and evaporated to dryness. The residue was purified by flash column chromatography on oven dried silica gel using N_2_ gas to drive the eluent (Et_3_N:EtOAC:hexane, 2:55:48, *v*/*v*/*v*) to yield 160 mg (80%) of the desired product **1** as a mixture of *R*_p_ and *S*_p_ diastereomers. ^1^H NMR (500 MHz, CDCl_3_): δ = 8.92 (m, 1H, pyridine‐H2), 8.42 (d, *J* = 9.3 Hz, 1H, pyrene‐H10), 8.28 (d, *J* = 7.9 Hz, 1H, pyrene‐H3), 8.24 (d, *J* = 7.6 Hz, 1H, pyrene‐H6), 8.21 (d, *J* = 7.7 Hz, 1H, pyrene‐H8), 8.18 (d, *J* = 7.9 Hz, 1H, pyrene‐H2), 8.14 (m, 2H, pyrene‐H4 & ‐H5), 8.09 (d, *J* = 9.3 Hz, 1H, pyrene‐H9), 8.05 (t, *J* = 7.6 Hz, 1H, pyrene‐H7), 7.98 (m, pyridine‐H4), 7.73 (m, 1H, pyridine‐H5), 7.52 (m, 2H, Ph‐H2 & ‐H6), 7.40 (m, 4H, MeOPh‐H2 & ‐H6), 7.33 (m, 2H, Ph‐H3 & ‐H5), 7.23 (m, 1H, Ph‐H4), 6.89 (m, 4H, MeOPh‐H3 & ‐H5), 5.39 (m, 1H, H1′), 4.68 (m, 1H, H3′), 4.38 (m, 1H, H4′), 3.92 (m, 1H, POCH_2_), 3.84 (m, 1H, POCH_2_), 3.81 (s, 3H, OCH_3_), 3.80 (s, 3H, OCH_3_), 3.68 (m, 2H, PNCH), 3.42 (m, 2H, H5′ & H5”), 2.67 (t, *J* = 6.3 Hz, 1H, CH_2_CN), 2.67—2.51 (m, 1H, H2′), 2.53 (t, *J* = 6.4 Hz, 1H, CH_2_CN), 2.24 (m, 1H, H2”), 1.25 (m, 9H, NCHCH_3_), 1.18 (d, *J* = 6.7 Hz, 3H, NCHCH_3_). ^13^C NMR (125 MHz, CDCl_3_): δ = 158.9 (pyridine‐C6), 158.8 (pyridine‐C6), 158.5 (MeOPh‐C4), 148.02 (pyridine‐C2), 148.01 (pyridine‐C2), 144.9 (Ph‐C1), 144.8 (Ph‐C1), 136.1 (MeOPh‐C1), 136.0 (MeOPh‐C1), 135.60 (pyrene‐C1), 135.56 (pyrene‐C1), 135.40 (pyridine‐C3), 135.38 (pyridine‐C3), 134.30 (pyridine‐C4), 134.27 (pyridine‐C4), 131.4 (pyrene‐C3a & ‐C5a), 130.9 (pyrene‐C8a), 130.21 (MeOPh‐C2 & ‐C6), 130.18 (MeOPh‐C2 & ‐C6), 128.7 (pyrene‐C10a), 128.33 (Ph‐C2 & ‐C6), 128.28 (Ph‐C2 & ‐C6), 128.0 (pyrene‐C9 & pyrene‐C4 or ‐C5), 127.9 (Ph‐C3 & ‐C5), 127.6 (pyrene‐C2), 127.4 (pyrene‐C5 or ‐C4), 126.9 (Ph ‐C4), 126.8 (Ph ‐C4), 126.0 (pyrene‐C7), 125.44 (pyridine‐C5), 125.41 (pyridine‐C5), 125.36 (pyrene‐C6), 125.35 (pyrene‐C6), 125.1 (pyrene‐C3a^1^ & pyrene‐C8), 124.93 (pyrene‐C10), 124.90 (pyrene‐C10), 124.85 (pyrene‐C5a^1^), 124.81 (pyrene‐C3), 117.6 (CN), 117.5 (CN), 113.2 (MeOPh‐C3 & ‐C5), 86.4 (d, *J* = 3.9 Hz, C4′), 86.28 (Ar_3_C), 86.26 (Ar_3_C), 86.0 (d, *J* = 5.8 Hz, C4′), 78.21 (C1′), 78.17 (C1′), 76.4 (d, *J* = 17.4 Hz, C3′), 75.9 (d, *J* = 16.5 Hz, C3′), 64.20 (C5′), 64.16 (C5′), 58.4 (d, *J* = 6.0 Hz, POCH_2_), 58.3 (d, *J* = 6.3 Hz, POCH_2_), 55.25 (OCH_3_), 55.24 (OCH_3_), 43.3 (d, *J* = 4.9 Hz, PNCH), 43.24 (d, *J* = 4.9 Hz, PNCH), 43.17 (C2′), 43.1 (C2′), 24.8—24.4 (m, NCHCH_3_), 20.5 (d, *J* = 7.1 Hz, CH_2_CN), 20.3 (d, *J* = 6.9 Hz, CH_2_CN). ^31^ P NMR (202 MHz, CDCl_3_, diastereoisomeric mixture): δ = 148.1, 147.9. HRMS (ESI^+^‐TOF): *m*/*z* calcd for [C_56_H_56_N_3_NaO_6_P]: 920.3804; found: 920.3766 [M + Na]^+^.

##### Oligonucleotide Synthesis

The pyrenylpyridine‐modified oligonucleotide **ON1pp** was synthesized using phosphoramidite chemistry on an Applied Biosystems incorporated 3400 automated DNA/RNA synthesizer. Coupling time for the pyrenylpyridine *C*‐nucleoside building block **1** was extended to 300 s. Based on the trityl response, all couplings proceeded with normal efficiency. Cleavage from the solid support and deprotection of phosphate and base moieties were achieved by incubation in 33% aqueous ammonia at 55 °C overnight. For synthesis of the cyclopalladated oligonucleotide **ON1pp‐Pd**, **ON1pp** (30 nmol) and Li_2_PdCl_4_ (1.5 mg, 10 μmol) were dissolved in 0.1 M aqueous NaOAc (100 μL). The mixture was shaken at 55 °C overnight. The product mixtures were purified by RP‐HPLC on a Hypersil ODS C18 column (250 × 4.6 mm, 5 μm) eluting with a linear gradient of MeCN (5%–40% over 20 min, flow rate = 1.0 mL min^−1^) in a 50 mM triethylammonium acetate buffer (pH = 7.0). The palladacyclic oligonucleotide **ON1pp‐Pd** eluted as several peaks, possibly owing to formation of intrastrand crosslinks through coordination of Pd(II) to one of the many nitrogen ligands present. Such crosslinks would be expected to dissociate on hybridization of **ON1pp‐Pd** with a complementary oligonucleotide. The purified oligonucleotides were characterized by ESI‐TOF mass spectrometry (HPLC traces presented in Figure S10 and mass spectra in Figure S11 and S12 of the Supporting information) and quantified by UV spectrophotometry using molar absorptivities calculated by an implementation of the nearest‐neighbors method. Molar absorptivity of the pyrenylpyridine *C*‐nucleoside **4** at 260 nm was determined to be 3300 L mol^−1^ cm^−1^ in MeOH (UV spectrum presented in Figure S13 in the Supporting Information). This value was used for calculation of the molar absorptivity of both **ON1pp** and **ON1pp‐Pd**, as solubility issues precluded measurements on the cyclopalladated derivative of **4**.

##### UV Melting Experiments

UV melting profiles were recorded on PerkinElmer Lambda 35 UV/vis spectrophotometer equipped with a Peltier temperature control unit. The samples were prepared by diluting stock solutions of the appropriate oligonucleotides to a 1.0 μM final concentration with 20 mM cacodylate buffer (pH = 7.4), the ionic strength of which was adjusted to 0.10 M with NaClO_4_. The samples were placed in quartz cuvettes with 10 mm optical path length and their absorbance at 260, 365, and 425 (only with palladacyclic duplexes) nm was recorded at 0.5 °C intervals between 10 °C and 90 °C. Three heating and cooling ramps of 0.5 °C min^−1^ were run at each wavelength. Where applicable, melting temperatures were determined by plotting the absorbance as a function of temperature and fitting the data points to Equation (1).

(1) $$A/A_{90}=a_{\text{low}}+b_{\text{low}}T+\frac{\left(a_{\text{high}}+b_{\text{high}}T\right)-\left(a_{\text{low}}+b_{\text{low}}T\right)}{1+10^{\left(T_{m}-T\right)h}}$$

*A* and *A*_90_ are measured absorbances at a given temperature and at 90 °C. *a*_low_ and *b*_low_ are the offset and slope of the “low‐temperature” baseline and *a*_high_ and *b*_high_ the corresponding values for the “high‐temperature” baseline. These terms were included to allow the baselines to deviate from horizontality. In cases where the data did not allow reliable fitting, the slope parameters were fixed at zero value. *T*_m_ is the melting temperature and *h* the slope of the melting curve at the melting temperature. The more conventional approach of determining the melting temperature as the maximum (or, in the case of profiles obtained at 425 nm, the minimum) of the first‐derivative curve of the melting profile yielded mostly comparable results. However, in cases where the “high‐temperature” baseline was very sloped (notably duplexes **ON1pp●ON2a** and **ON1pp●ON2g** at 260 nm), melting temperatures obtained by the latter method were systematically higher. Larger and less systematic differences were observed when the melting took place at a very low temperature (palladacyclic duplexes at 260 nm) or over a very wide temperature range (palladacyclic duplexes at 425 nm). All melting temperatures are summarized in Table S1 of the Supporting Information.

##### CD Melting Experiments

CD spectra were recorded on an Applied Photophysics Chirascan spectropolarimeter equipped with a Peltier temperature control unit. The samples and the cuvettes were identical to the ones used in the UV melting experiments. Spectra were acquired over a wavelength range of 200–400 nm and a temperature range of 10–90 °C, at 5 °C intervals. At each temperature, the samples were allowed to equilibrate for 120 s before acquisition. Melting temperatures were determined by plotting the molar ellipticity at 275 (for metal‐free duplexes) or 285 nm (for palladacyclic duplexes) as a function of temperature and fitting the data points to Equation (1), as described above for the UV melting experiments.

##### Fluorescence Melting Experiments

Fluorescence emission spectra were recorded on a Cary Eclipse fluorescence spectrometer over a wavelength range of 400–500 nm, the excitation wavelength being 365 nm. The excitation and emission slits were set to 5 nm, photomultiplier tube voltage to 600 V, and scan rate to 120 nm min^−1^. The samples were otherwise identical to those of the UV and CD experiments but the oligonucleotide concentration was reduced to 50 nM. The emission intensity was quantified by fitting one (for metal‐free duplexes) or two (for palladacyclic duplexes) bigaussian peak functions to the emission spectra. Quantum yields were estimated by a method employed previously with related duplexes,^[^ ^64^ ^]^ namely by comparison with a known reference material (pyrene) using Equation (2).

(2) $$\Phi_{\text{duplex}}=\Phi_{\text{referemce}}\frac{Int_{\text{duplex}}}{Int_{\text{reference}}}\frac{1-10^{-A_{\text{reference}}}}{1-10^{-A_{\text{duplex}}}}\frac{n_{\text{duplex}}^{2}}{n_{\text{reference}}^{2}}$$

Φ_reference_ is the quantum yield of pyrene in cyclohexane (0.32). *Int*_duplex_ and *Int*_reference_ are integrals of the emission spectra of the duplex and pyrene samples and *A*_duplex_ and *A*_reference_ their absorbancies at the excitation wavelength (365 nm for the duplexes and 317 nm for pyrene). *n*_duplex_ and *n*_reference_ are the refractive indices of the solvents (water and cyclohexane, respectively).

## Conflict of Interest

The authors declare no conflict of interest.

## Supporting information

Supplementary Material

## Data Availability

The data that support the findings of this study are available in the supplementary material of this article.
