# Supplementary material for: Hybridization Probes Featuring a Pyrenylpyridine C‐Nucleoside or Its Palladacycle as a Fluorescent Sensor Moiety
Source: Chembiochem. 2025 Jul 21;26(17):e202500474. doi: 10.1002/cbic.202500474 (PMC12442211; doi:10.1002/cbic.202500474)
Supplement: Supplementary file 1 — Supplementary Material [file CBIC-26-e202500474-s001.pdf]

Contents

Figure S1. ^{1}H NMR spectrum of compound 3.	S3
Figure S2. ^{13}C NMR spectrum of compound 3.	S5
Figure S3. ^{1}H NMR spectrum of compound 4.	S7
Figure S4. ^{13}C NMR spectrum of compound 4.	S9
Figure S5. ^{1}H NMR spectrum of compound 5.	S11
Figure S6. ^{13}C NMR spectrum of compound 5.	S13
Figure S7. ^{1}H NMR spectrum of compound 1.	S15
Figure S8. ^{13}C NMR spectrum of compound 1.	S17
Figure S9. ^{31}P NMR spectrum of compound 1.	S19
Figure S10. HPLC traces of oligonucleotides ON1pp and ON1pp-Pd.	S20
Figure S11. Mass spectrum of oligonucleotide ON1pp.	S21
Figure S12. Mass spectrum of oligonucleotide ON1pp-Pd.	S22
Figure S13. UV spectrum of compound 4.	S23
Figure S14. Normalized UV spectra of oligonucleotides ON1pp and ON1pp-Pd.	S23
Figure S15. UV melting profile and fitting to Equation 1 for duplex ON1pp•ON2a; λ = 260 nm.	S24
Figure S16. UV melting profile and fitting to Equation 1 for duplex ON1pp•ON2c; λ = 260 nm.	S25
Figure S17. UV melting profile and fitting to Equation 1 for duplex ON1pp•ON2g; λ = 260 nm.	S25
Figure S18. UV melting profile and fitting to Equation 1 for duplex ON1pp•ON2t; λ = 260 nm.	S25
Figure S19. UV melting profile and fitting to Equation 1 for duplex ON1pp•ON2s; λ = 260 nm.	S26
Figure S20. UV melting profile and fitting to Equation 1 for duplex ON1pp-Pd•ON2a; λ = 260 nm.	S26
Figure S21. UV melting profile and fitting to Equation 1 for duplex ON1pp-Pd•ON2c; λ = 260 nm.	S27
Figure S22. UV melting profile and fitting to Equation 1 for duplex ON1pp-Pd•ON2g; λ = 260 nm.	S27
Figure S23. UV melting profile and fitting to Equation 1 for duplex ON1pp-Pd•ON2t; λ = 260 nm.	S28
Figure S24. UV melting profile and fitting to Equation 1 for duplex ON1pp-Pd•ON2s; λ = 260 nm.	S28
Figure S25. UV melting profile for duplex ON1pp•ON2a; λ = 355 nm.	S29
Figure S26. UV melting profile for duplex ON1pp•ON2c; λ = 355 nm.	S29
Figure S27. UV melting profile for duplex ON1pp•ON2g; λ = 355 nm.	S30
Figure S28. UV melting profile for duplex ON1pp•ON2t; λ = 355 nm.	S30
Figure S29. UV melting profile for duplex ON1pp•ON2s; λ = 355 nm.	S31
Figure S30. UV melting profile for duplex ON1pp-Pd•ON2a; λ = 355 nm.	S31
Figure S31. UV melting profile for duplex ON1pp-Pd•ON2c; λ = 355 nm.	S32
Figure S32. UV melting profile for duplex ON1pp-Pd•ON2g; λ = 355 nm.	S32
Figure S33. UV melting profile for duplex ON1pp-Pd•ON2t; λ = 355 nm.	S33
Figure S34. UV melting profile for duplex ON1pp-Pd•ON2s; λ = 355 nm.	S33
Figure S35. UV melting profile and fitting to Equation 1 for duplex ON1pp-Pd•ON2a; λ = 425 nm.	S34
Figure S36. UV melting profile and fitting to Equation 1 for duplex ON1pp-Pd•ON2c; λ = 425 nm.	S34
Figure S37. UV melting profile and fitting to Equation 1 for duplex ON1pp-Pd•ON2g; λ = 425 nm.	S35
Figure S38. UV melting profile and fitting to Equation 1 for duplex ON1pp-Pd•ON2t; λ = 425 nm.	S35
Figure S39. UV melting profile and fitting to Equation 1 for duplex ON1pp-Pd•ON2s; λ = 425 nm.	S36
Figure S40. Excitation and emission spectra of compound 4.	S36
Figure S41. Absorption and emission spectra of compound 4.	S37
Figure S42. Absorption and emission spectra of oligonucleotide ON1pp.	S38
Figure S43. Absorption and emission spectra of oligonucleotide ON1pp-Pd.	S38
Figure S44. Emission spectra and fitting to a bigaussian peak function for oligonucleotide ON1pp.	S39
Figure S45. Emission spectra and fitting to a bigaussian peak function for duplex ON1pp•ON2a.	S39
Figure S46. Emission spectra and fitting to a bigaussian peak function for duplex ON1pp•ON2c.	S40
Figure S47. Emission spectra and fitting to a bigaussian peak function for duplex ON1pp•ON2g.	S40
Figure S48. Emission spectra and fitting to a bigaussian peak function for duplex ON1pp•ON2t.	S41
Figure S49. Emission spectra and fitting to a bigaussian peak function for duplex ON1pp•ON2s.	S41

Contents (continued)

Figure S50. Emission spectra and fitting to a bigaussian peak function for oligonucleotide ON1pp-Pd. S42
Figure S51. Emission spectra and fitting to a bigaussian peak function for duplex ON1pp-Pd•ON2a. S42
Figure S52. Emission spectra and fitting to a bigaussian peak function for duplex ON1pp-Pd•ON2c. S43
Figure S53. Emission spectra and fitting to a bigaussian peak function for duplex ON1pp-Pd•ON2g. S43
Figure S54. Emission spectra and fitting to a bigaussian peak function for duplex ON1pp-Pd•ON2t. S44
Figure S55. Emission spectra and fitting to a bigaussian peak function for duplex ON1pp-Pd•ON2s. S44
Figure S56. Fluorescence melting profiles for single-stranded ON1pp and duplexes ON1pp•ON2a, ON1pp•ON2c, ON1pp•ON2g, ON1pp•ON2t and ON1pp•ON2s. S45
Figure S57. Fluorescence melting profiles for single-stranded ON1pp-Pd and duplexes ON1pp-Pd•ON2a, ON1pp-Pd•ON2c, ON1pp-Pd•ON2g, ON1pp-Pd•ON2t and ON1pp-Pd•ON2s. S45
Figure S58. CD spectra of duplex ON1pp•ON2a. S46
Figure S59. CD spectra of duplex ON1pp•ON2c. S46
Figure S60. CD spectra of duplex ON1pp•ON2g. S47
Figure S61. CD spectra of duplex ON1pp•ON2t. S47
Figure S62. CD spectra of duplex ON1pp•ON2s. S48
Figure S63. CD spectra of duplex ON1pp-Pd•ON2a. S48
Figure S64. CD spectra of duplex ON1pp-Pd•ON2c. S49
Figure S65. CD spectra of duplex ON1pp-Pd•ON2g. S49
Figure S66. CD spectra of duplex ON1pp-Pd•ON2t. S50
Figure S67. CD spectra of duplex ON1pp-Pd•ON2s. S50
Figure S68. CD melting profile and fitting to Equation 1 for duplex ON1pp•ON2a; λ = 275 nm. S51
Figure S69. CD melting profile and fitting to Equation 1 for duplex ON1pp•ON2c; λ = 275 nm. S51
Figure S70. CD melting profile and fitting to Equation 1 for duplex ON1pp•ON2g; λ = 275 nm. S52
Figure S71. CD melting profile and fitting to Equation 1 for duplex ON1pp•ON2t; λ = 275 nm. S52
Figure S72. CD melting profile and fitting to Equation 1 for duplex ON1pp•ON2s; λ = 275 nm. S53
Figure S73. CD melting profile and fitting to Equation 1 for duplex ON1pp-Pd•ON2a; λ = 285 nm. S53
Figure S74. CD melting profile and fitting to Equation 1 for duplex ON1pp-Pd•ON2c; λ = 285 nm. S54
Figure S75. CD melting profile and fitting to Equation 1 for duplex ON1pp-Pd•ON2g; λ = 285 nm. S54
Figure S76. CD melting profile and fitting to Equation 1 for duplex ON1pp-Pd•ON2t; λ = 285 nm. S55
Figure S77. CD melting profile and fitting to Equation 1 for duplex ON1pp-Pd•ON2s; λ = 285 nm. S55
Table S1. UV and CD melting temperatures and selected photophysical properties of the various metal-free and palladacyclic duplexes. S56

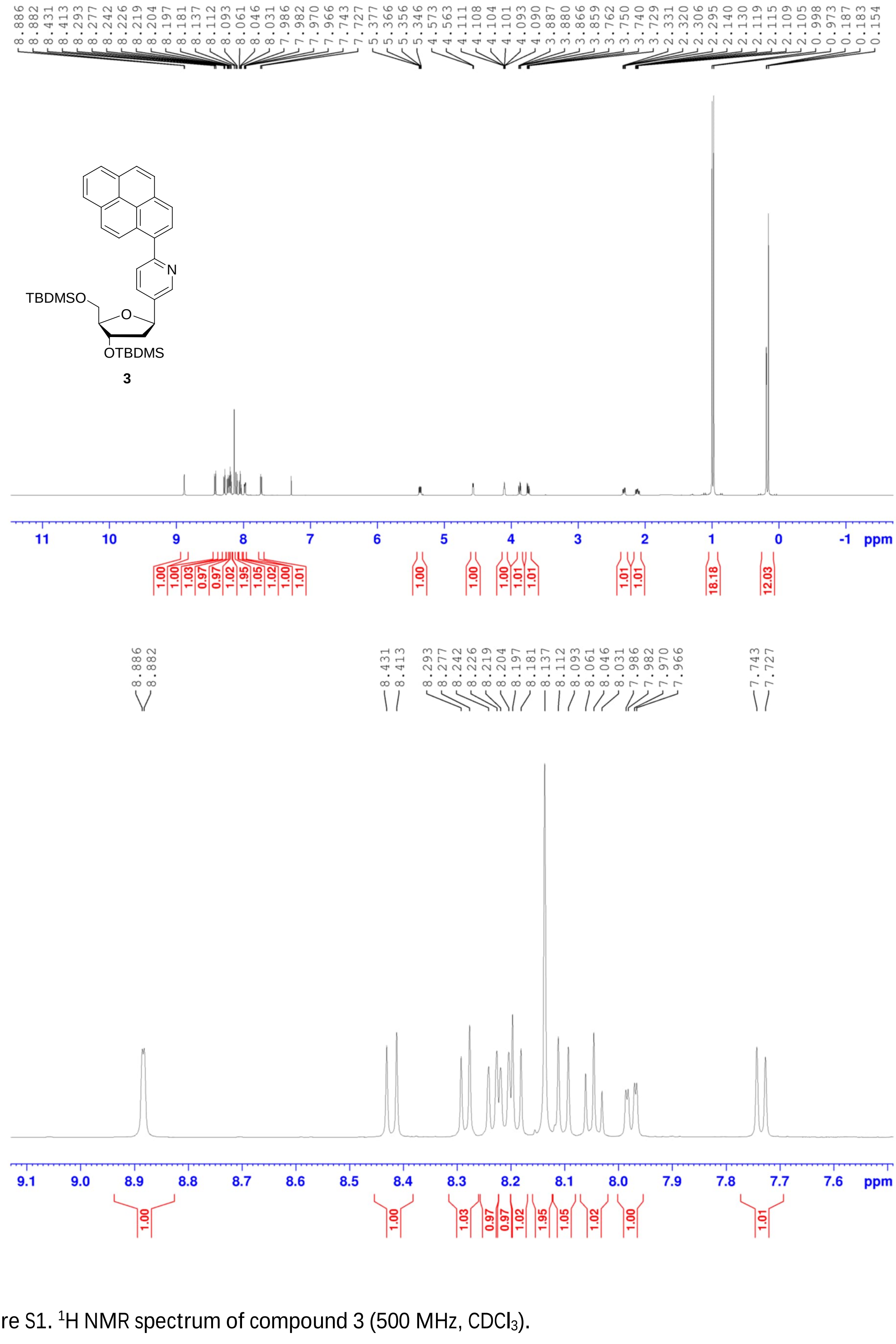

Figure S1. ^{1}H NMR spectrum of compound 3 (500 MHz, $CDCl_3$).

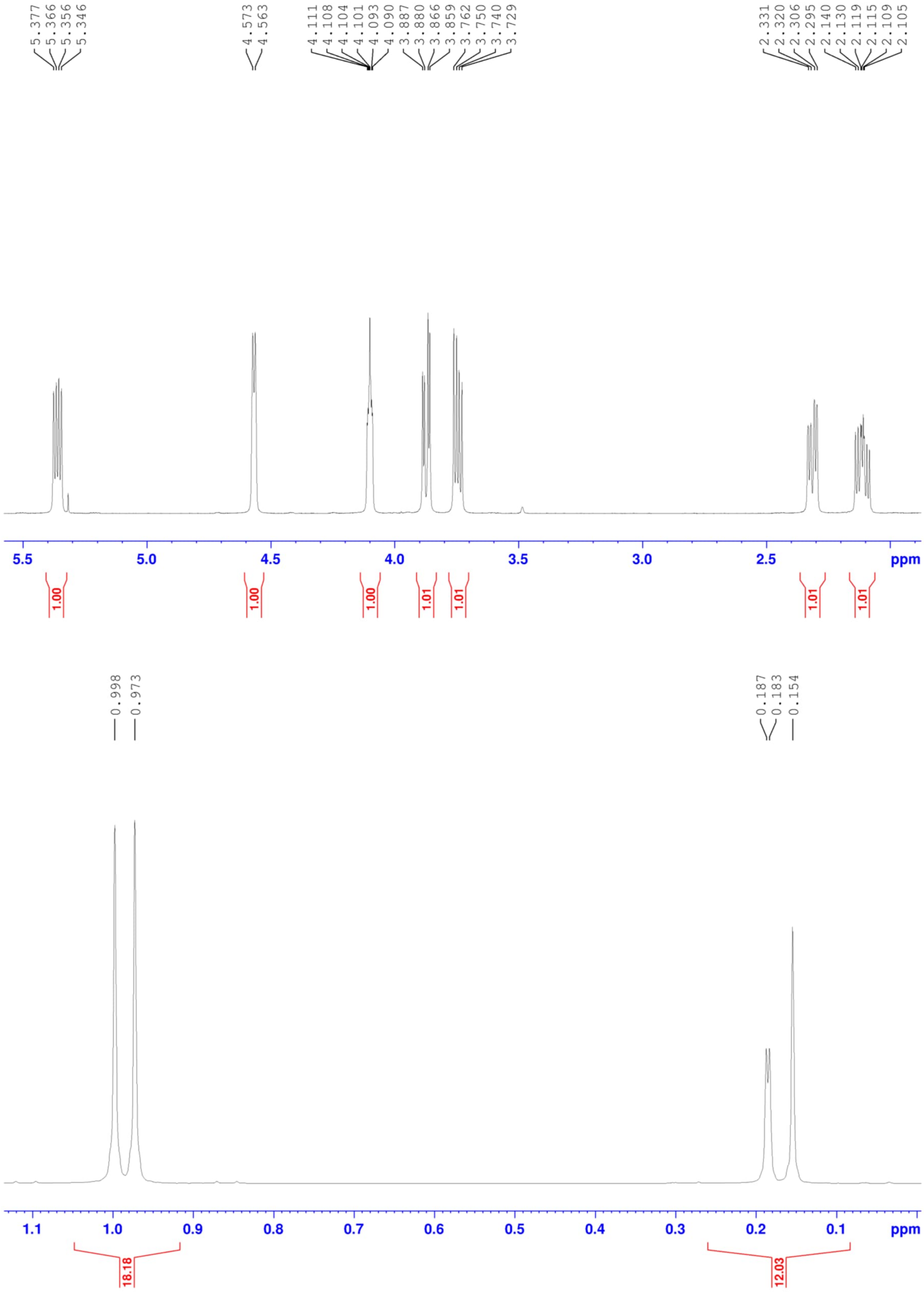

Figure S1 (continued). ^{1}H NMR spectrum of compound 3 (500 MHz, $CDCl_3$).

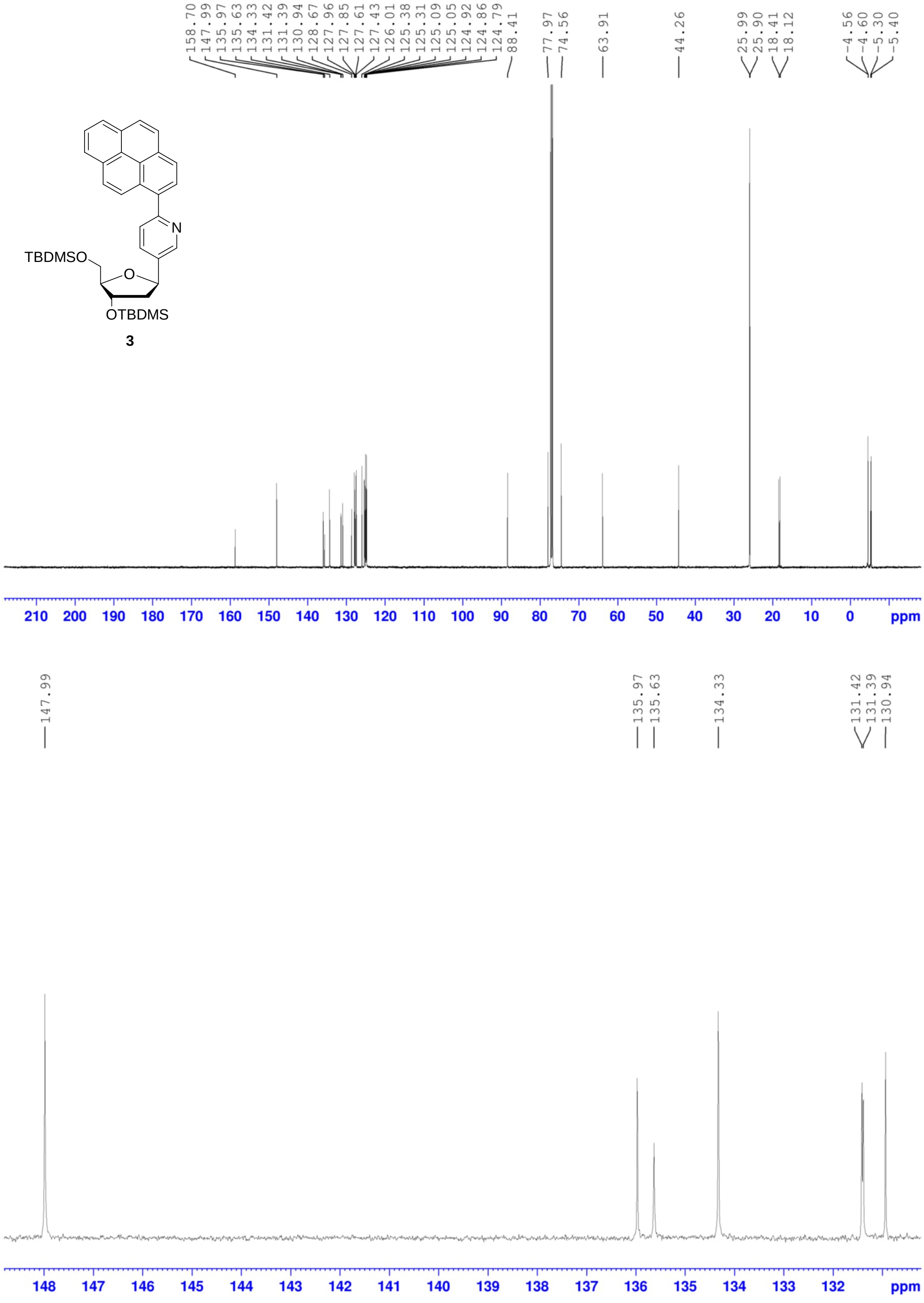

Figure S2. ^{13}C NMR spectrum of compound 3 (125 MHz, $CDCl_3$).

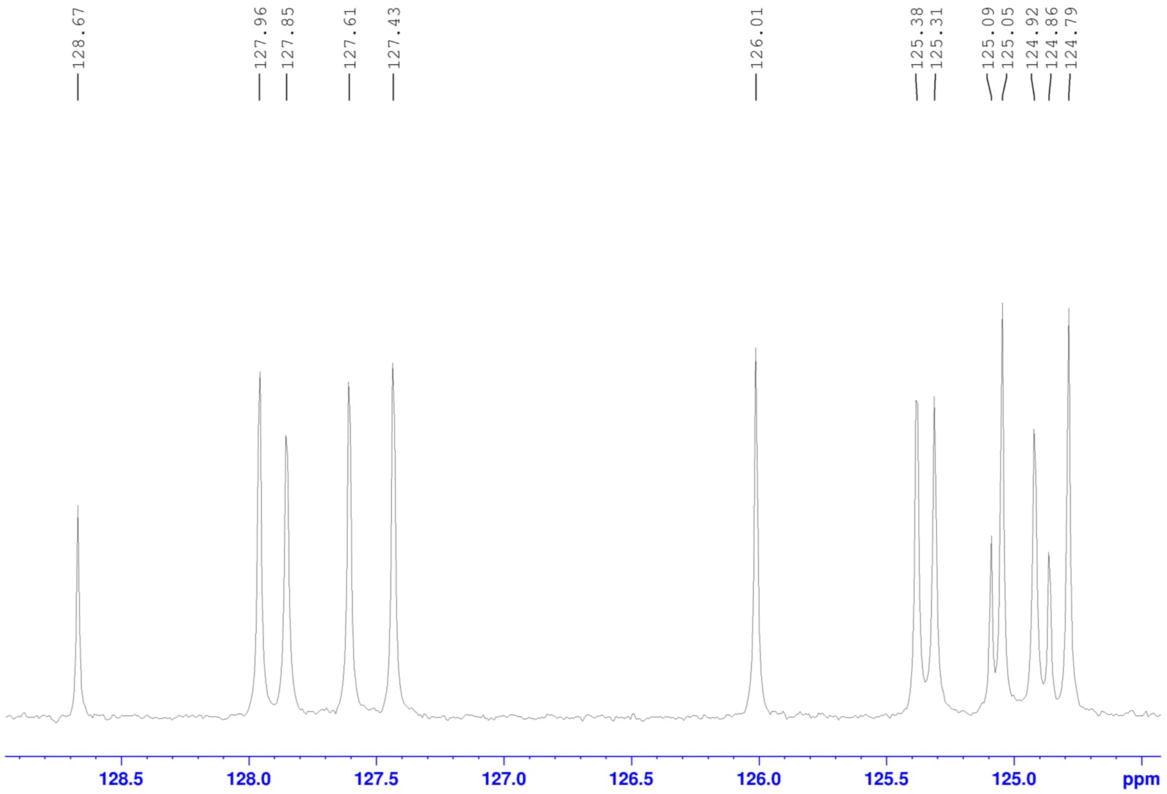

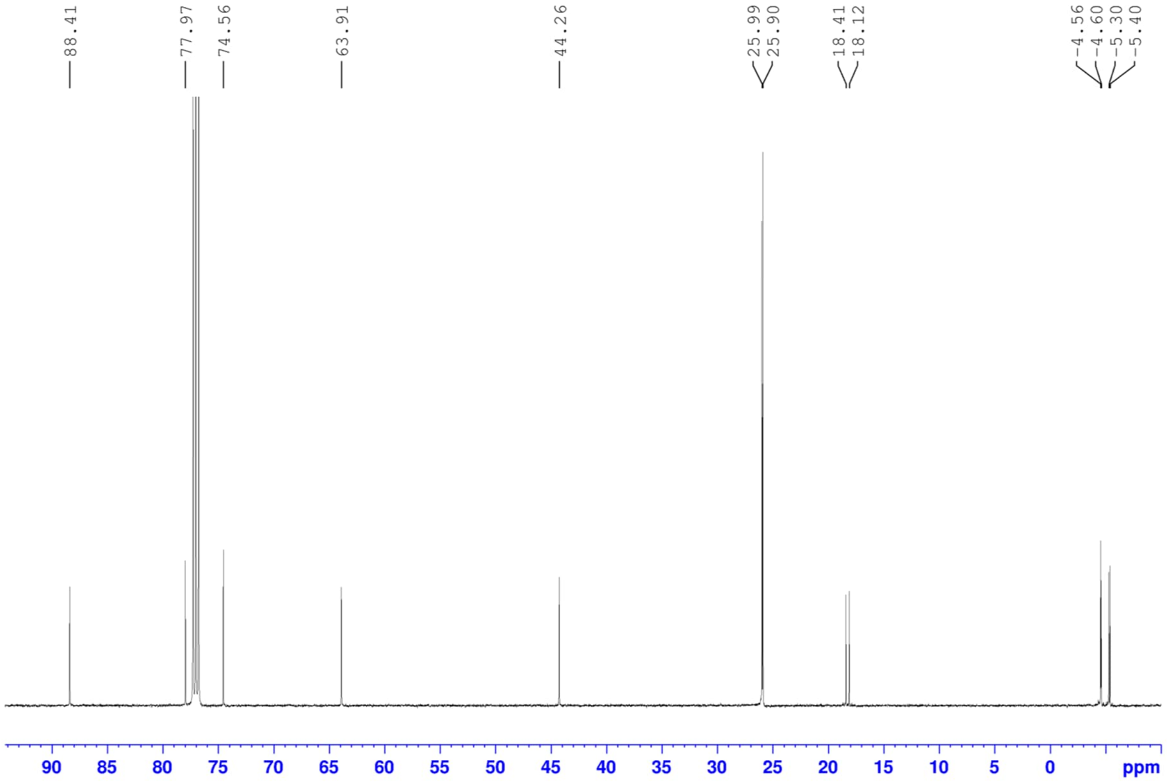

Figure S2 (continued). ^{13}C NMR spectrum of compound 3 (125 MHz, $CDCl_3$).

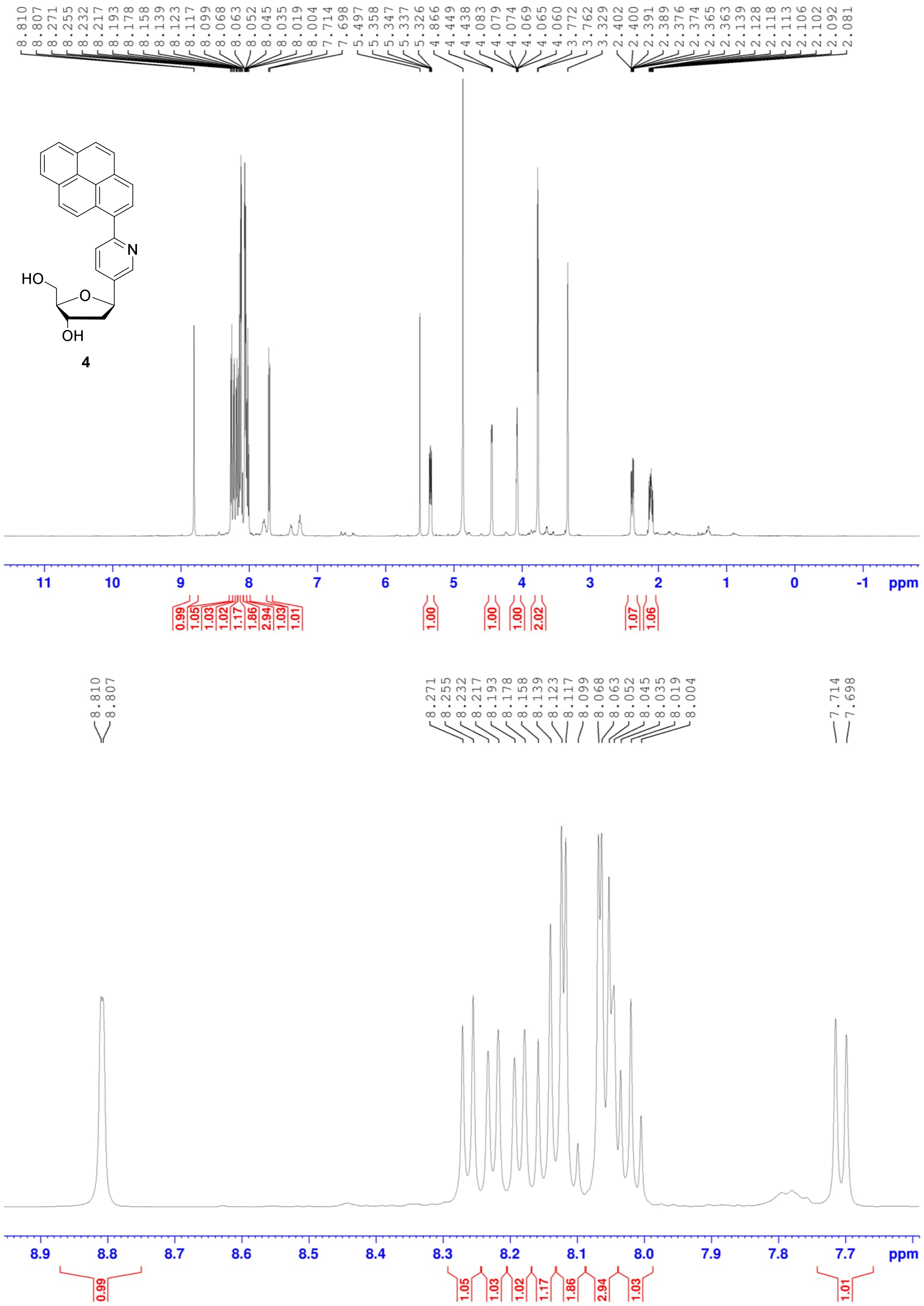

Figure S3. ^{1}H NMR spectrum of compound 4 (500 MHz, CD_3OD).

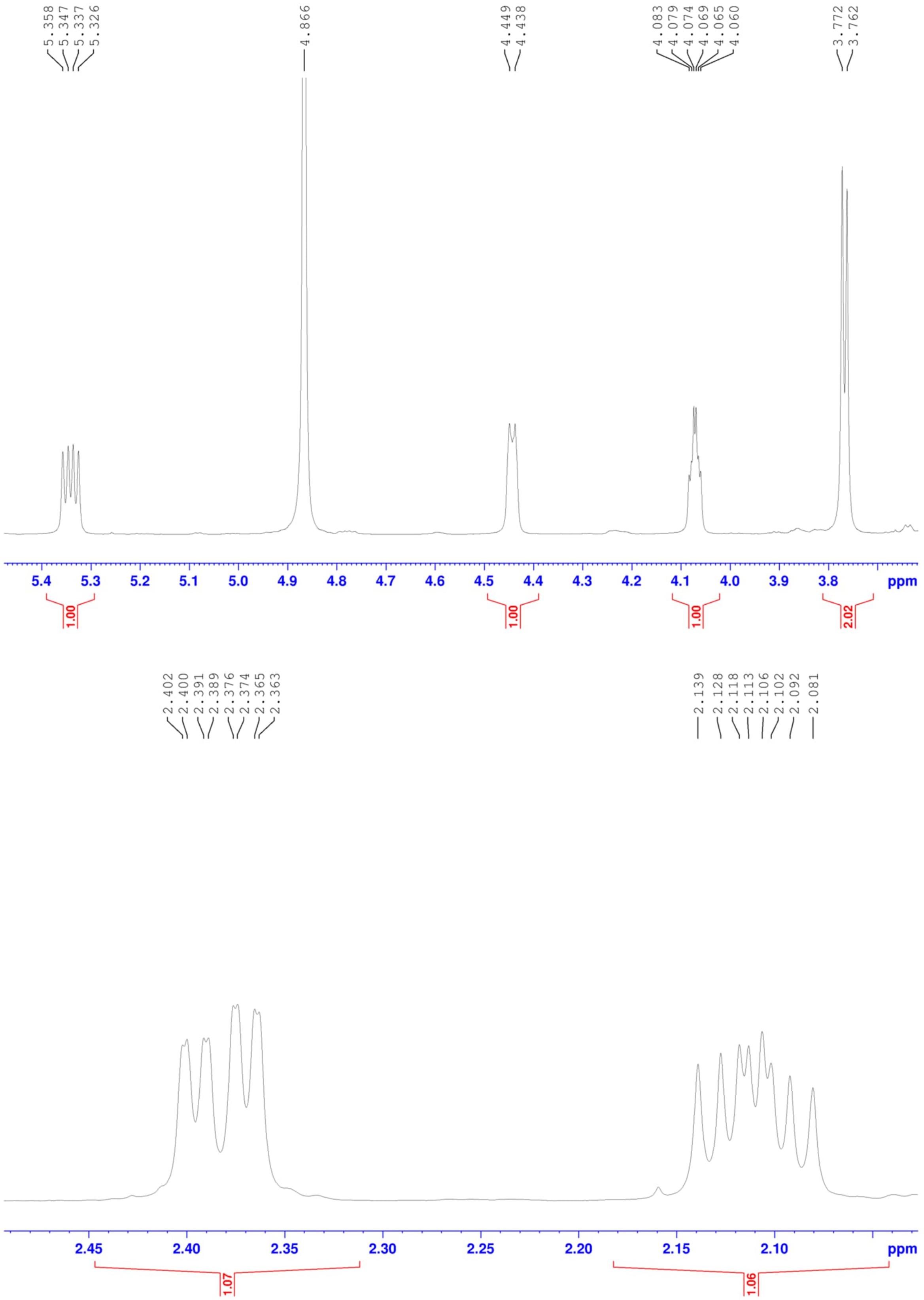

Figure S3 (continued). ^{1}H NMR spectrum of compound 4 (500 MHz, CD_3OD).

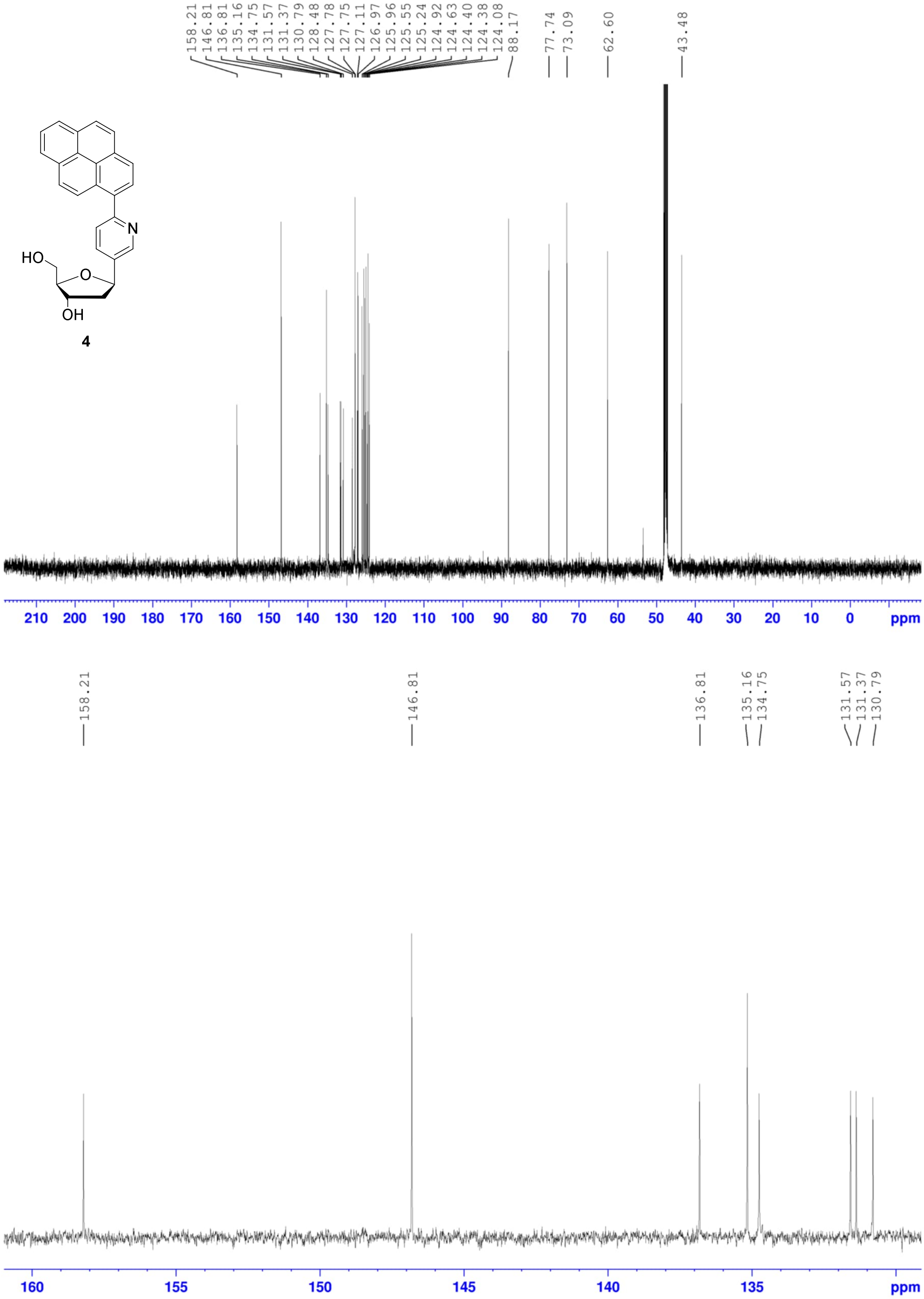

Figure S4. ^{13}C NMR spectrum of compound 4 (125 MHz, CD_3OD).

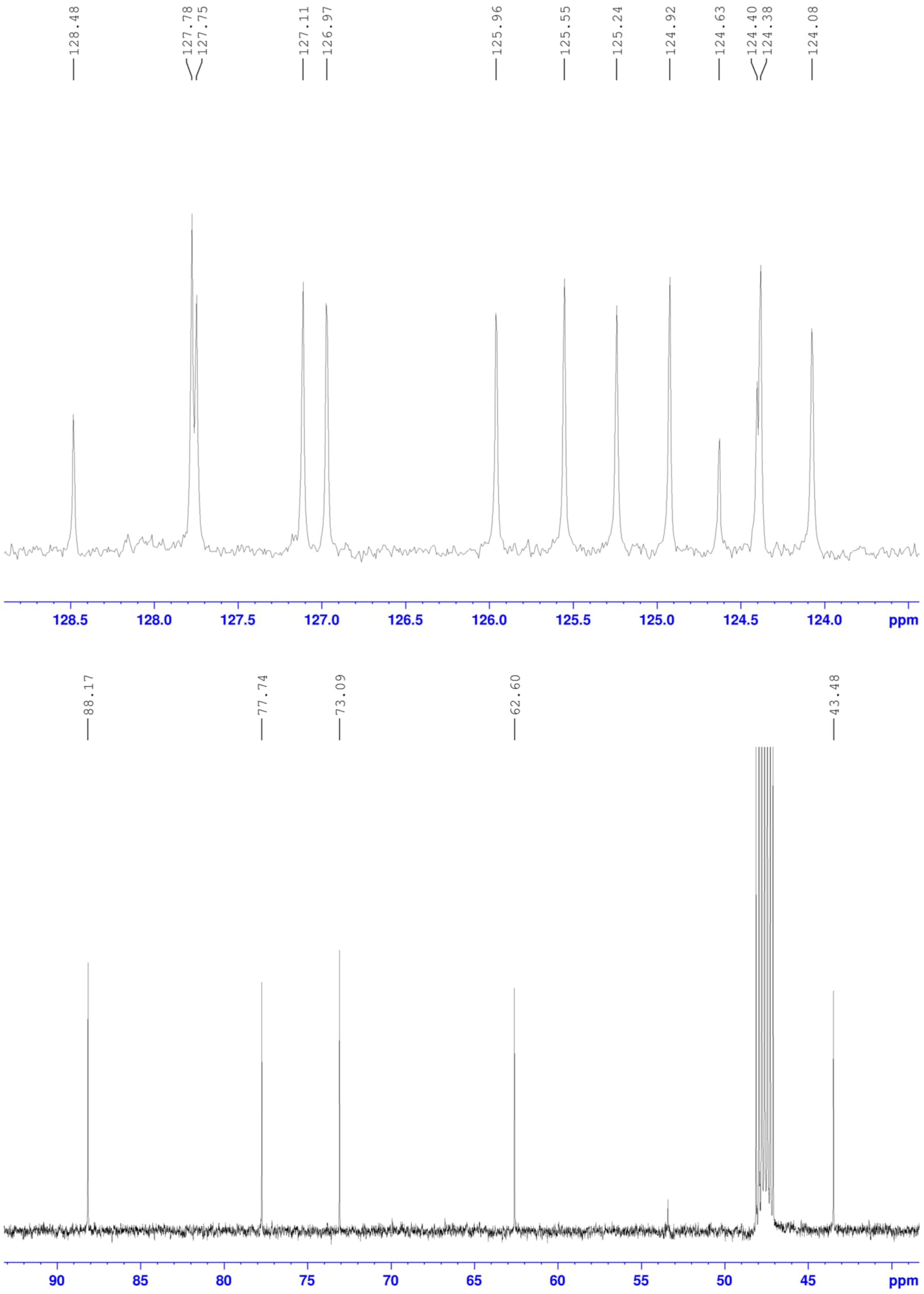

Figure S4 (continued). ^{13}C NMR spectrum of compound 4 (125 MHz, CD_3OD).

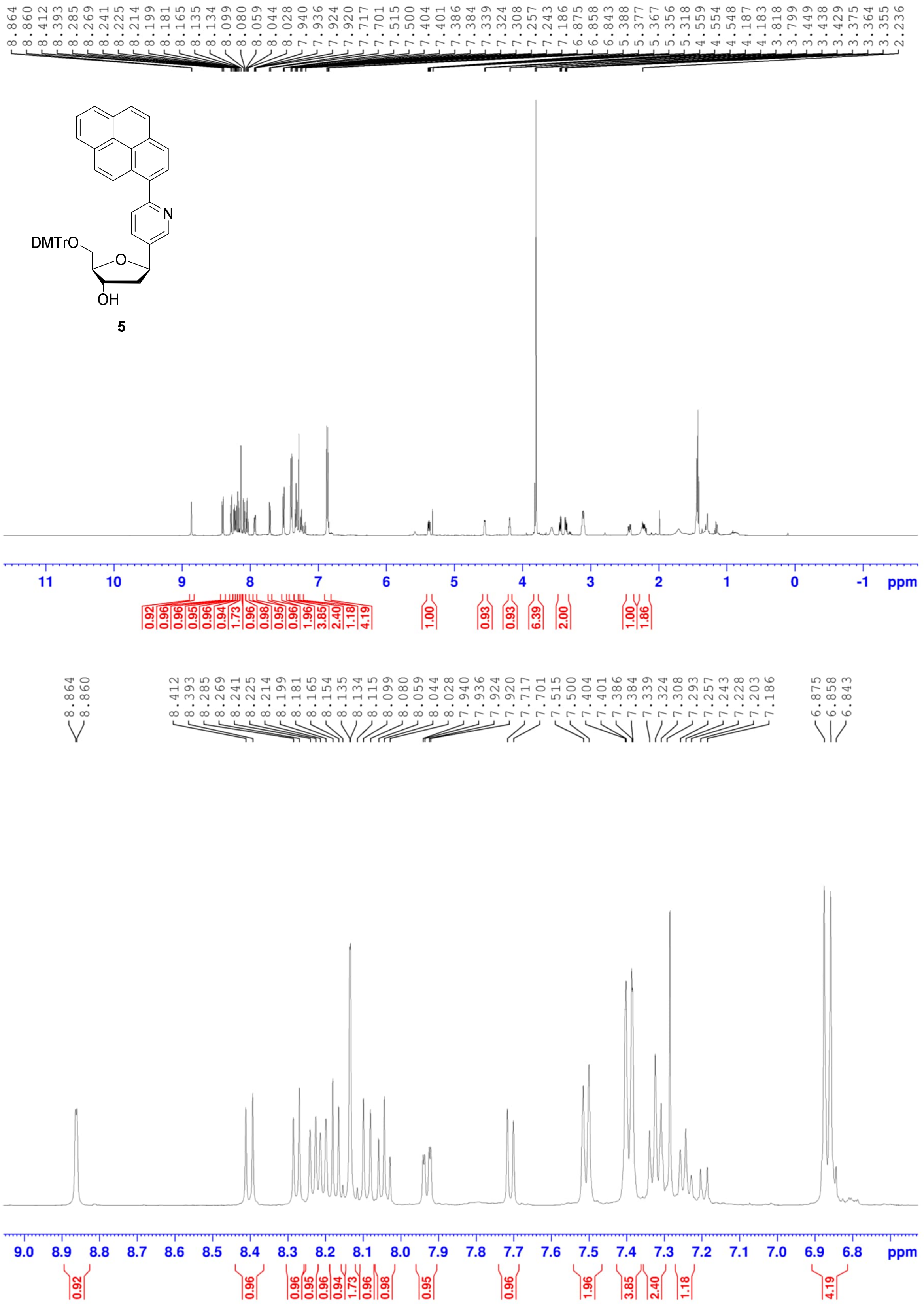

Figure S5. ^{1}H NMR spectrum of compound 5 (500 MHz, $CDCl_3$).

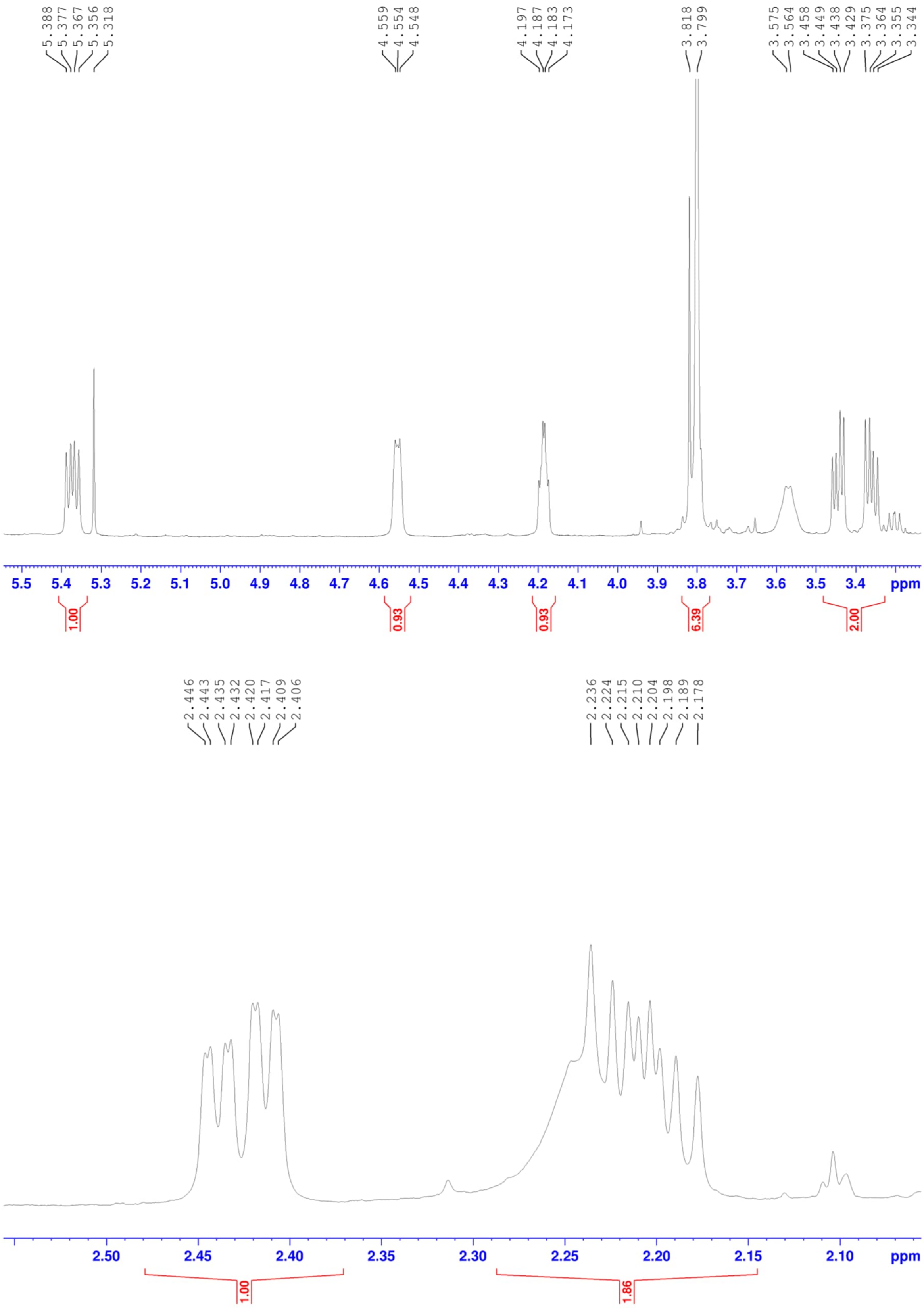

Figure S5 (continued). ^{1}H NMR spectrum of compound 5 (500 MHz, $CDCl_3$).

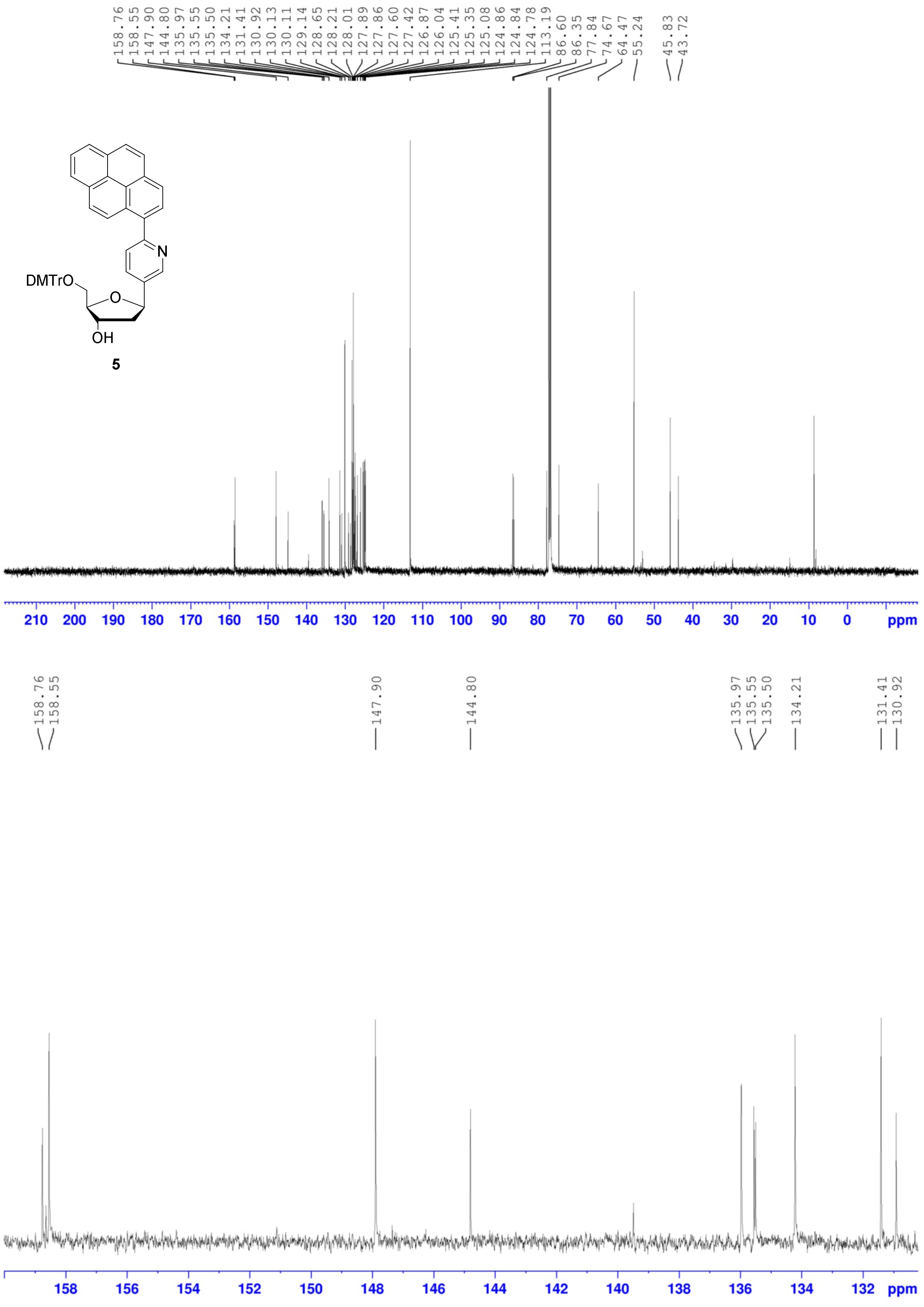

Figure S6. ^{13}C NMR spectrum of compound 5 (125 MHz, $CDCl_3$).

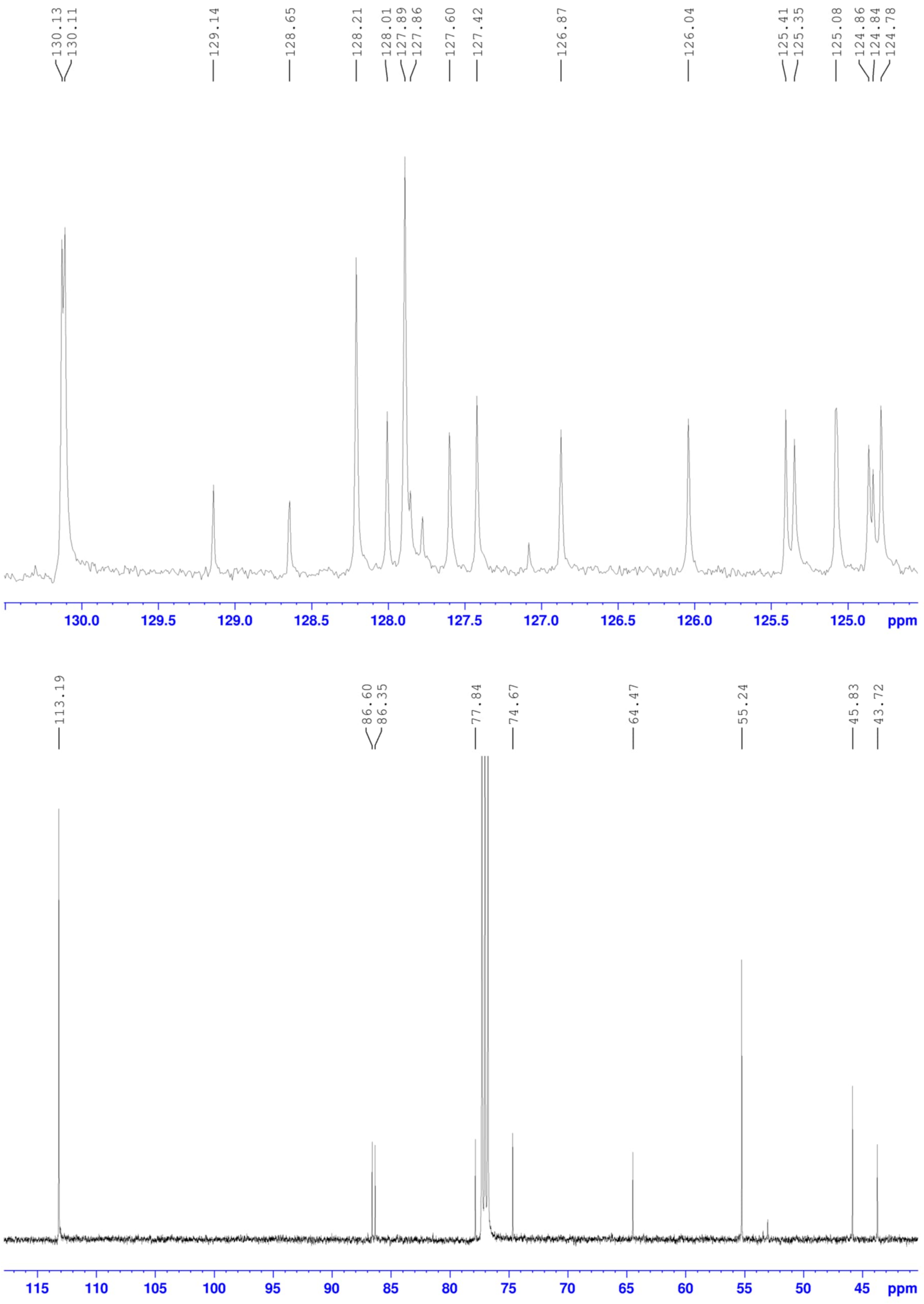

Figure S6 (continued). ^{13}C NMR spectrum of compound 5 (125 MHz, $CDCl_3$).

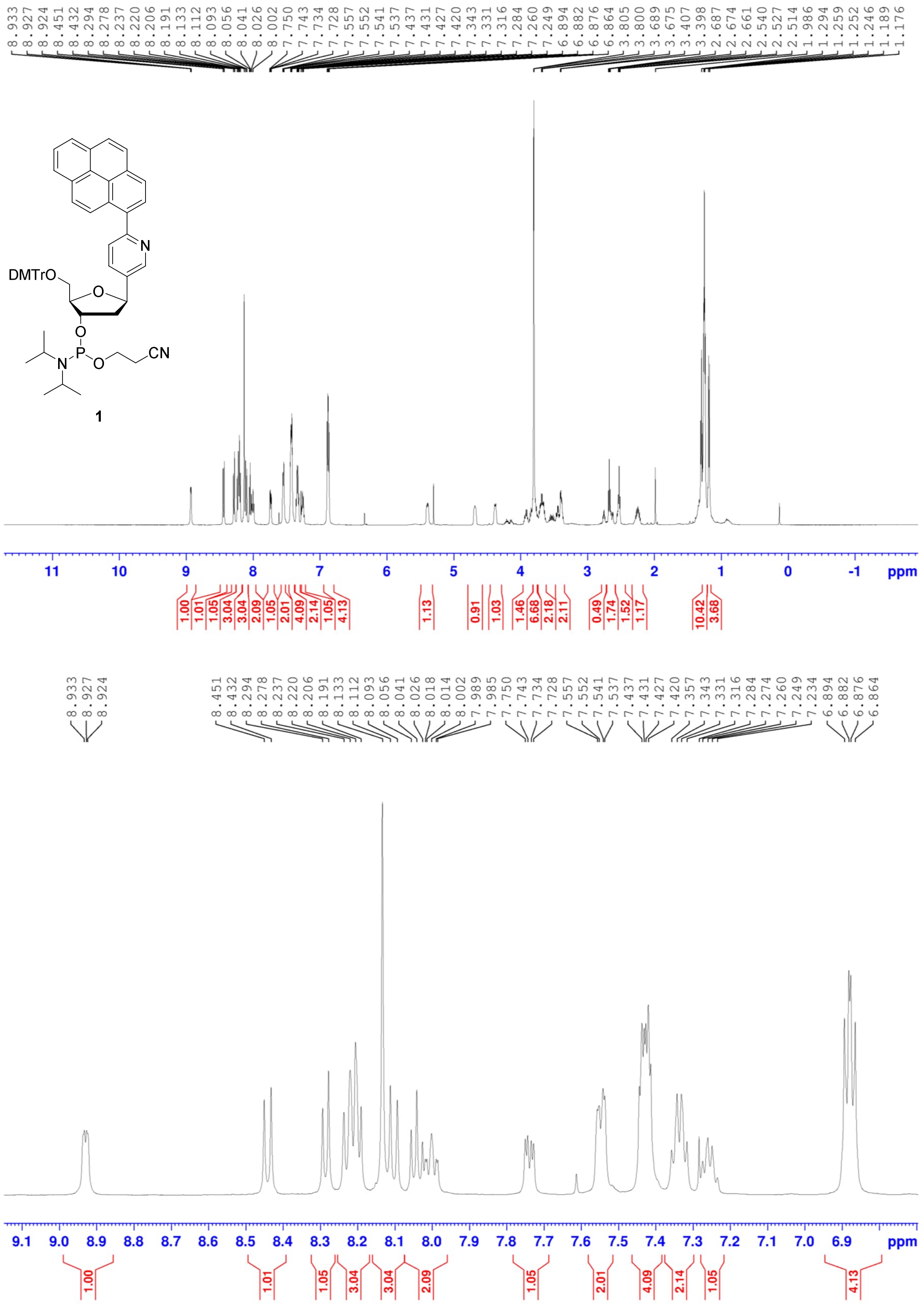

Figure S7. ^{1}H NMR spectrum of compound 1 (500 MHz, $CDCl_3$).

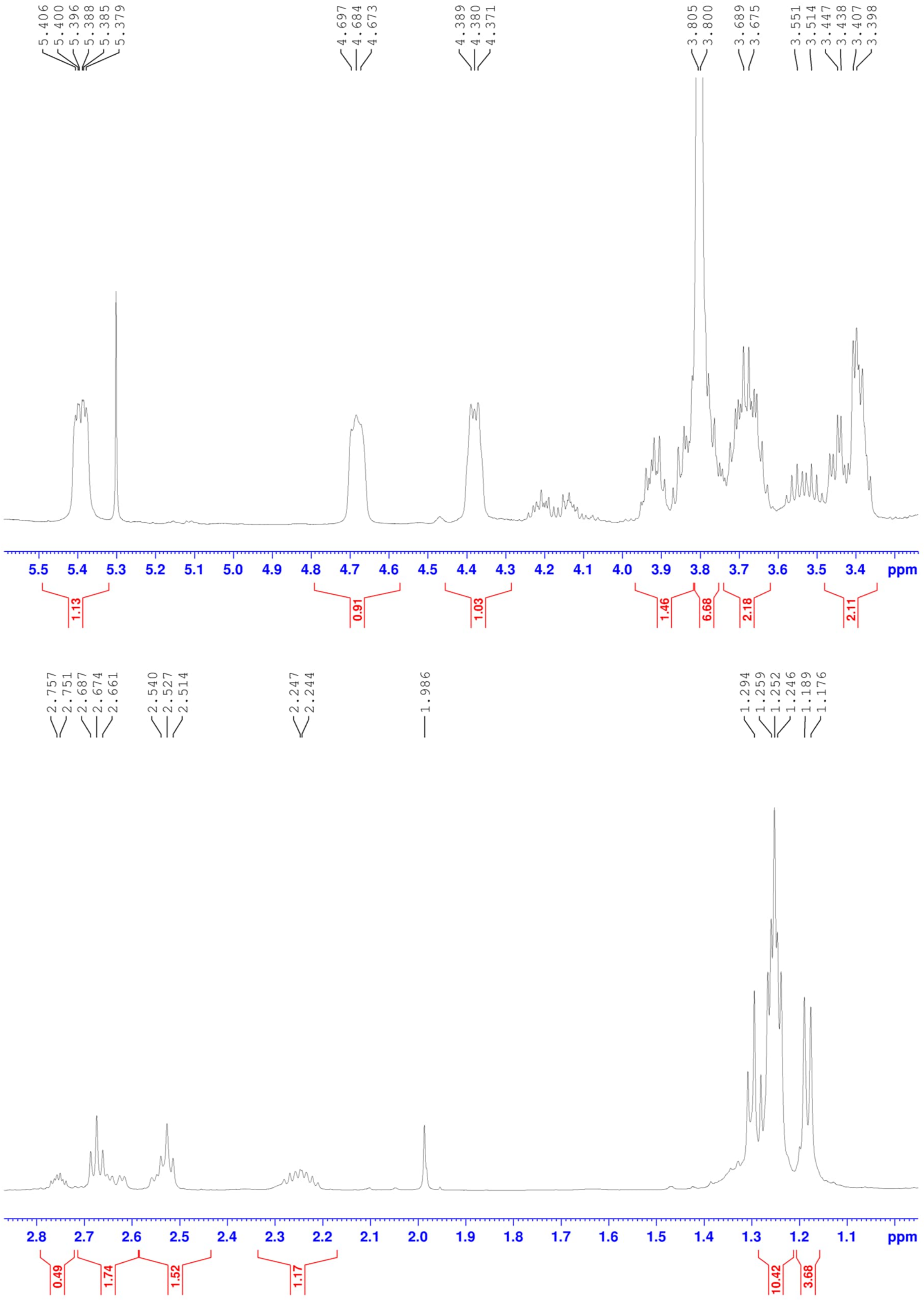

Figure S7 (continued). ^{1}H NMR spectrum of compound 1 (500 MHz, $CDCl_3$).

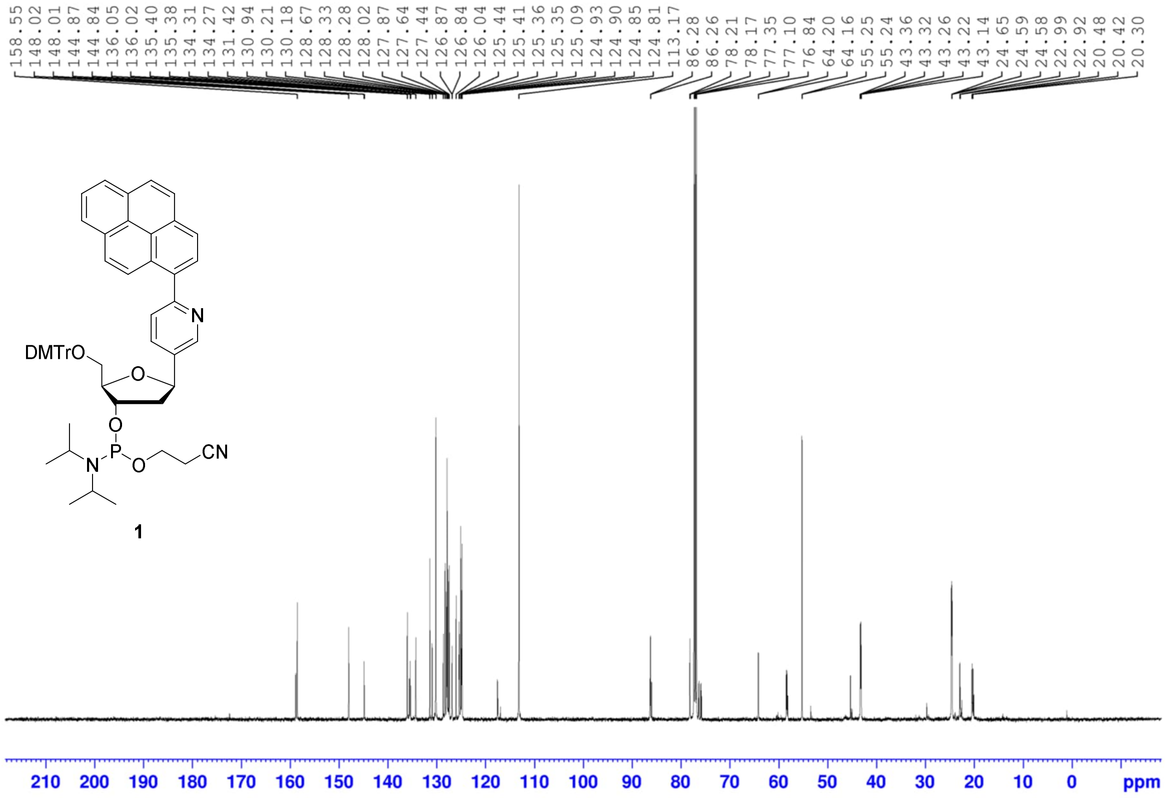

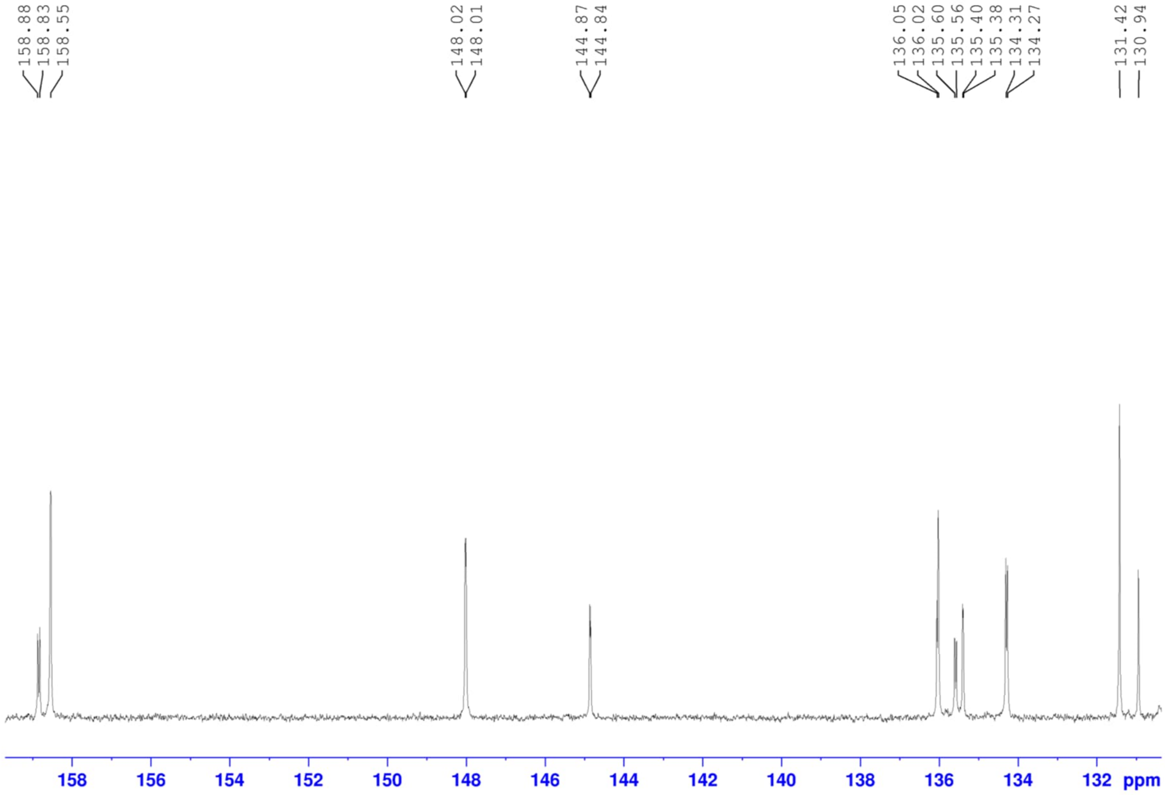

Figure S8. ^{13}C NMR spectrum of compound 1 (125 MHz, $CDCl_3$).

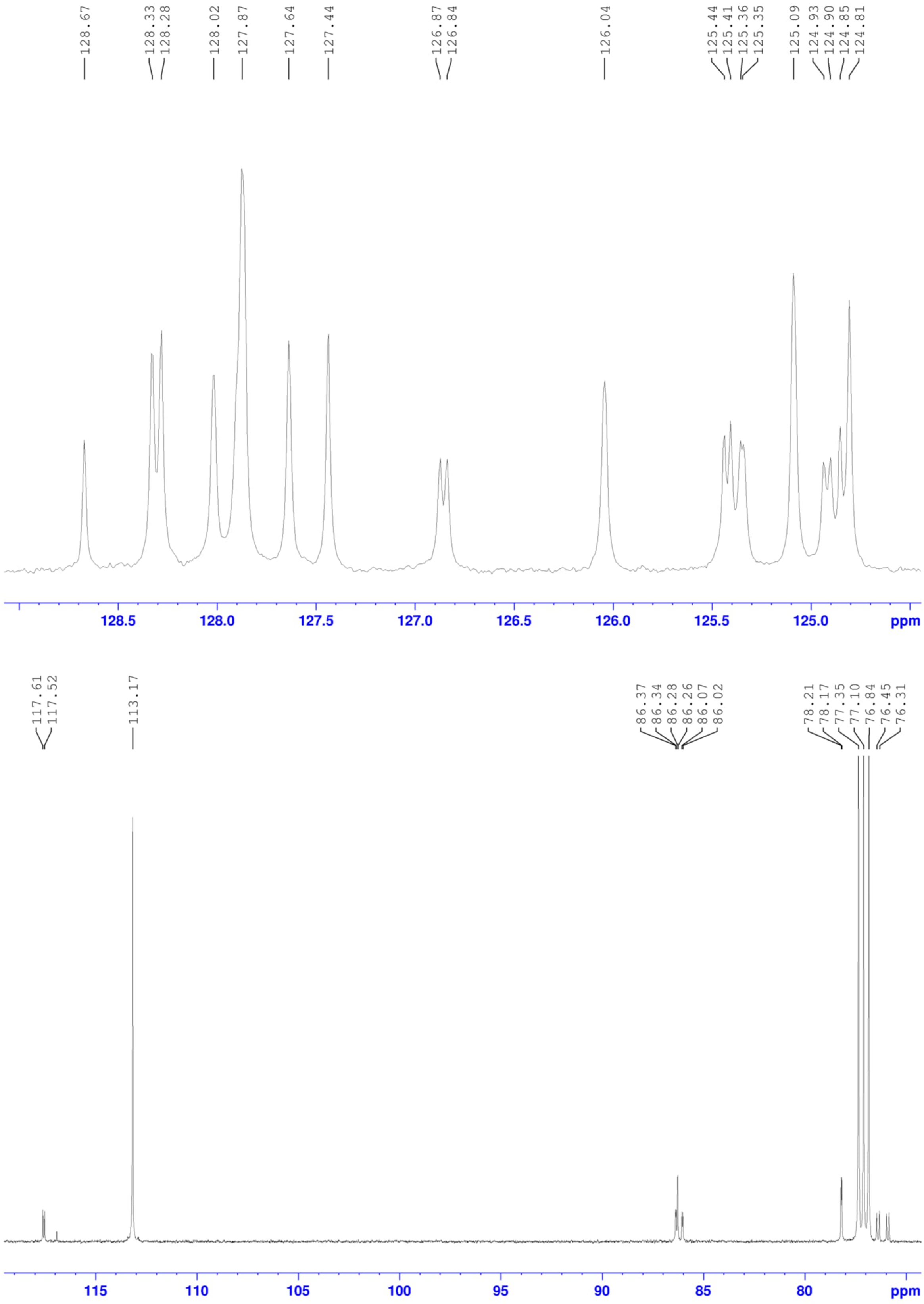

Figure S8 (continued). ^{13}C NMR spectrum of compound 1 (125 MHz, $CDCl_3$).

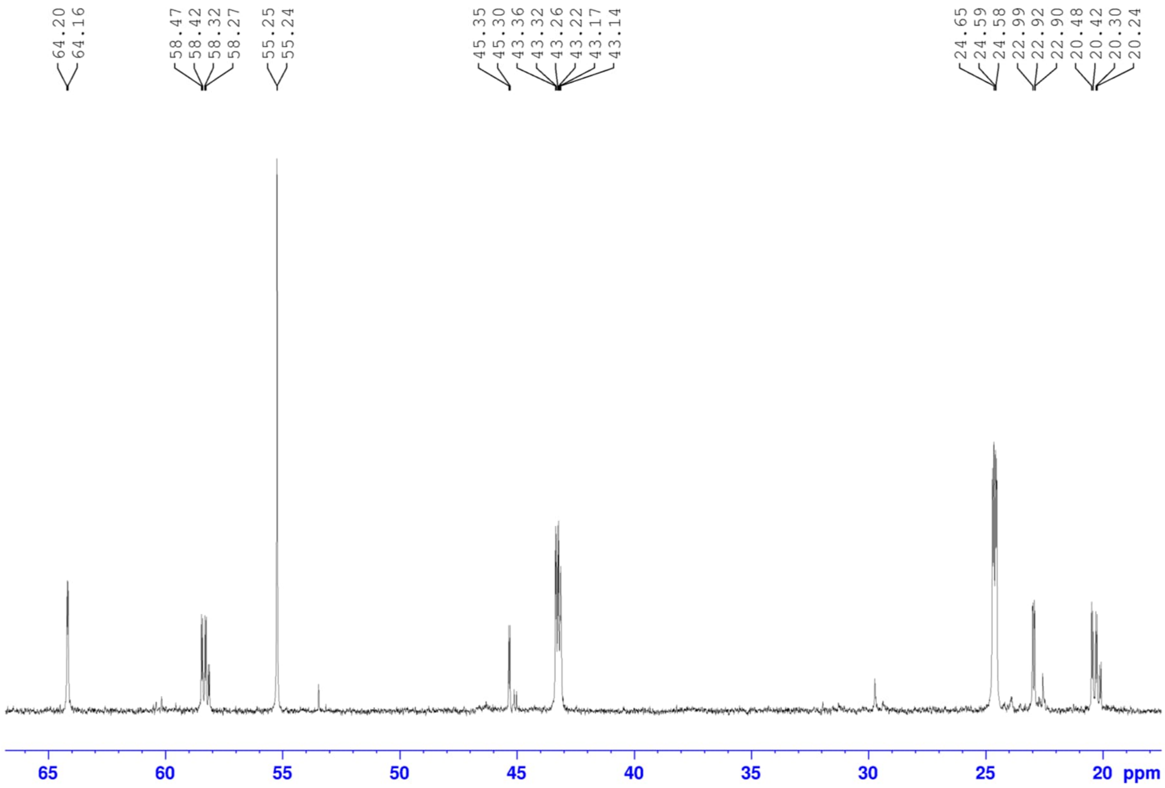

Figure S8 (continued). ^{13}C NMR spectrum of compound 1 (125 MHz, $CDCl_3$).

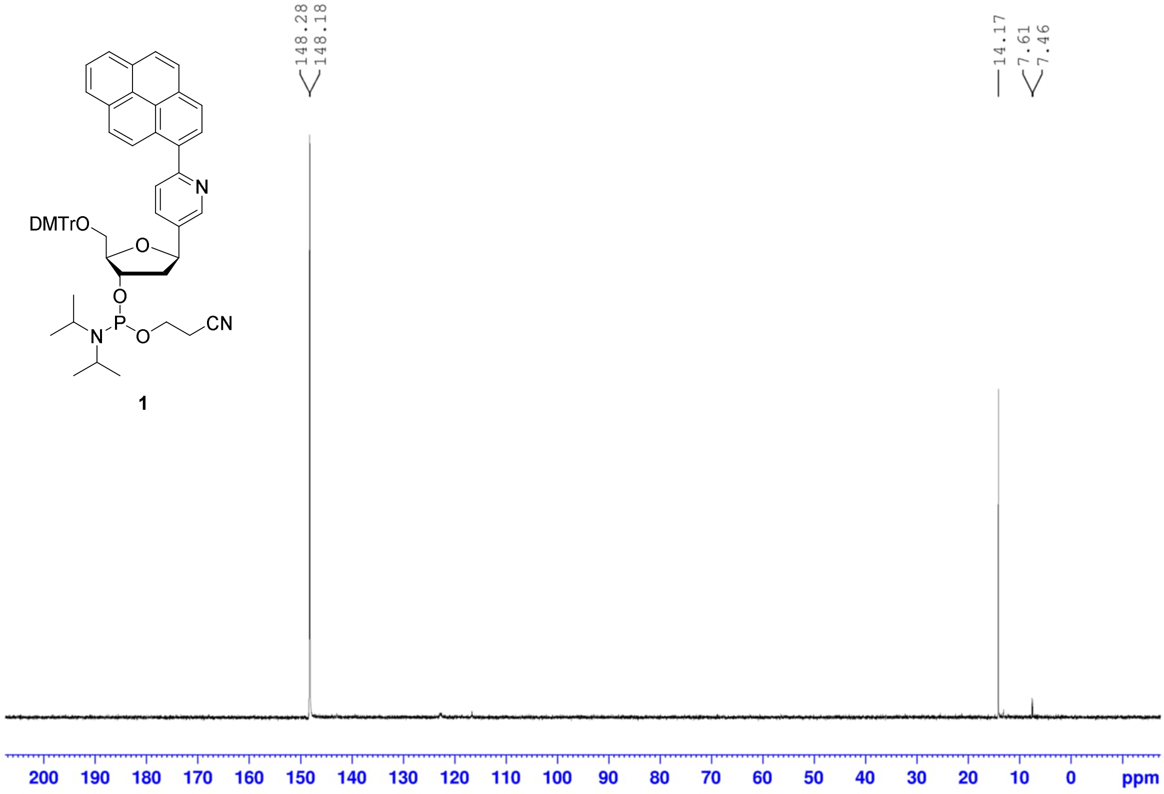

Figure S9. ^{31}P NMR spectrum of compound 1 (202 MHz, $CDCl_3$).

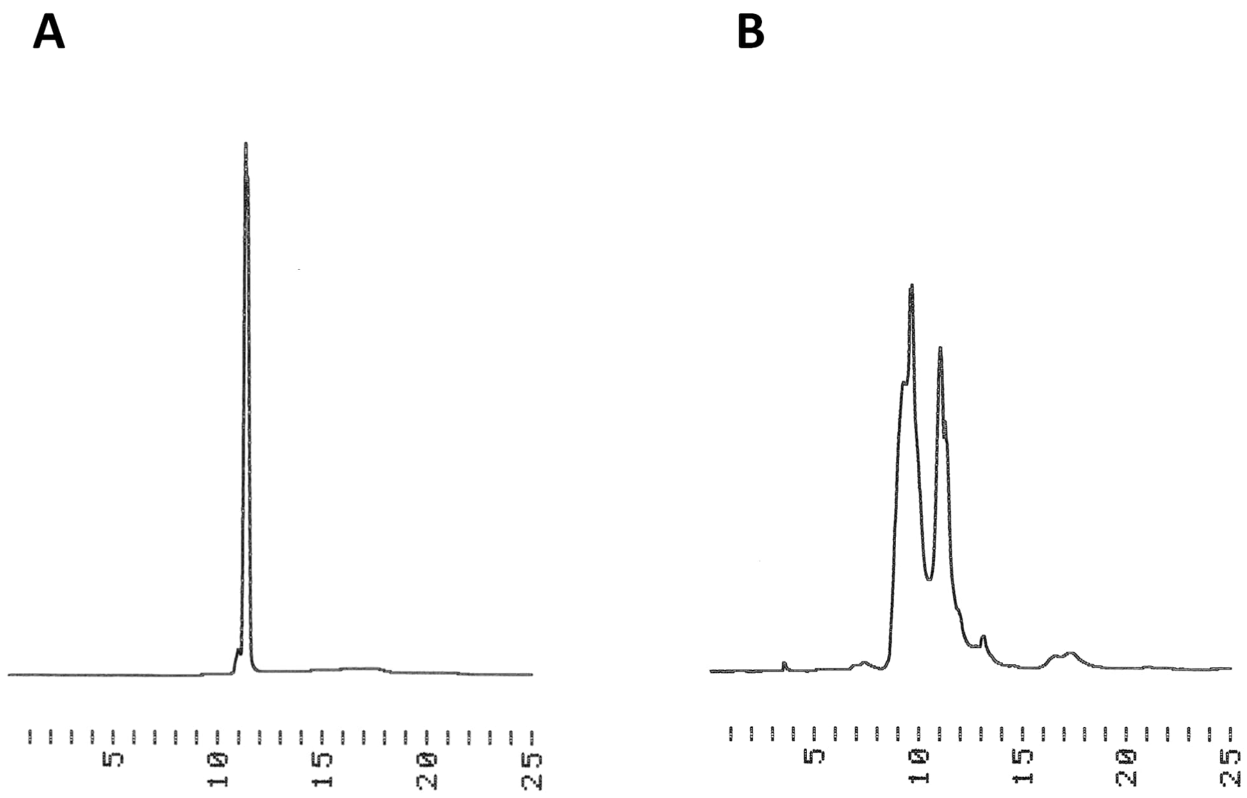

Figure S10. HPLC traces of oligonucleotides A) ON1pp and B) ON1pp-Pd; Hypersil ODS C18 column (250 × 4.6 mm, 5 µm); flow rate = 1 mL min^{-1}; linear gradient (5—40 % over 20 min) of MeCN in 50 mmol L^{-1} aqueous triethylammonium acetate; λ = 260 nm.

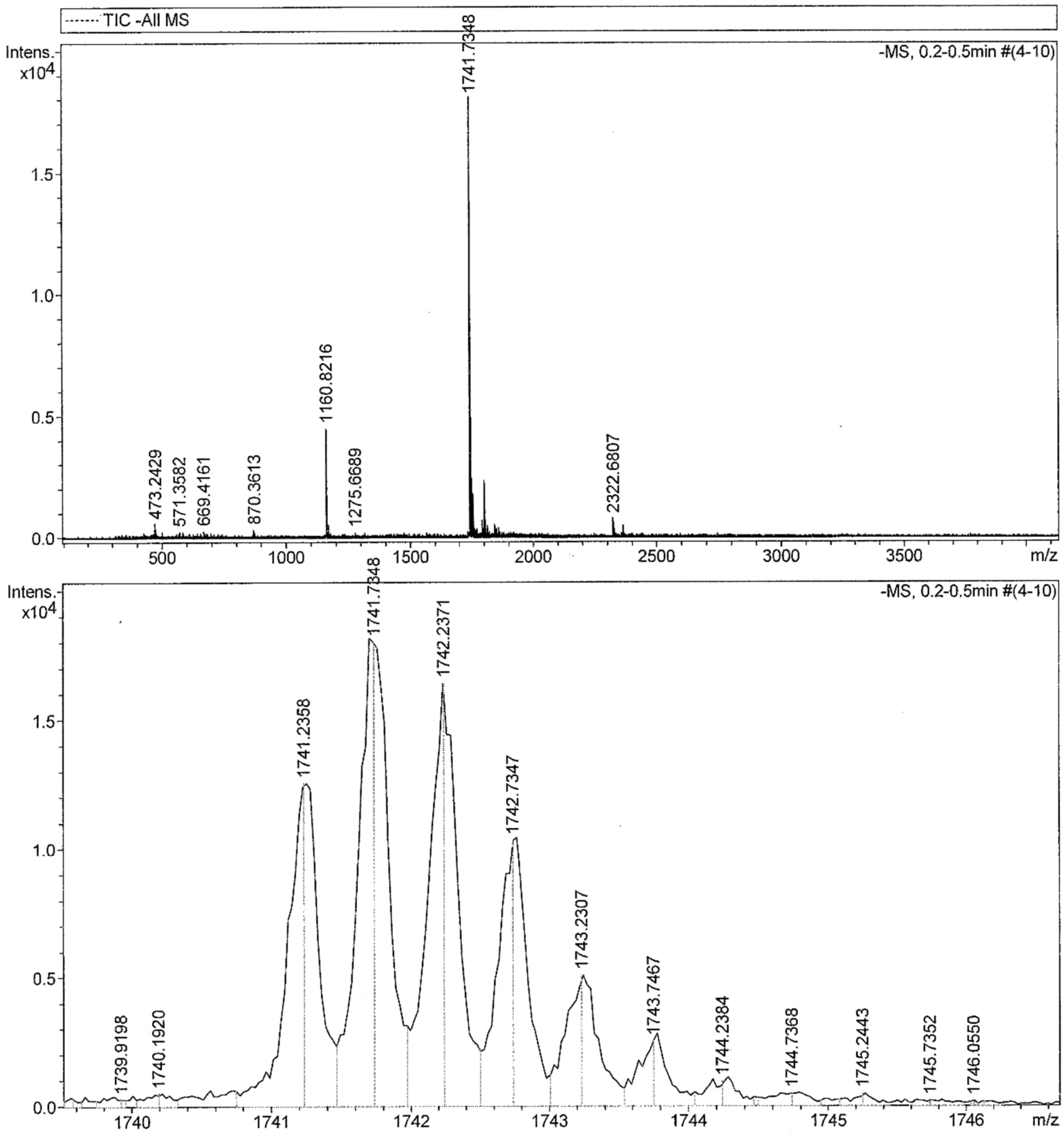

Figure S11. Mass spectrum of oligonucleotide ON1pp; *m/z* calculated for $C_{122}H_{140}N_{40}O_{63}P_{10}$: 1741.8201; found: 1741.7348 $[M-2H]^{2-}$.

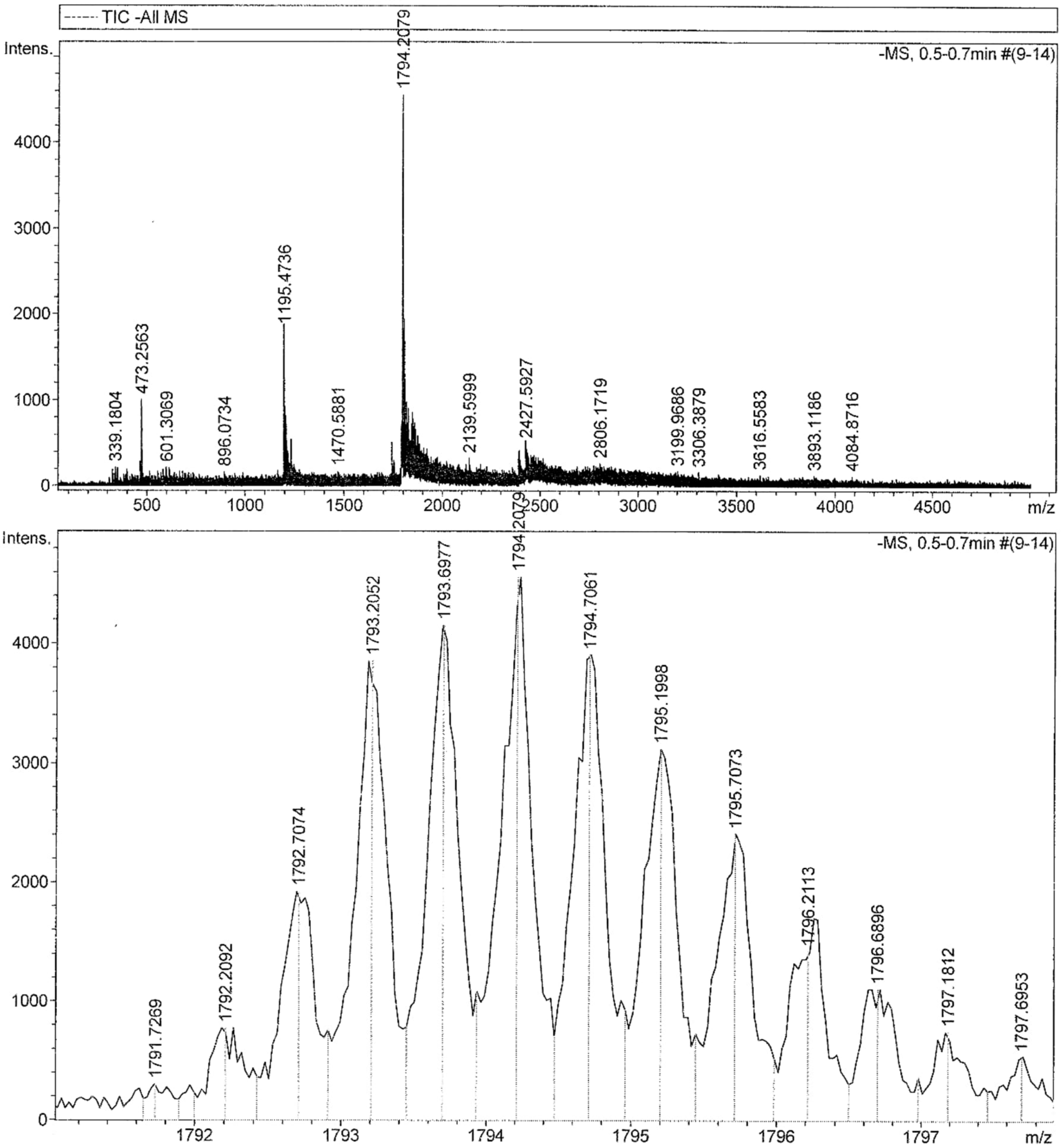

Figure S12. Mass spectrum of oligonucleotide ON1pp-Pd; *m/z* calculated for $C_{122}H_{138}N_{40}O_{63}P_{10}Pd$: 1793.7640; found: 1793.6977 $[M-3H]^{2-}$.

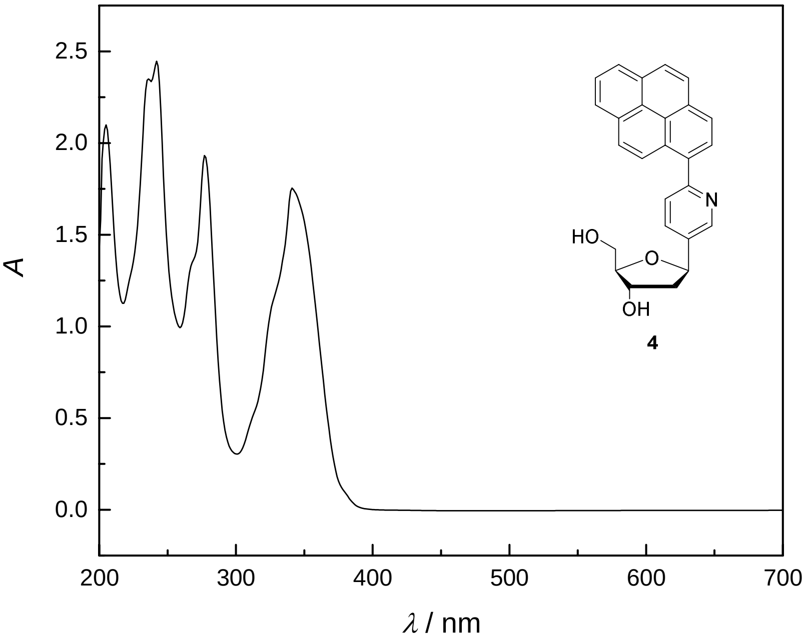

Figure S13. UV spectrum of compound 4 (0.30 mM in MeOH).

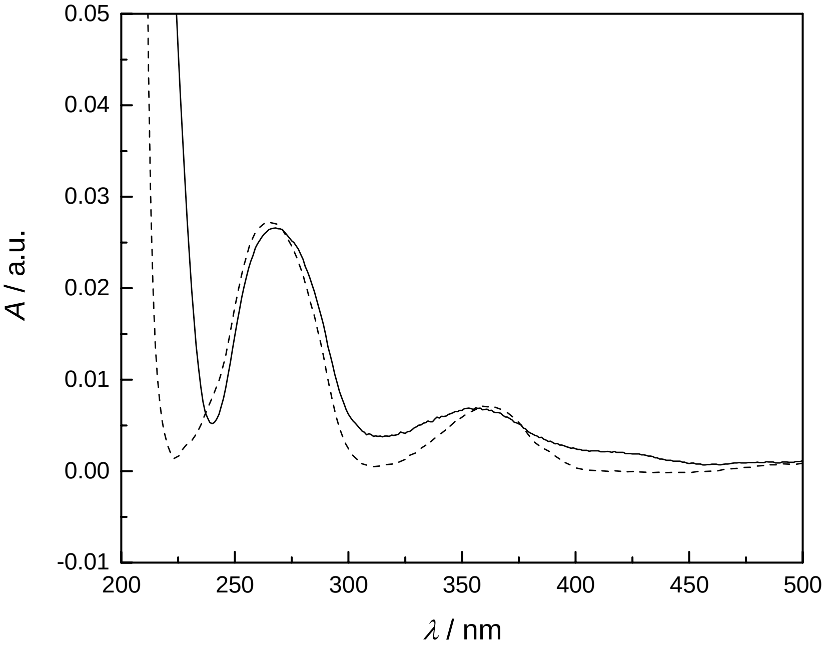

Figure S14. Normalized UV spectra of oligonucleotides ON1pp (dashed line) and ON1pp-Pd (solid line); pH = 7.4 (20 mM cacodylate buffer); *I* = 0.10 M (adjusted with $NaClO_4$).

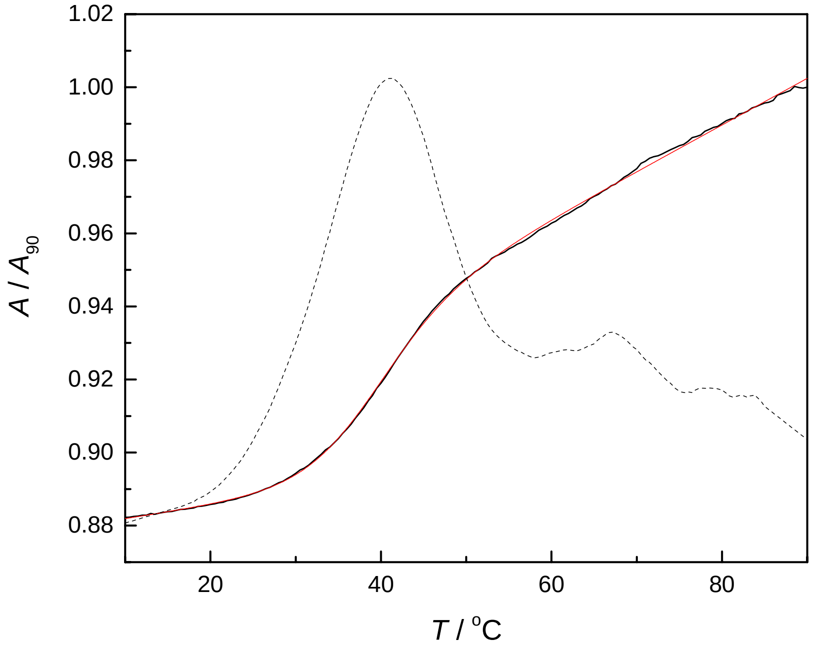

Figure S15. UV melting profile (solid black line), its first derivative (dashed black line) and fitting to Equation 1 (red line) for duplex ON1pp•ON2a; [oligonucleotides] = 1.0 µM; pH = 7.4 (20 mM cacodylate buffer); *I* = 0.10 M (adjusted with $NaClO_4$); λ = 260 nm.

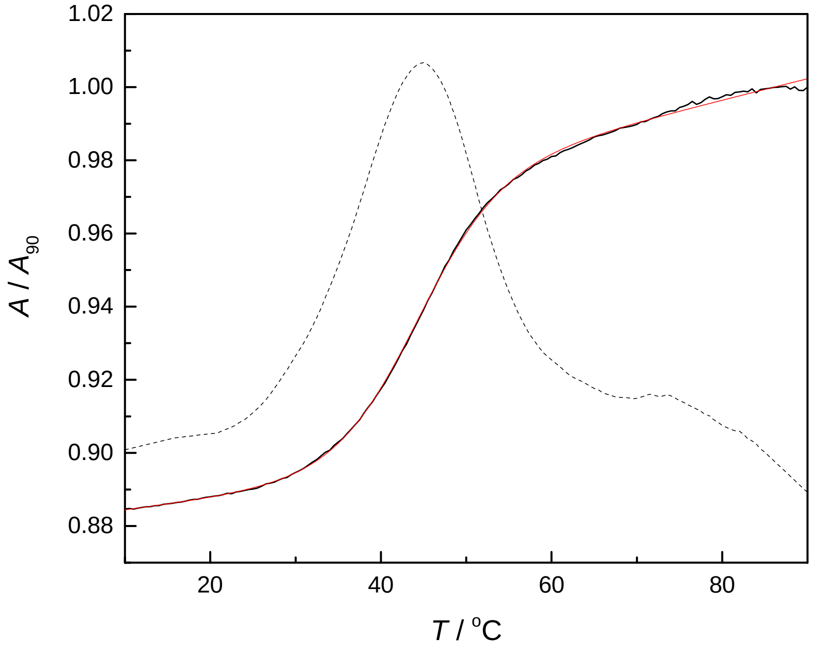

Figure S16. UV melting profile (solid black line), its first derivative (dashed black line) and fitting to Equation 1 (red line) for duplex ON1pp•ON2c; [oligonucleotides] = 1.0 µM; pH = 7.4 (20 mM cacodylate buffer); *I* = 0.10 M (adjusted with $NaClO_4$); λ = 260 nm.

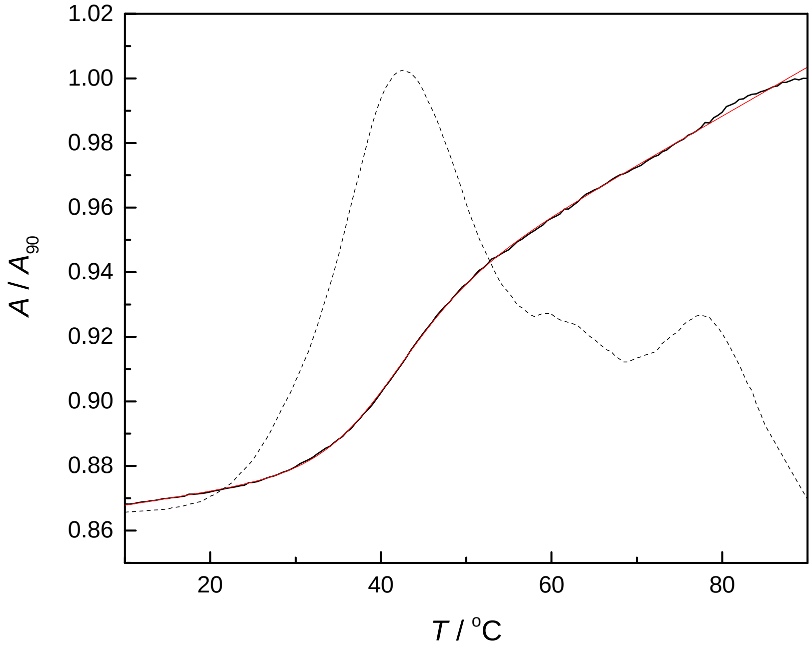

Figure S17. UV melting profile (solid black line), its first derivative (dashed black line) and fitting to Equation 1 (red line) for duplex ON1pp•ON2g; [oligonucleotides] = 1.0 µM; pH = 7.4 (20 mM cacodylate buffer); *I* = 0.10 M (adjusted with $NaClO_4$); λ = 260 nm.

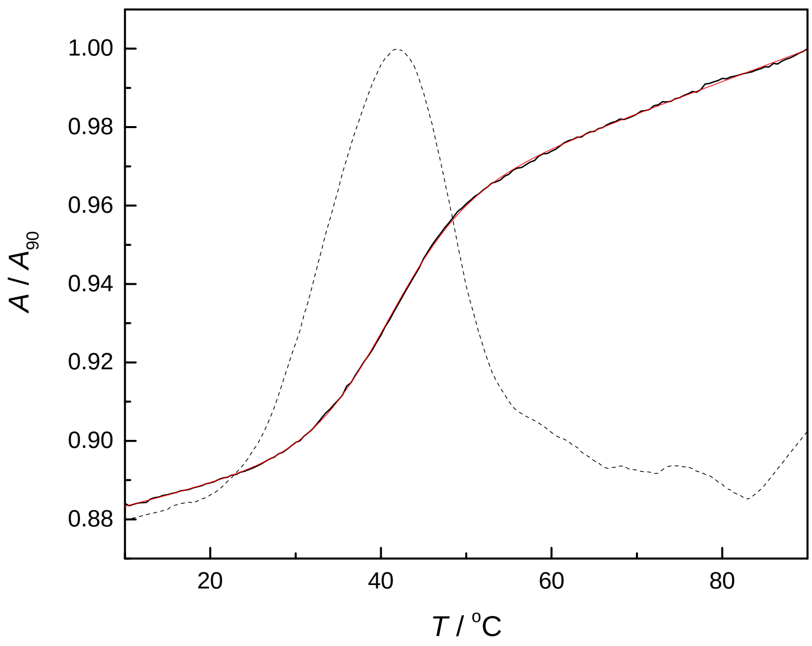

Figure S18. UV melting profile (solid black line), its first derivative (dashed black line) and fitting to Equation 1 (red line) for duplex ON1pp•ON2t; [oligonucleotides] = 1.0 µM; pH = 7.4 (20 mM cacodylate buffer); *I* = 0.10 M (adjusted with $NaClO_4$); λ = 260 nm.

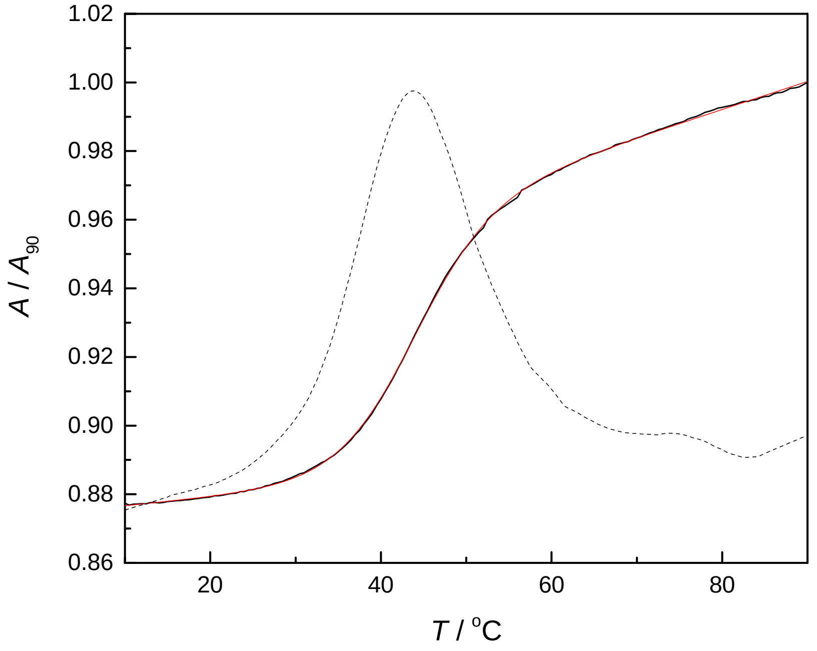

Figure S19. UV melting profile (solid black line), its first derivative (dashed black line) and fitting to Equation 1 (red line) for duplex ON1pp•ON2s; [oligonucleotides] = 1.0 µM; pH = 7.4 (20 mM cacodylate buffer); *I* = 0.10 M (adjusted with $NaClO_4$); λ = 260 nm.

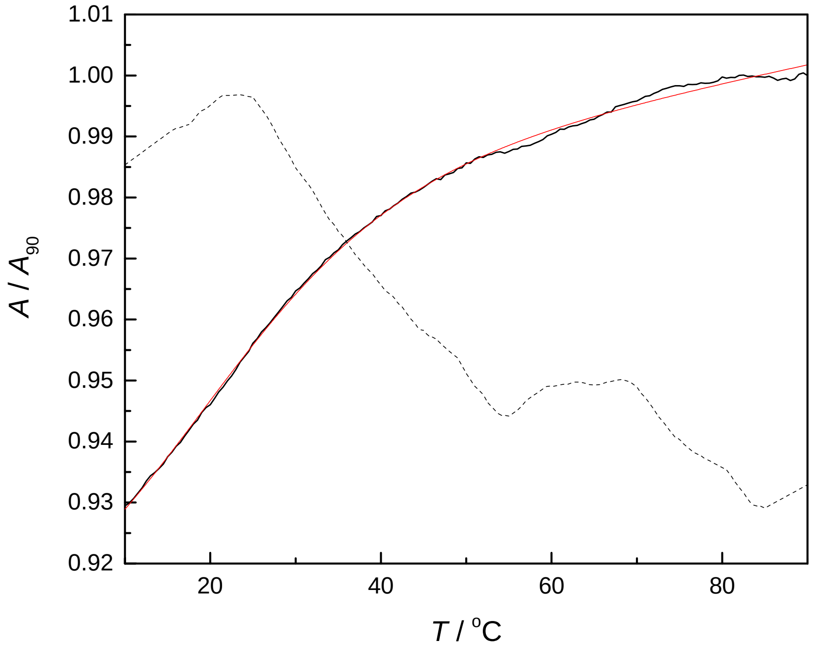

Figure S20. UV melting profile (solid black line), its first derivative (dashed black line) and fitting to Equation 1 (red line) for duplex ON1pp-Pd•ON2a; [oligonucleotides] = 1.0 µM; pH = 7.4 (20 mM cacodylate buffer); *I* = 0.10 M (adjusted with $NaClO_4$); λ = 260 nm.

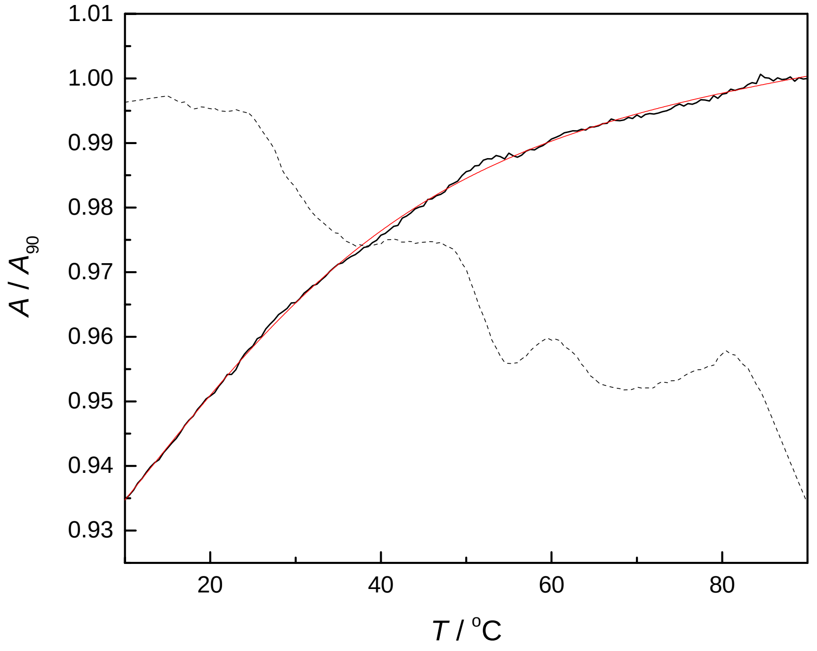

Figure S21. UV melting profile (solid black line), its first derivative (dashed black line) and fitting to Equation 1 (red line) for duplex ON1pp-Pd•ON2c; [oligonucleotides] = 1.0 µM; pH = 7.4 (20 mM cacodylate buffer); *I* = 0.10 M (adjusted with $NaClO_4$); λ = 260 nm.

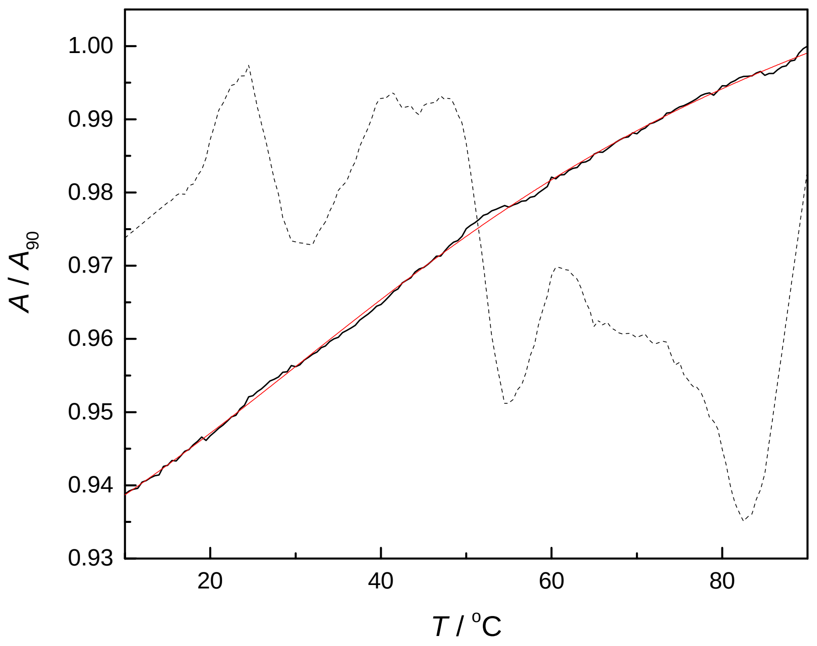

Figure S22. UV melting profile (solid black line), its first derivative (dashed black line) and fitting to Equation 1 (red line) for duplex ON1pp-Pd•ON2g; [oligonucleotides] = 1.0 µM; pH = 7.4 (20 mM cacodylate buffer); *I* = 0.10 M (adjusted with $NaClO_4$); λ = 260 nm.

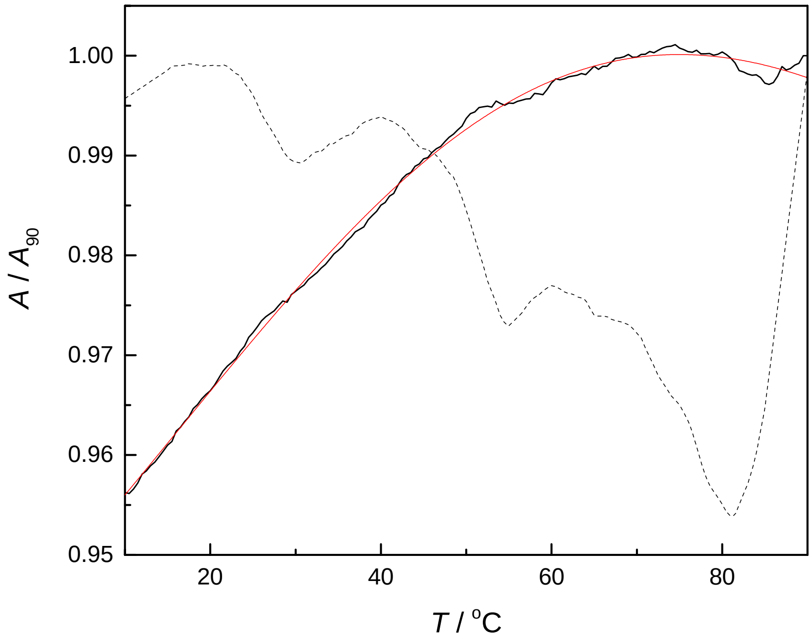

Figure S23. UV melting profile (solid black line), its first derivative (dashed black line) and fitting to Equation 1 (red line) for duplex ON1pp-Pd•ON2t; [oligonucleotides] = 1.0 µM; pH = 7.4 (20 mM cacodylate buffer); *I* = 0.10 M (adjusted with $NaClO_4$); λ = 260 nm.

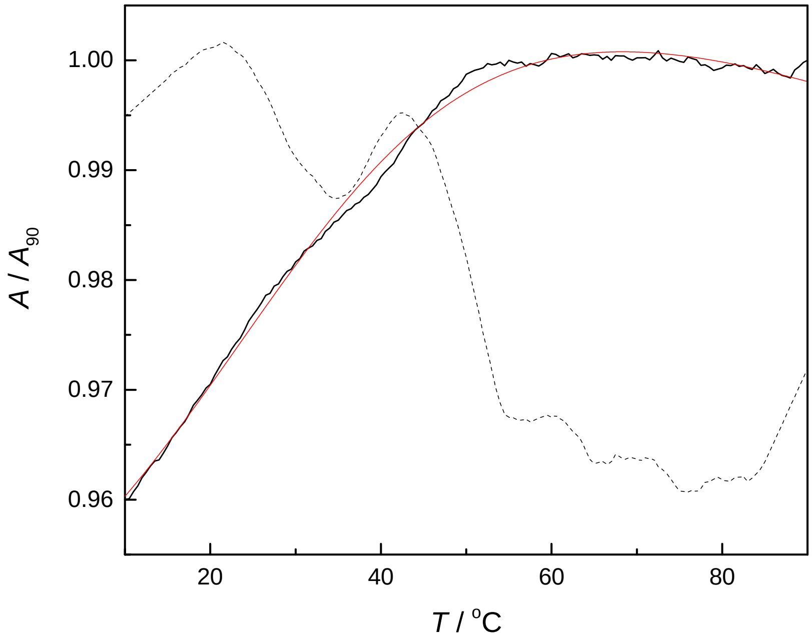

Figure S24. UV melting profile (solid black line), its first derivative (dashed black line) and fitting to Equation 1 (red line) for duplex ON1pp-Pd•ON2s; [oligonucleotides] = 1.0 µM; pH = 7.4 (20 mM cacodylate buffer); *I* = 0.10 M (adjusted with $NaClO_4$); λ = 260 nm.

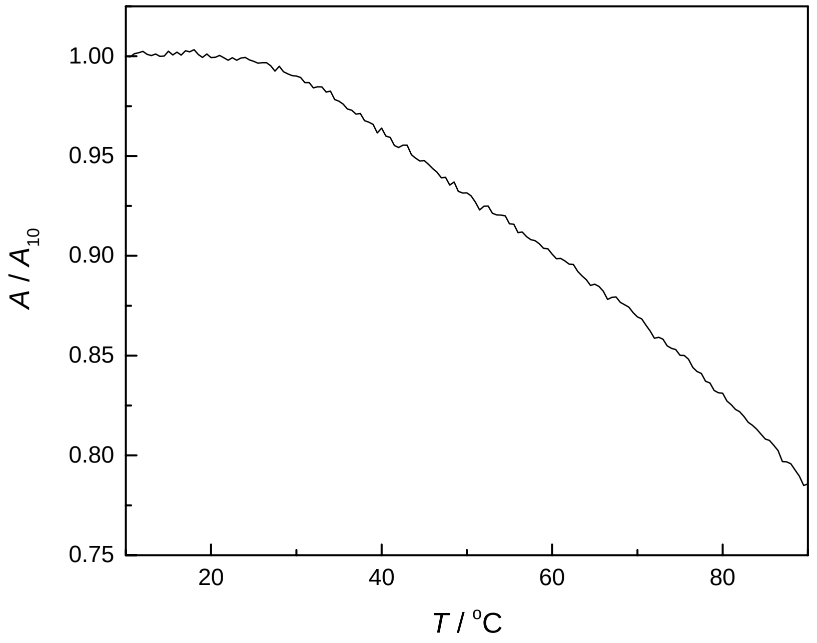

Figure S25. UV melting profile for duplex ON1pp•ON2a; [oligonucleotides] = 1.0 μM; pH = 7.4 (20 mM cacodylate buffer); *I* = 0.10 M (adjusted with $NaClO_4$); λ = 355 nm.

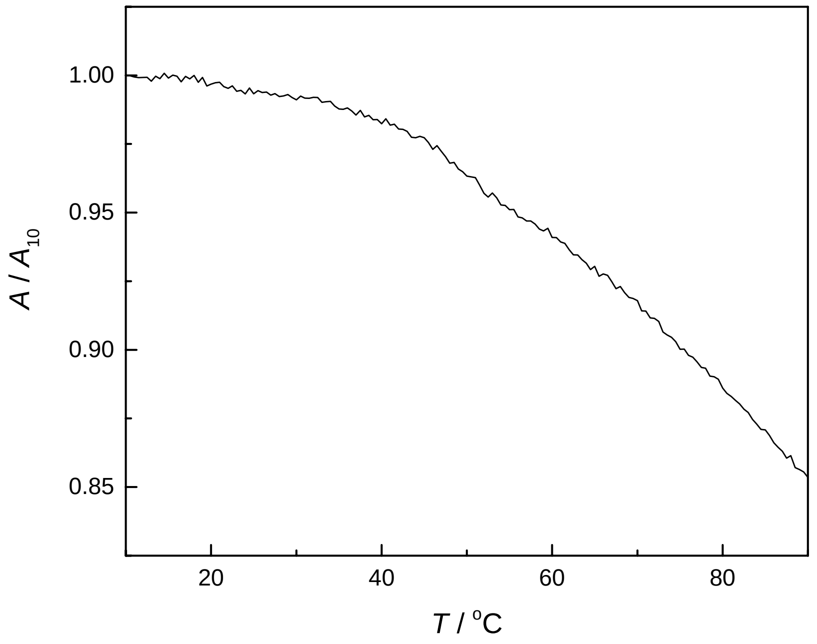

Figure S26. UV melting profile for duplex ON1pp•ON2c; [oligonucleotides] = 1.0 μM; pH = 7.4 (20 mM cacodylate buffer); *I* = 0.10 M (adjusted with $NaClO_4$); λ = 355 nm.

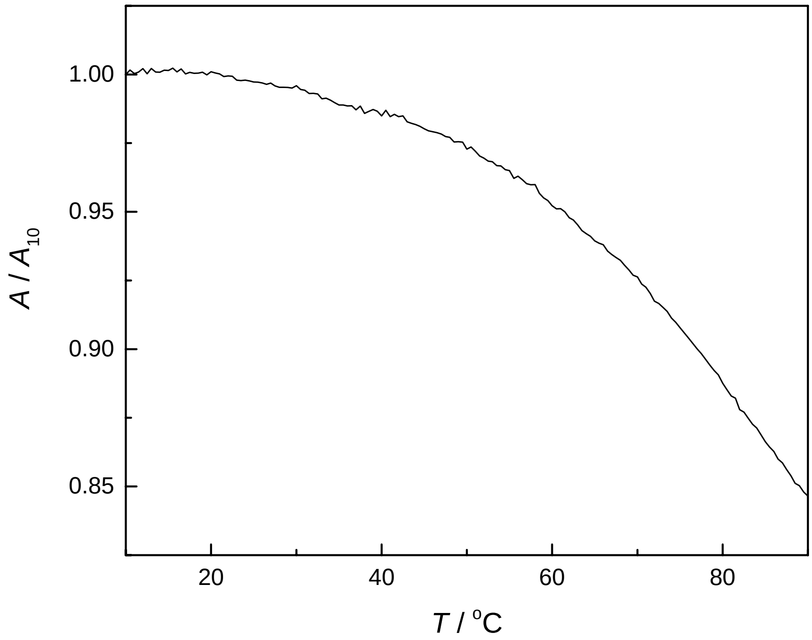

Figure S27. UV melting profile for duplex ON1pp•ON2g; [oligonucleotides] = 1.0 µM; pH = 7.4 (20 mM cacodylate buffer); I = 0.10 M (adjusted with $NaClO_4$); λ = 355 nm.

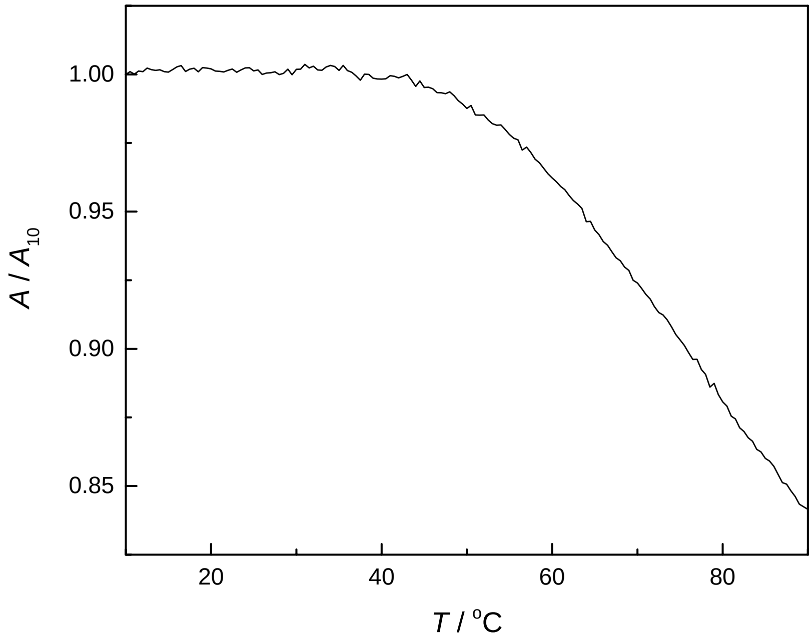

Figure S28. UV melting profile for duplex ON1pp•ON2t; [oligonucleotides] = 1.0 µM; pH = 7.4 (20 mM cacodylate buffer); I = 0.10 M (adjusted with $NaClO_4$); λ = 355 nm.

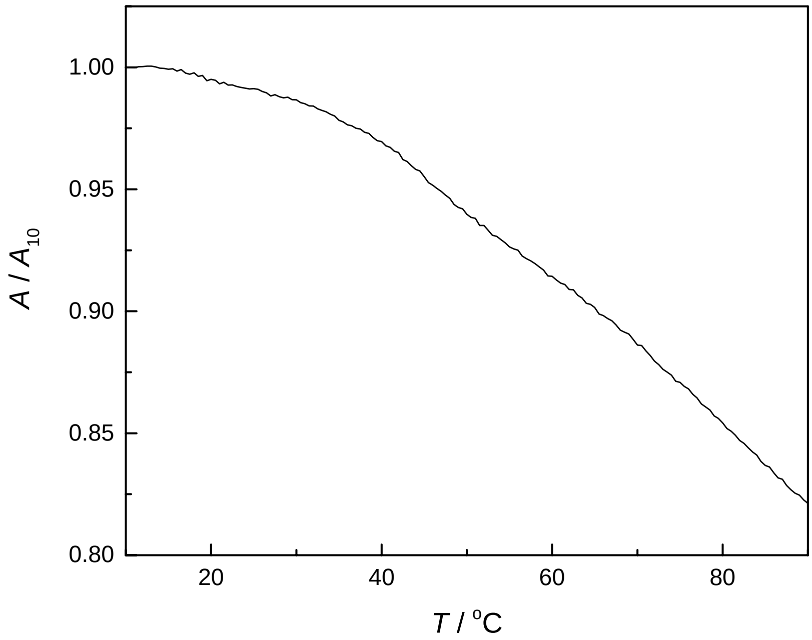

Figure S29. UV melting profile for duplex ON1pp•ON2s; [oligonucleotides] = 1.0 µM; pH = 7.4 (20 mM cacodylate buffer); *I* = 0.10 M (adjusted with $NaClO_4$); λ = 355 nm.

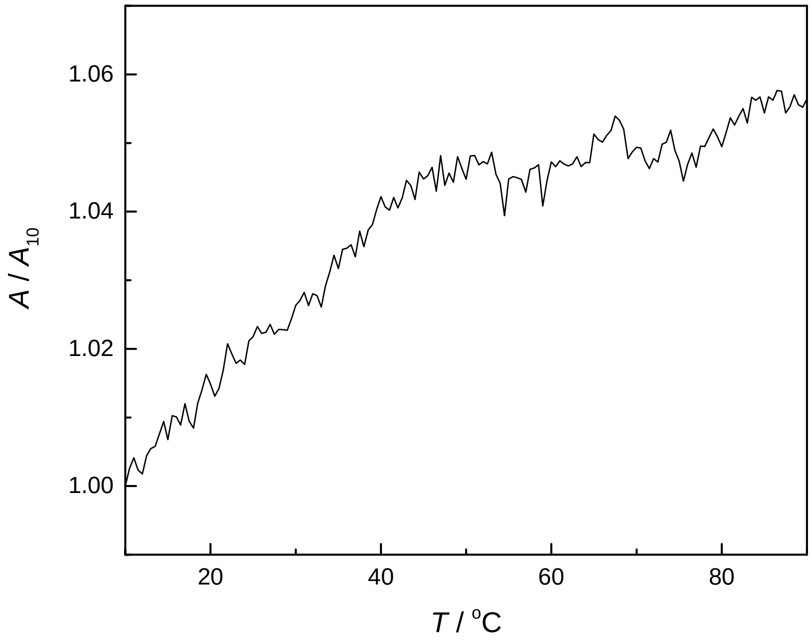

Figure S30. UV melting profile for duplex ON1pp-Pd•ON2a; [oligonucleotides] = 1.0 µM; pH = 7.4 (20 mM cacodylate buffer); *I* = 0.10 M (adjusted with $NaClO_4$); λ = 355 nm.

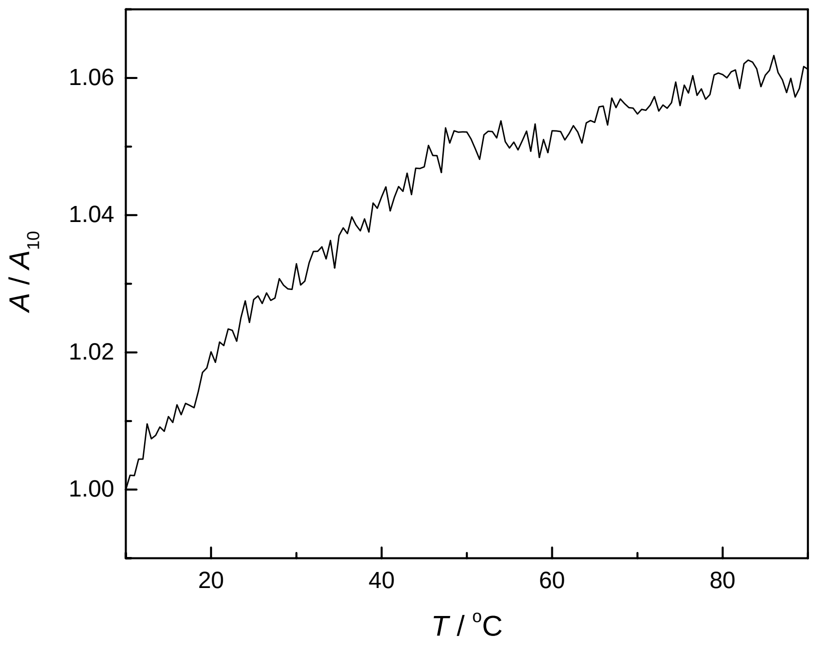

Figure S31. UV melting profile for duplex ON1pp-Pd•ON2c; [oligonucleotides] = 1.0 µM; pH = 7.4 (20 mM cacodylate buffer); I = 0.10 M (adjusted with $NaClO_4$); λ = 355 nm.

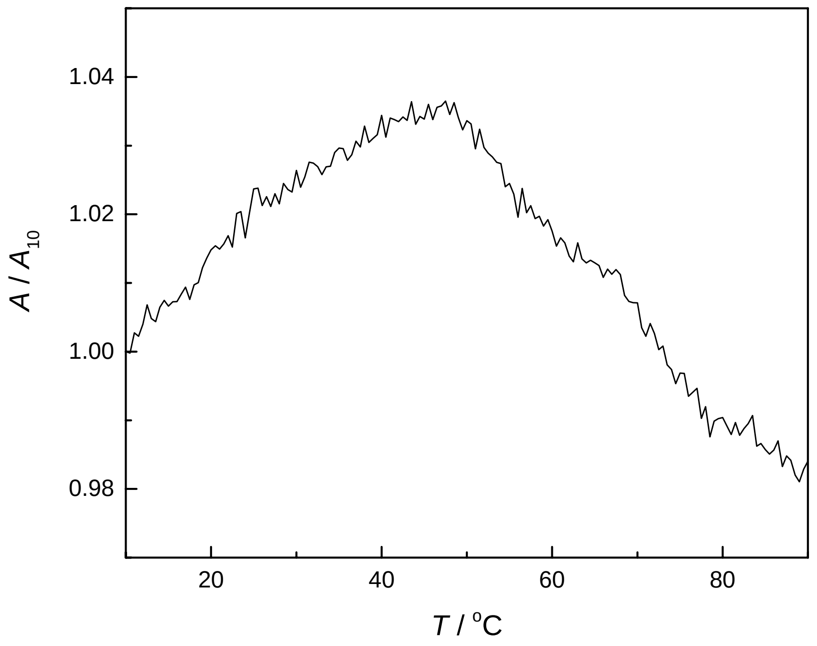

Figure S32. UV melting profile for duplex ON1pp-Pd•ON2g; [oligonucleotides] = 1.0 µM; pH = 7.4 (20 mM cacodylate buffer); I = 0.10 M (adjusted with $NaClO_4$); λ = 355 nm.

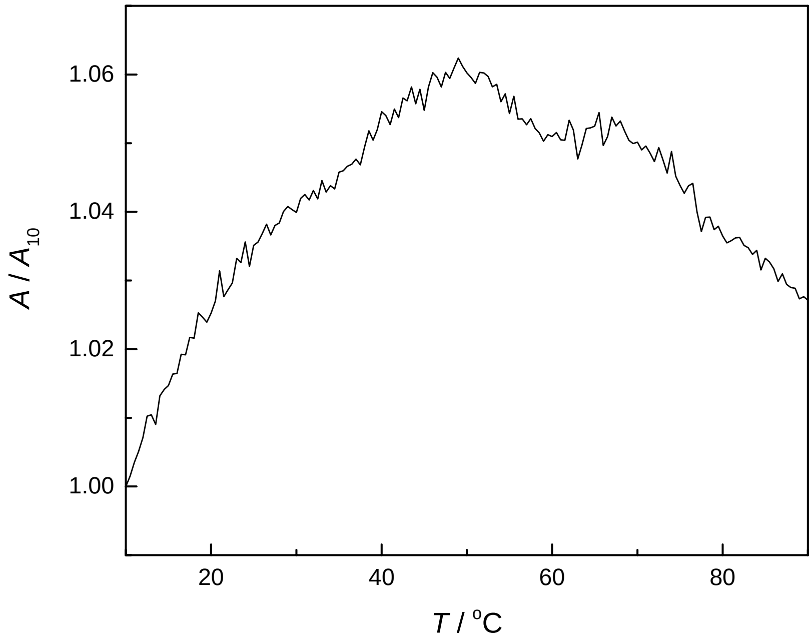

Figure S33. UV melting profile for duplex ON1pp-Pd•ON2t; [oligonucleotides] = 1.0 µM; pH = 7.4 (20 mM cacodylate buffer); *I* = 0.10 M (adjusted with $NaClO_4$); λ = 355 nm.

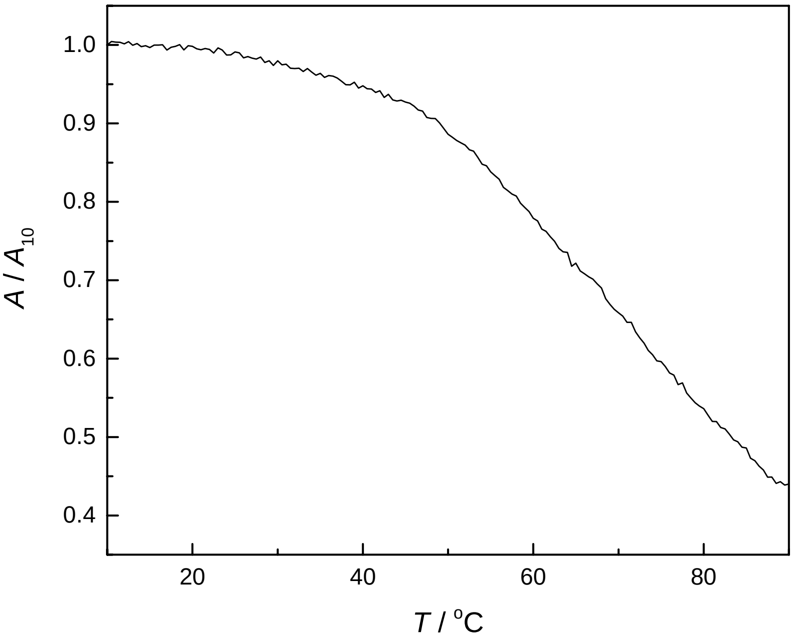

Figure S34. UV melting profile for duplex ON1pp-Pd•ON2s; [oligonucleotides] = 1.0 µM; pH = 7.4 (20 mM cacodylate buffer); *I* = 0.10 M (adjusted with $NaClO_4$); λ = 355 nm.

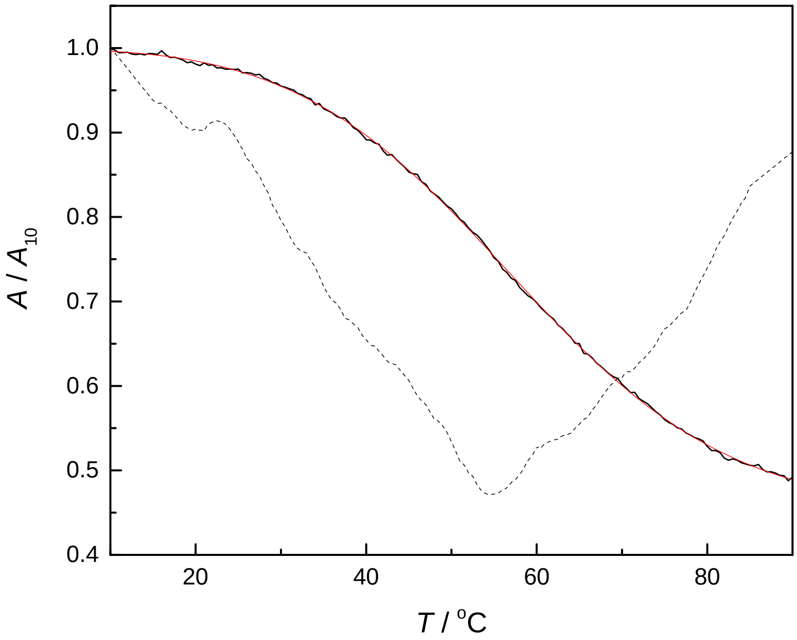

Figure S35. UV melting profile (solid black line), its first derivative (dashed black line) and fitting to Equation 1 (red line) for duplex ON1pp-Pd•ON2a; [oligonucleotides] = 1.0 µM; pH = 7.4 (20 mM cacodylate buffer); *I* = 0.10 M (adjusted with $NaClO_4$); λ = 425 nm.

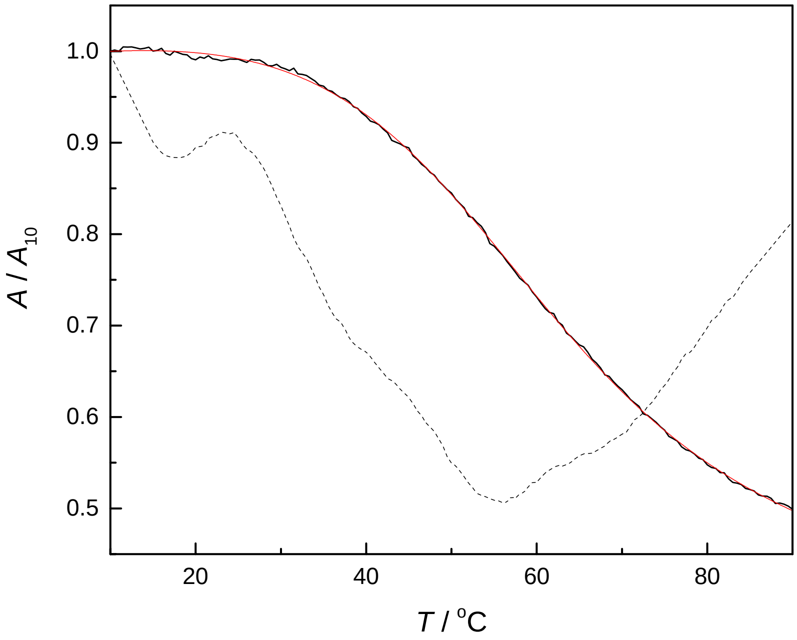

Figure S36. UV melting profile (solid black line), its first derivative (dashed black line) and fitting to Equation 1 (red line) for duplex ON1pp-Pd•ON2c; [oligonucleotides] = 1.0 µM; pH = 7.4 (20 mM cacodylate buffer); *I* = 0.10 M (adjusted with $NaClO_4$); λ = 425 nm.

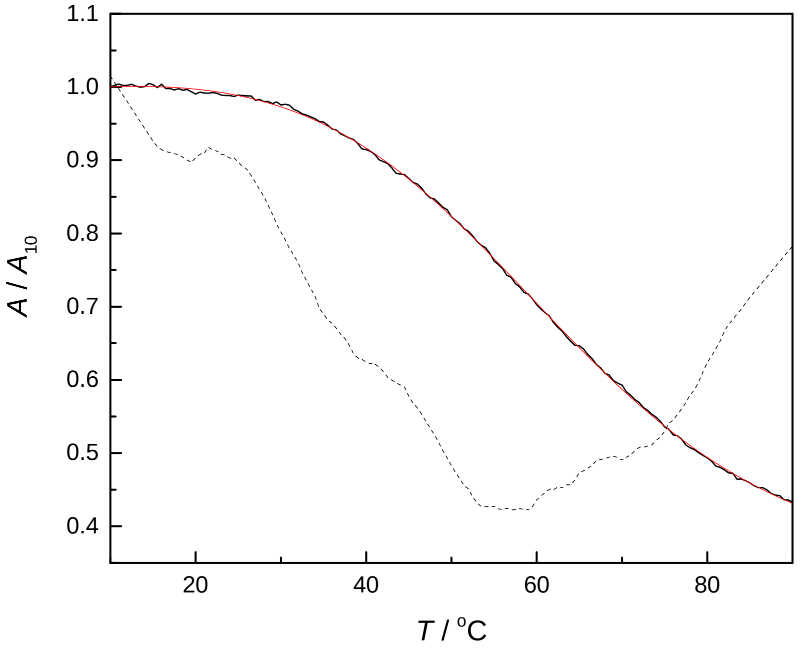

Figure S37. UV melting profile (solid black line), its first derivative (dashed black line) and fitting to Equation 1 (red line) for duplex ON1pp-Pd•ON2g; [oligonucleotides] = 1.0 µM; pH = 7.4 (20 mM cacodylate buffer); *I* = 0.10 M (adjusted with $NaClO_4$); λ = 425 nm.

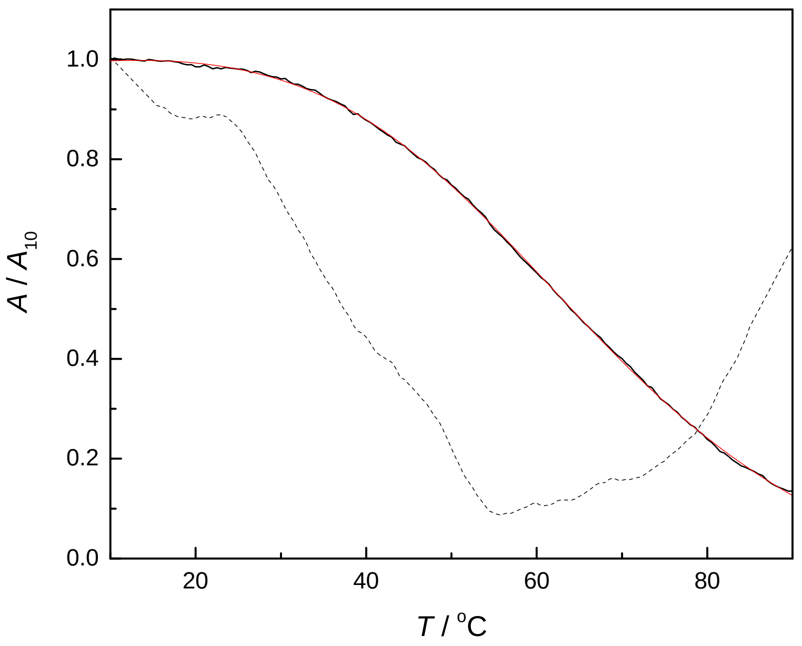

Figure S38. UV melting profile (solid black line), its first derivative (dashed black line) and fitting to Equation 1 (red line) for duplex ON1pp-Pd•ON2t; [oligonucleotides] = 1.0 µM; pH = 7.4 (20 mM cacodylate buffer); *I* = 0.10 M (adjusted with $NaClO_4$); λ = 425 nm.

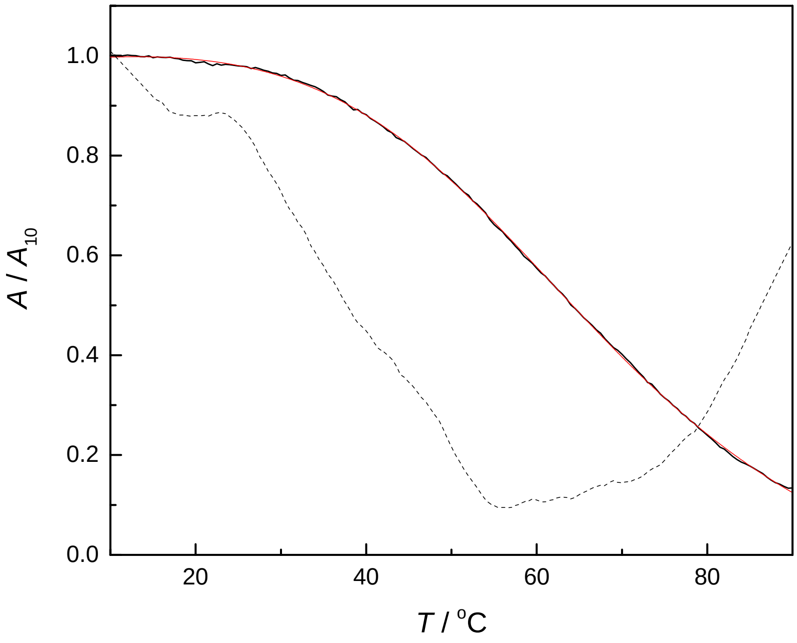

Figure S39. UV melting profile (solid black line), its first derivative (dashed black line) and fitting to Equation 1 (red line) for duplex ON1pp-Pd•ON2s; [oligonucleotides] = 1.0 µM; pH = 7.4 (20 mM cacodylate buffer); *I* = 0.10 M (adjusted with $NaClO_4$); λ = 425 nm.

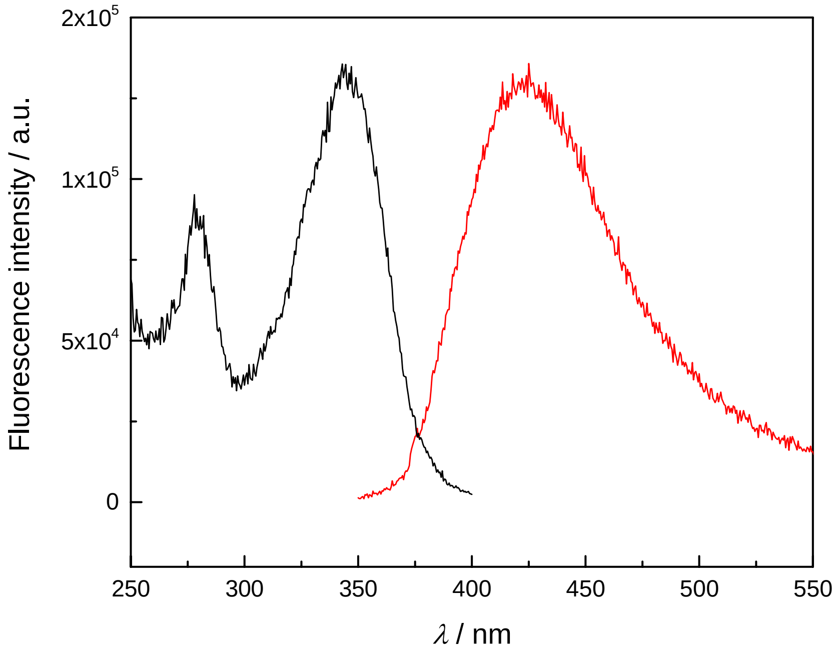

Figure S40. Excitation (black line) and emission (red line) spectra of compound 4 (1.0 mM in MeOH). For the excitation scan, λ_{em} was fixed at 425 nm and for the emission scan, λ_{ex} was fixed at 345 nm.

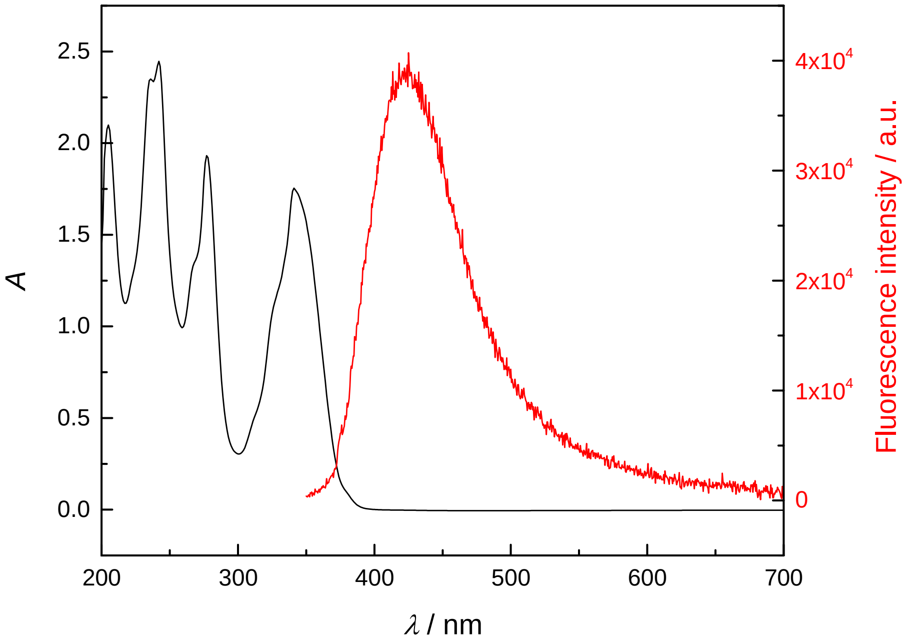

Figure S41. Absorption (black line) and emission (red line) spectra of compound 4 (0.3 mM in MeOH). For the emission scan, λ_{ex} was fixed at 345 nm.

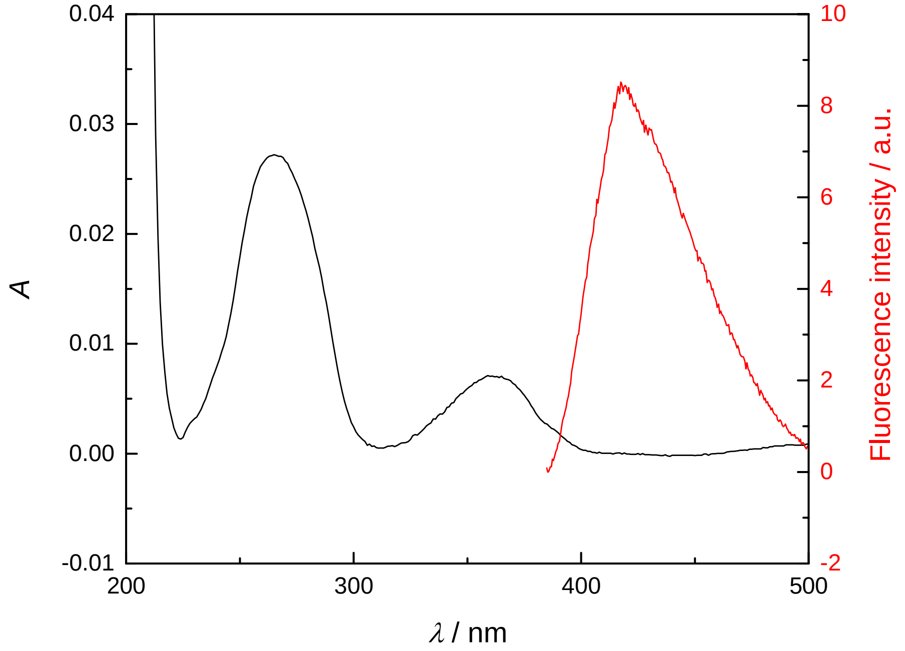

Figure S42. Absorption (black line) and emission (red line) spectra of oligonucleotide ON1pp; [ON1pp] = 300 nM (absorption) / 50 nm (emission); pH = 7.4 (20 mM cacodylate buffer); *I* = 0.10 M (adjusted with $NaClO_4$); λ_{ex} = 365 nm.

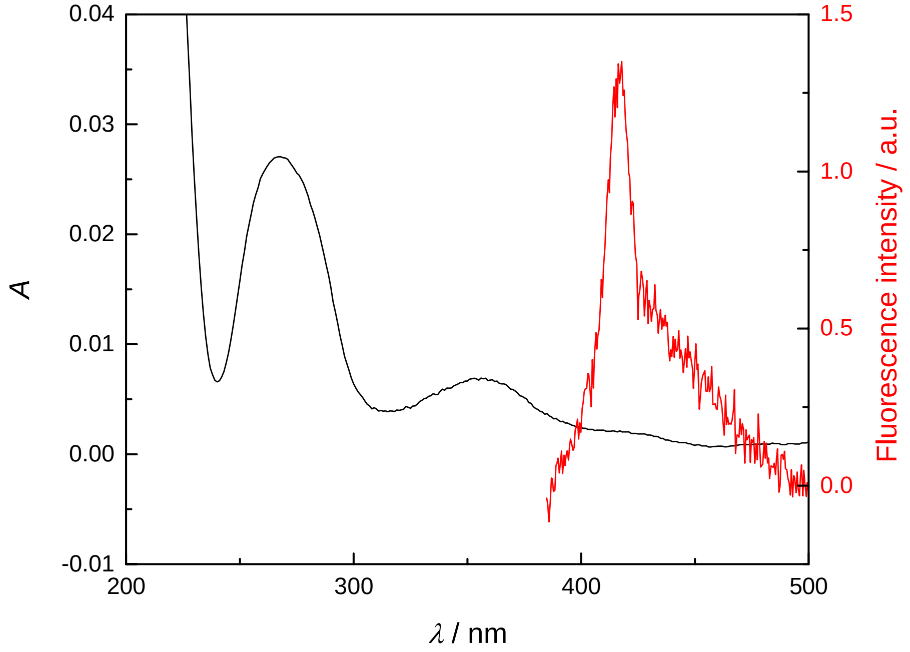

Figure S43. Absorption (black line) and emission (red line) spectra of oligonucleotide ON1pp-Pd; [ON1pp-Pd] = 300 nM (absorption) / 50 nm (emission); pH = 7.4 (20 mM cacodylate buffer); *I* = 0.10 M (adjusted with $NaClO_4$); λ_{ex} = 365 nm.

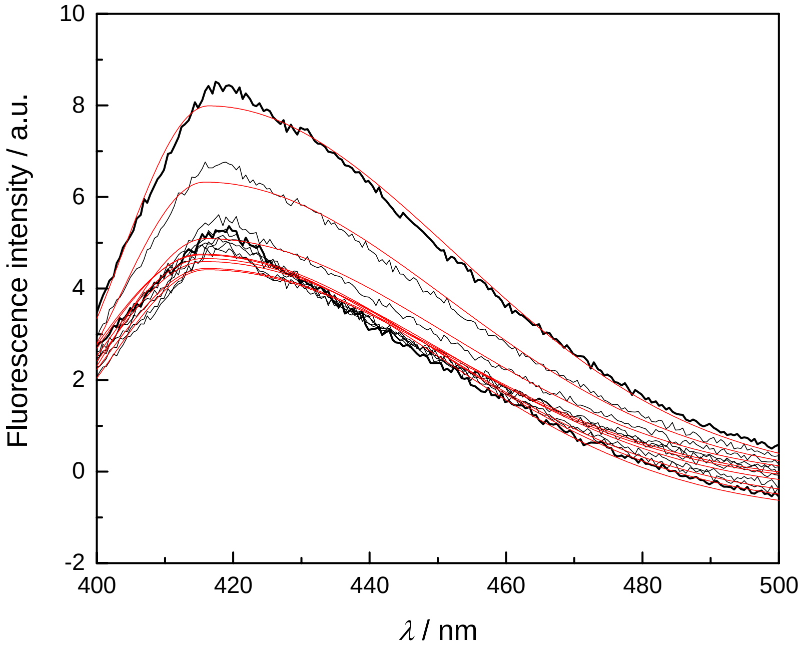

Figure S44. Emission spectra (black line) and fitting to a bigaussian peak function (red line) for oligonucleotide ON1pp, recorded at 10 °C intervals between 10 and 90 °C; [oligonucleotides] = 50 nm; pH = 7.4 (20 mM cacodylate buffer); I = 0.10 M (adjusted with $NaClO_4$); λ_{ex} = 365 nm. The spectra obtained at the extreme temperatures are represented by thicker lines.

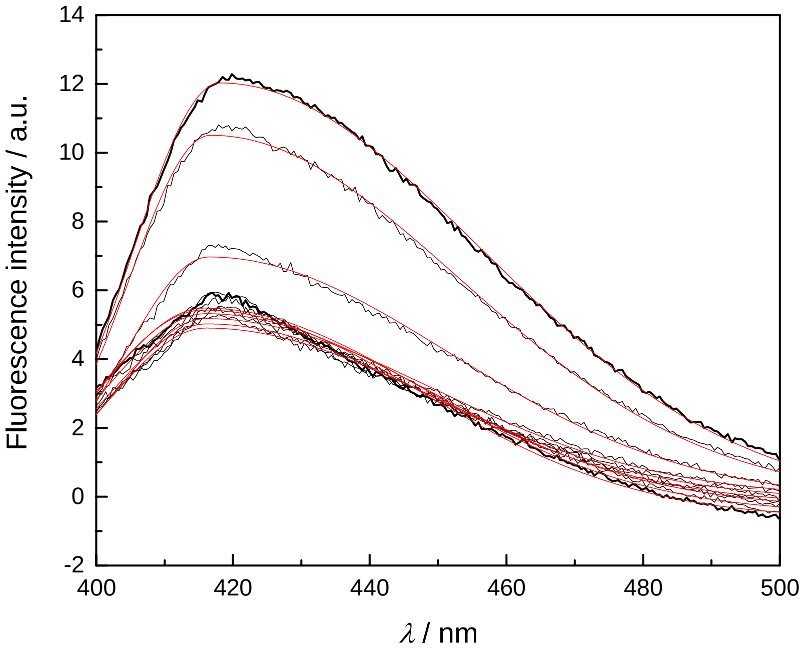

Figure S45. Emission spectra (black line) and fitting to a bigaussian peak function (red line) for duplex ON1pp•ON2a, recorded at 10 °C intervals between 10 and 90 °C; [oligonucleotides] = 50 nm; pH = 7.4 (20 mM cacodylate buffer); I = 0.10 M (adjusted with $NaClO_4$); λ_{ex} = 365 nm. The spectra obtained at the extreme temperatures are represented by thicker lines.

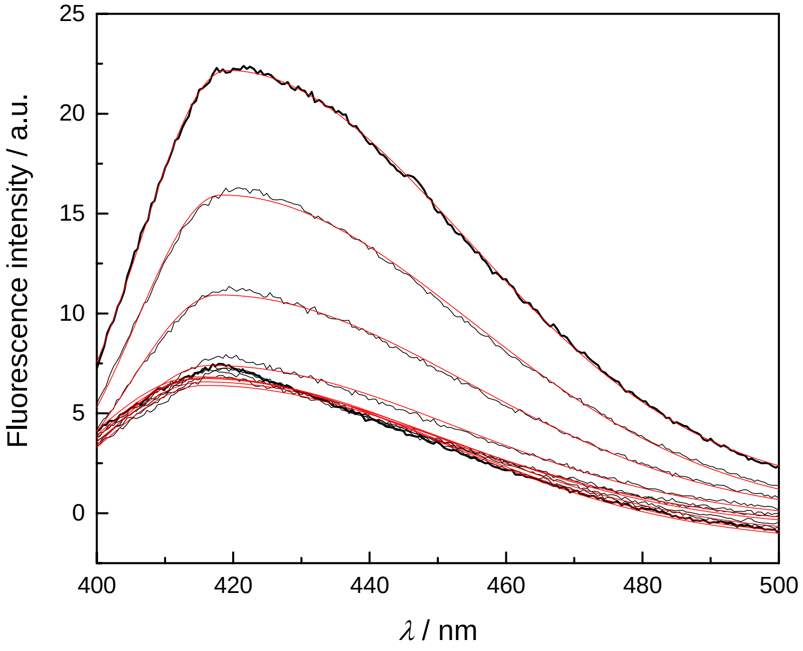

Figure S46. Emission spectra (black line) and fitting to a bigaussian peak function (red line) for duplex ON1pp•ON2c, recorded at 10 °C intervals between 10 and 90 °C; [oligonucleotides] = 50 nm; pH = 7.4 (20 mM cacodylate buffer); I = 0.10 M (adjusted with $NaClO_4$); λ_{ex} = 365 nm. The spectra obtained at the extreme temperatures are represented by thicker lines.

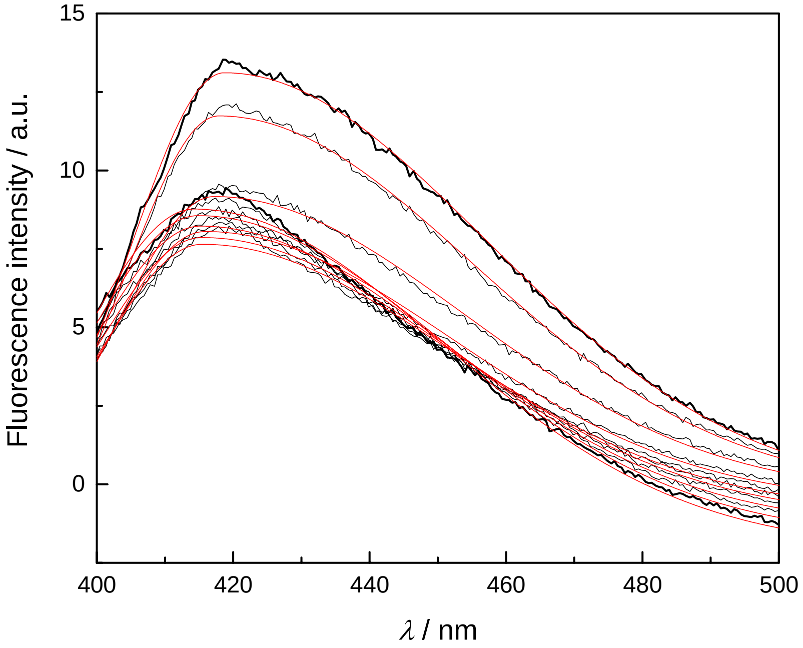

Figure S47. Emission spectra (black line) and fitting to a bigaussian peak function (red line) for duplex ON1pp•ON2g, recorded at 10 °C intervals between 10 and 90 °C; [oligonucleotides] = 50 nm; pH = 7.4 (20 mM cacodylate buffer); I = 0.10 M (adjusted with $NaClO_4$); λ_{ex} = 365 nm. The spectra obtained at the extreme temperatures are represented by thicker lines.

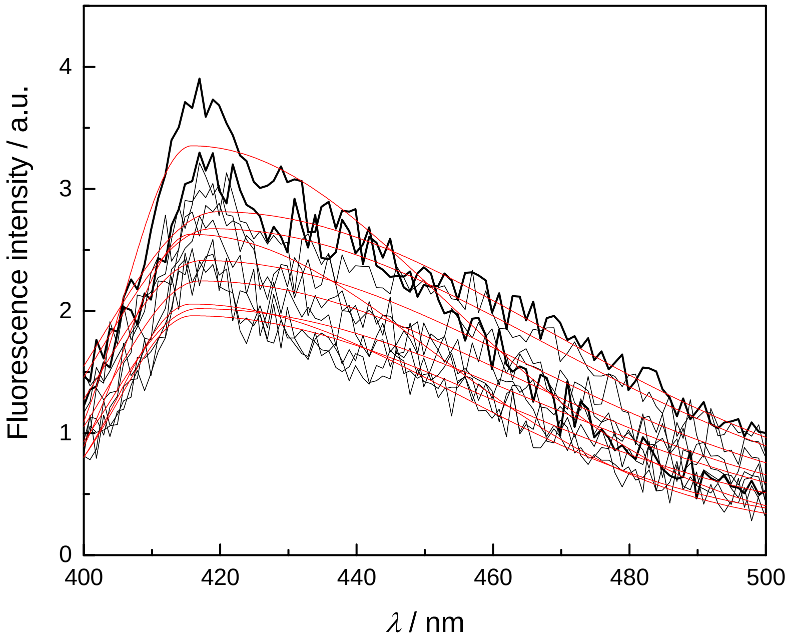

Figure S48. Emission spectra (black line) and fitting to a bigaussian peak function (red line) for duplex ON1pp•ON2t, recorded at 10 °C intervals between 10 and 90 °C; [oligonucleotides] = 50 nm; pH = 7.4 (20 mM cacodylate buffer); *I* = 0.10 M (adjusted with $NaClO_4$); λ_{ex} = 365 nm. The spectra obtained at the extreme temperatures are represented by thicker lines.

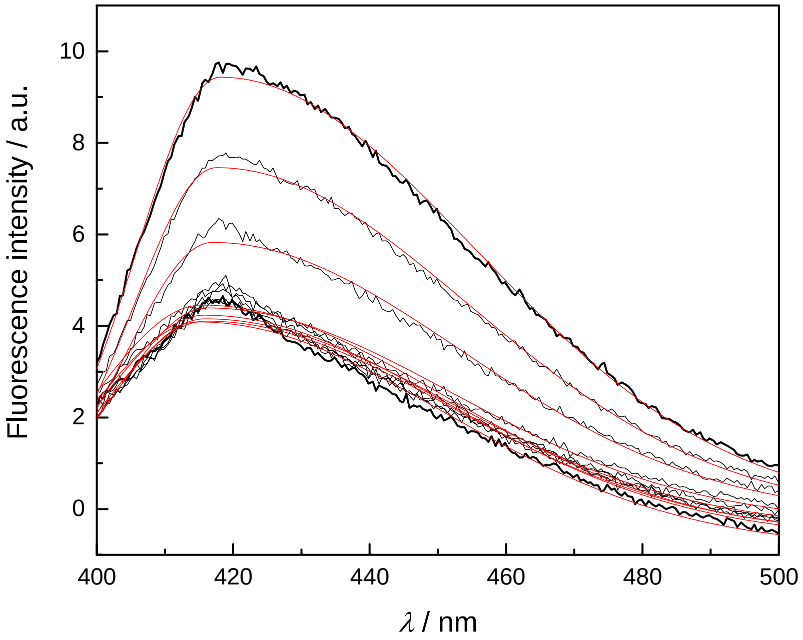

Figure S49. Emission spectra (black line) and fitting to a bigaussian peak function (red line) for duplex ON1pp•ON2s, recorded at 10 °C intervals between 10 and 90 °C; [oligonucleotides] = 50 nm; pH = 7.4 (20 mM cacodylate buffer); *I* = 0.10 M (adjusted with $NaClO_4$); λ_{ex} = 365 nm. The spectra obtained at the extreme temperatures are represented by thicker lines.

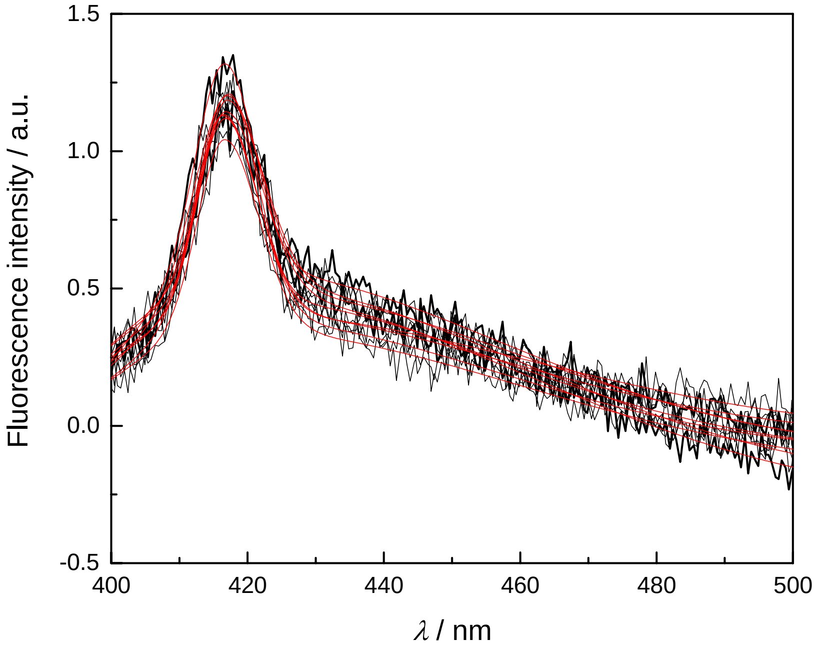

Figure S50. Emission spectra (black line) and fitting to a bigaussian peak function (red line) for oligonucleotide ON1pp-Pd, recorded at 10 °C intervals between 10 and 90 °C; [oligonucleotides] = 50 nm; pH = 7.4 (20 mM cacodylate buffer); *I* = 0.10 M (adjusted with $NaClO_4$); λ_{ex} = 365 nm. The spectra obtained at the extreme temperatures are represented by thicker lines.

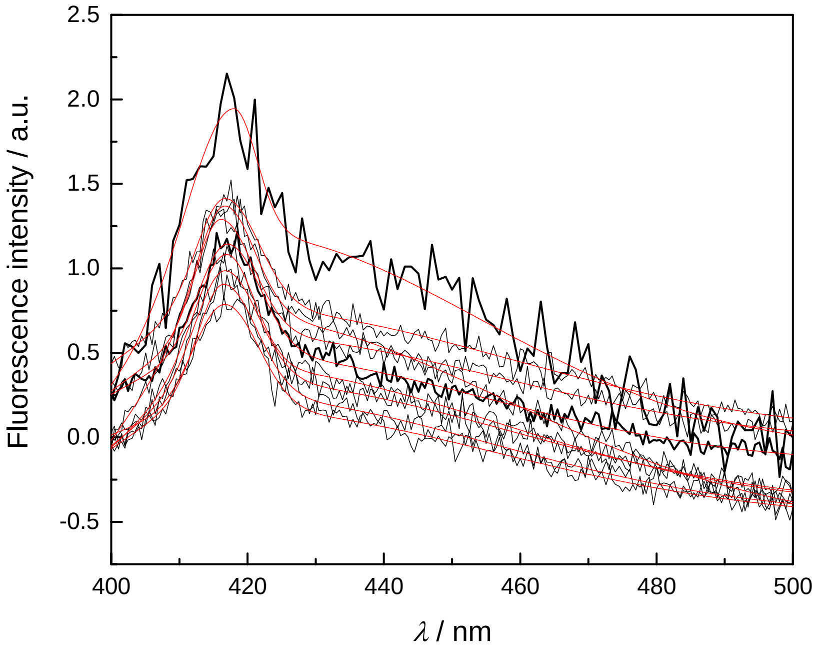

Figure S51. Emission spectra (black line) and fitting to a bigaussian peak function (red line) for duplex ON1pp-Pd•ON2a, recorded at 10 °C intervals between 10 and 90 °C; [oligonucleotides] = 50 nm; pH = 7.4 (20 mM cacodylate buffer); *I* = 0.10 M (adjusted with $NaClO_4$); λ_{ex} = 365 nm. The spectra obtained at the extreme temperatures are represented by thicker lines.

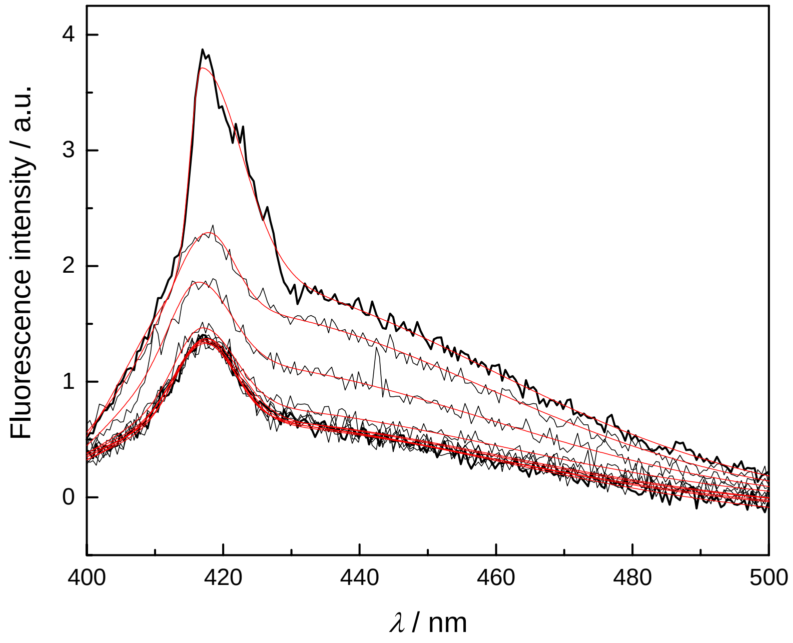

Figure S52. Emission spectra (black line) and fitting to a bigaussian peak function (red line) for duplex ON1pp-Pd•ON2c, recorded at 10 °C intervals between 10 and 90 °C; [oligonucleotides] = 50 nm; pH = 7.4 (20 mM cacodylate buffer); I = 0.10 M (adjusted with $NaClO_4$); λ_{ex} = 365 nm. The spectra obtained at the extreme temperatures are represented by thicker lines.

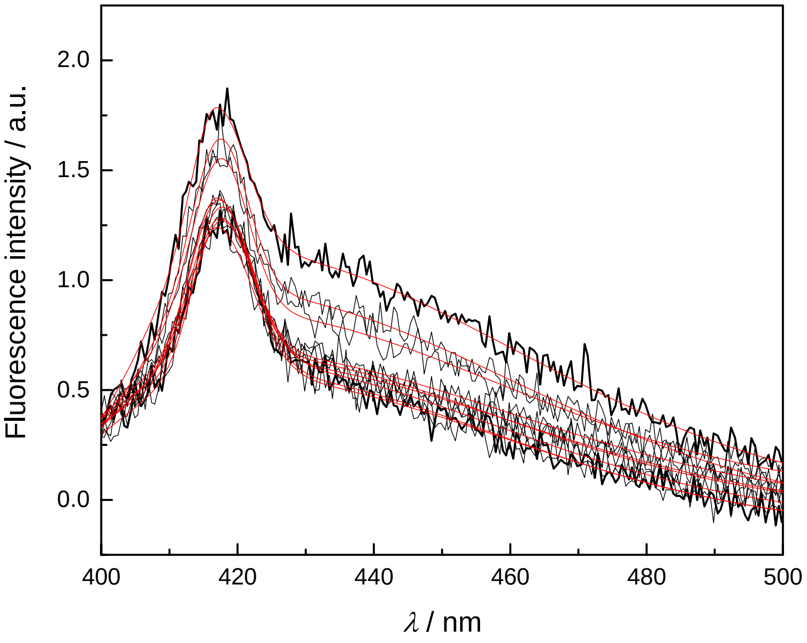

Figure S53. Emission spectra (black line) and fitting to a bigaussian peak function (red line) for duplex ON1pp-Pd•ON2g, recorded at 10 °C intervals between 10 and 90 °C; [oligonucleotides] = 50 nm; pH = 7.4 (20 mM cacodylate buffer); I = 0.10 M (adjusted with $NaClO_4$); λ_{ex} = 365 nm. The spectra obtained at the extreme temperatures are represented by thicker lines.

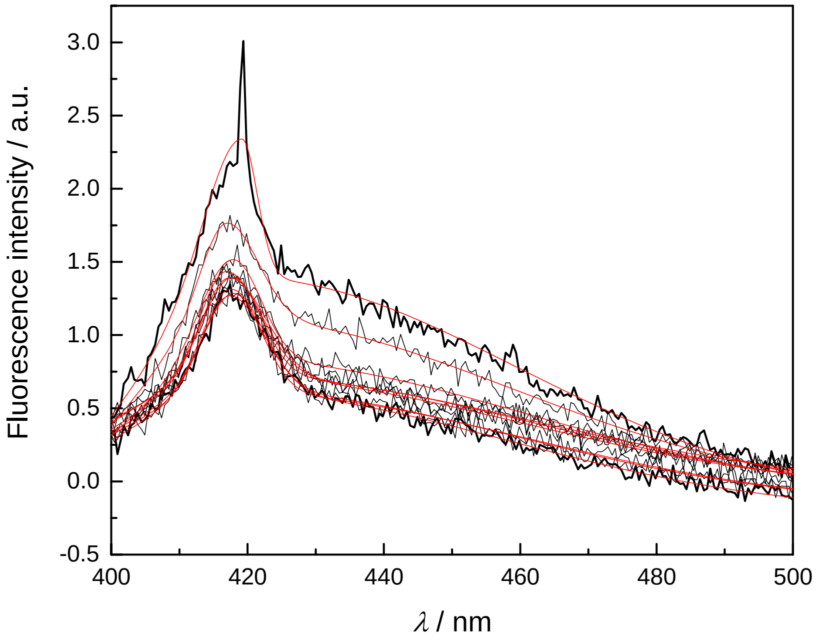

Figure S54. Emission spectra (black line) and fitting to a bigaussian peak function (red line) for duplex ON1pp-Pd•ON2t, recorded at 10 °C intervals between 10 and 90 °C; [oligonucleotides] = 50 nm; pH = 7.4 (20 mM cacodylate buffer); I = 0.10 M (adjusted with $NaClO_4$); λ_{ex} = 365 nm. The spectra obtained at the extreme temperatures are represented by thicker lines.

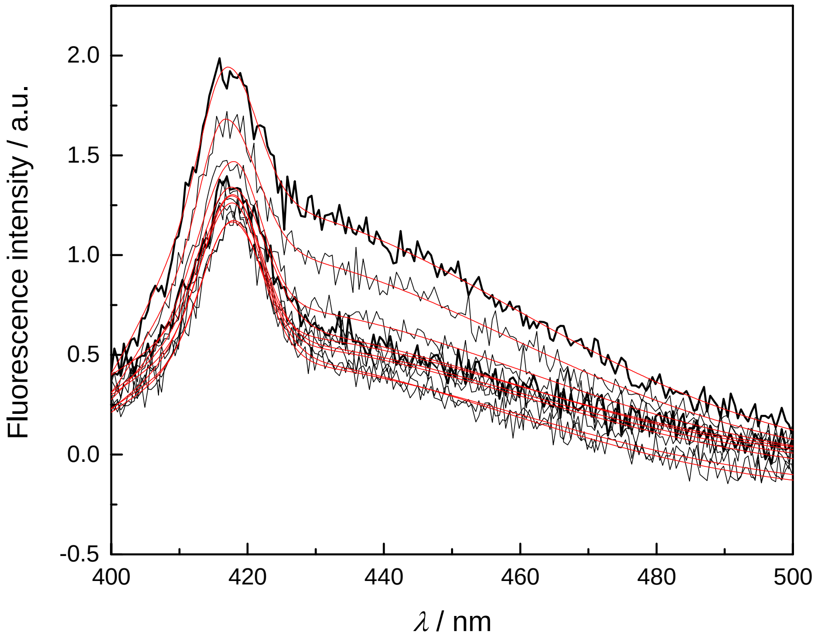

Figure S55. Emission spectra (black line) and fitting to a bigaussian peak function (red line) for duplex ON1pp-Pd•ON2s, recorded at 10 °C intervals between 10 and 90 °C; [oligonucleotides] = 50 nm; pH = 7.4 (20 mM cacodylate buffer); I = 0.10 M (adjusted with $NaClO_4$); λ_{ex} = 365 nm. The spectra obtained at the extreme temperatures are represented by thicker lines.

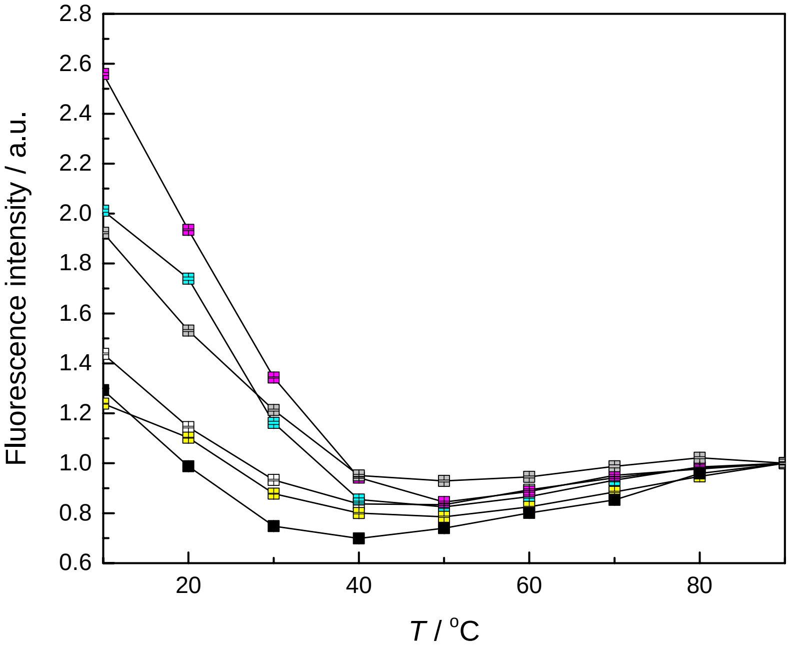

Figure S56. Fluorescence melting profiles for single-stranded ON1pp (white) and duplexes ON1pp•ON2a (cyan), ON1pp•ON2c (magenta), ON1pp•ON2g (yellow), ON1pp•ON2t (black) and ON1pp•ON2s (grey); [oligonucleotides] = 50 nm; pH = 7.4 (20 mM cacodylate buffer); I = 0.10 M (adjusted with $NaClO_4$); λ_{ex} = 365 nm;); λ_{em} = 418 nm.

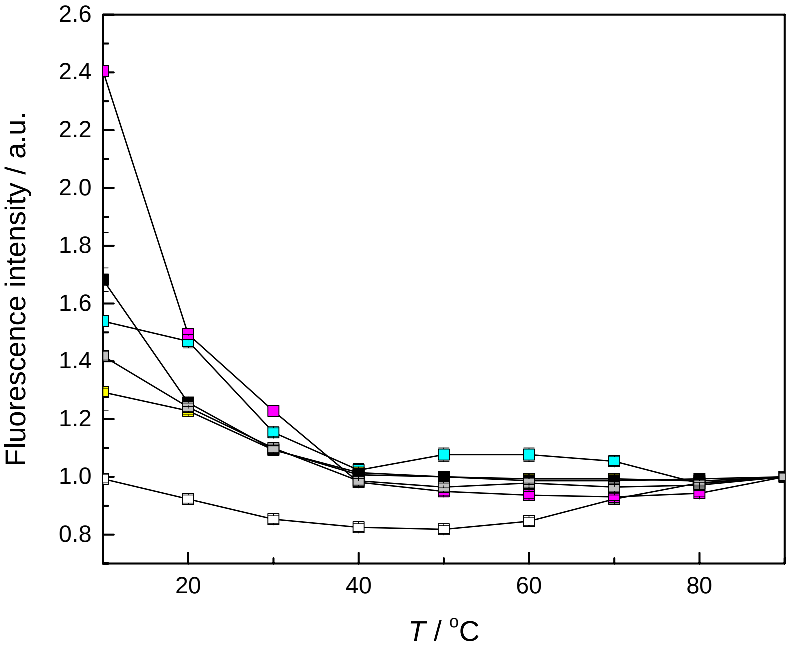

Figure S57. Fluorescence melting profiles for single-stranded ON1pp-Pd (white) and duplexes ON1pp-Pd•ON2a (cyan), ON1pp-Pd•ON2c (magenta), ON1pp-Pd•ON2g (yellow), ON1pp-Pd•ON2t (black) and ON1pp-Pd•ON2s (grey); [oligonucleotides] = 50 nm; pH = 7.4 (20 mM cacodylate buffer); I = 0.10 M (adjusted with $NaClO_4$); λ_{ex} = 365 nm;); λ_{em} = 418 nm.

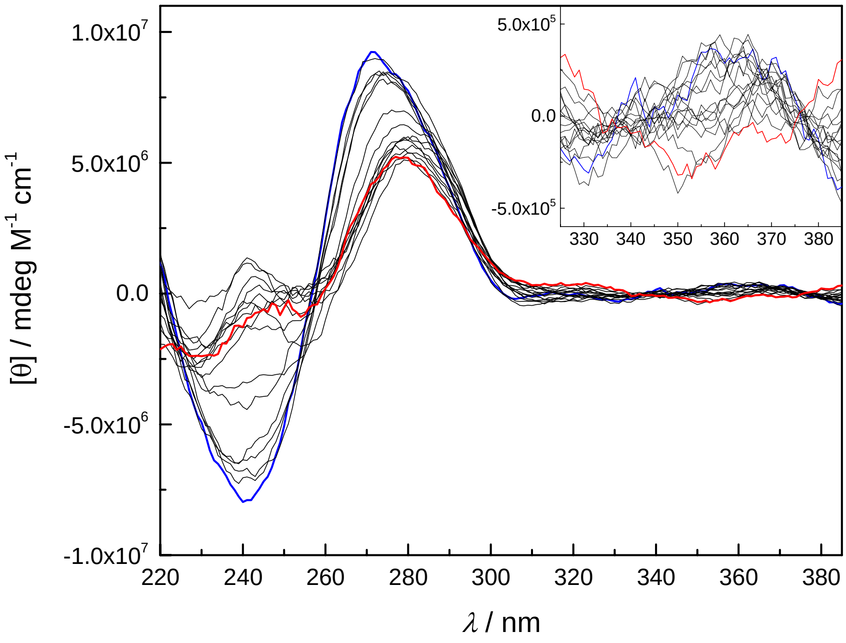

Figure S58. CD spectra of duplex ON1pp•ON2a, recorded at 5 °C intervals between 10 and 90 °C; [oligonucleotides] = 1.0 µM; pH = 7.4 (20 mM cacodylate buffer); *I* = 0.10 M (adjusted with $NaClO_4$). The spectra obtained at 10 and 90 °C are represented by thicker blue and red lines, respectively.

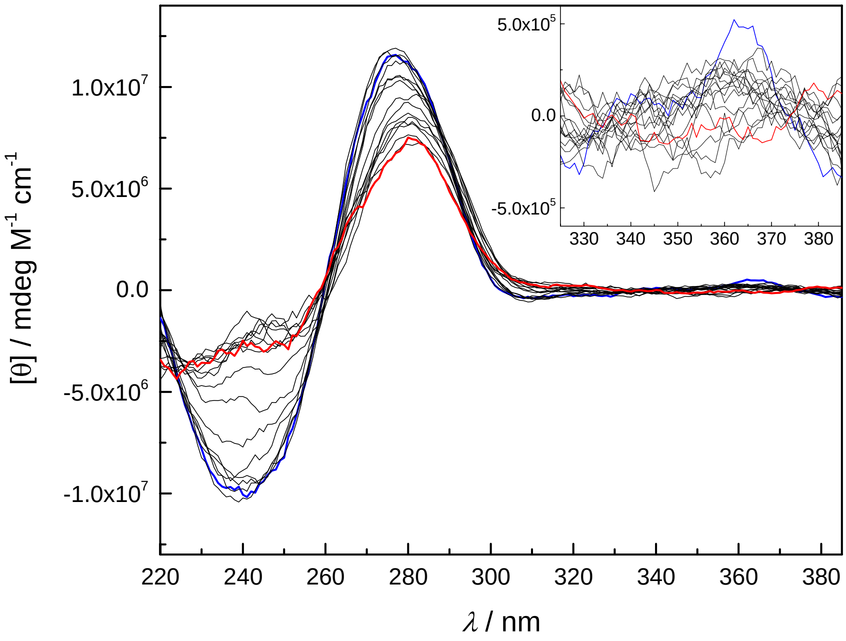

Figure S59. CD spectra of duplex ON1pp•ON2c, recorded at 5 °C intervals between 10 and 90 °C; [oligonucleotides] = 1.0 µM; pH = 7.4 (20 mM cacodylate buffer); *I* = 0.10 M (adjusted with $NaClO_4$). The spectra obtained at 10 and 90 °C are represented by thicker blue and red lines, respectively.

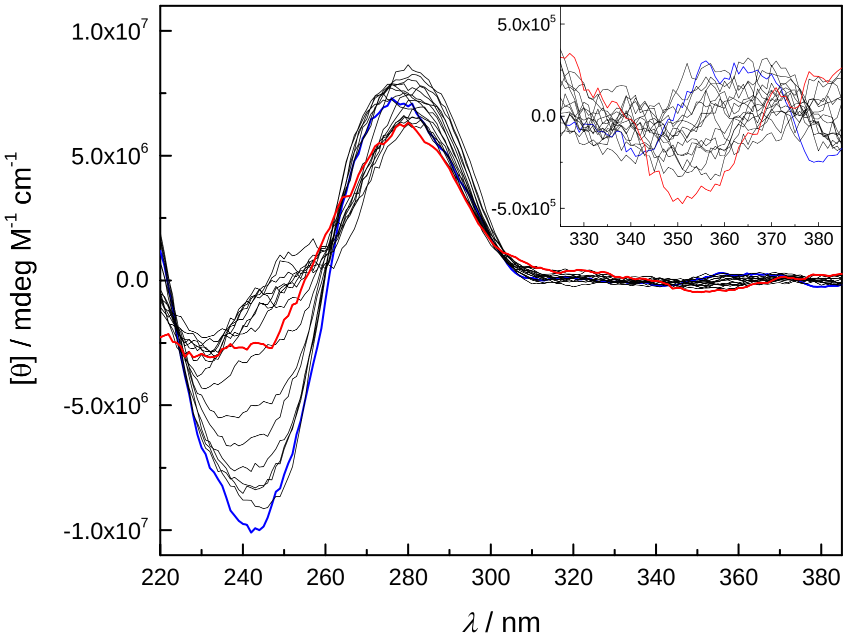

Figure S60. CD spectra of duplex ON1pp•ON2g, recorded at 5 °C intervals between 10 and 90 °C; [oligonucleotides] = 1.0 µM; pH = 7.4 (20 mM cacodylate buffer); *I* = 0.10 M (adjusted with $NaClO_4$). The spectra obtained at 10 and 90 °C are represented by thicker blue and red lines, respectively.

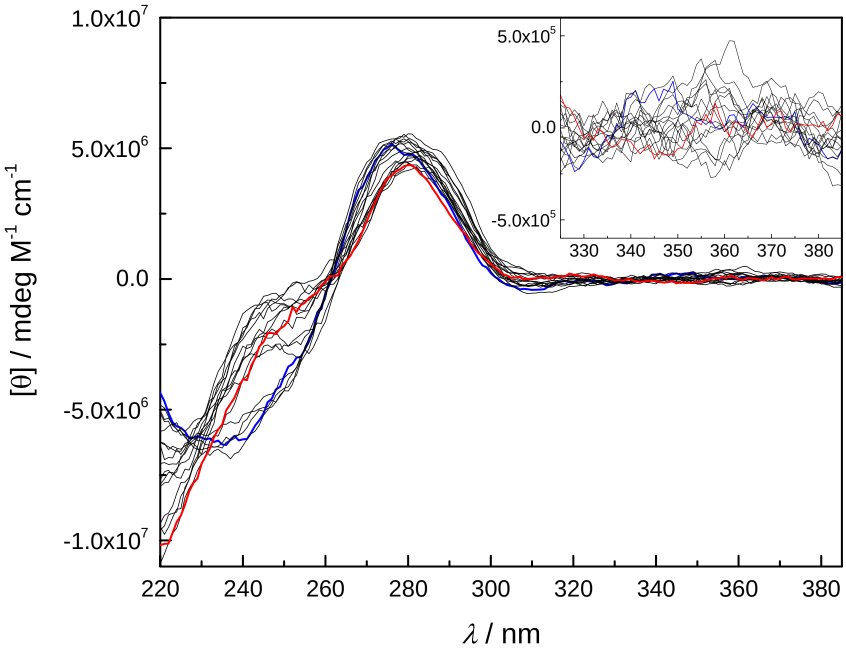

Figure S61. CD spectra of duplex ON1pp•ON2t, recorded at 5 °C intervals between 10 and 90 °C; [oligonucleotides] = 1.0 µM; pH = 7.4 (20 mM cacodylate buffer); *I* = 0.10 M (adjusted with $NaClO_4$). The spectra obtained at 10 and 90 °C are represented by thicker blue and red lines, respectively.

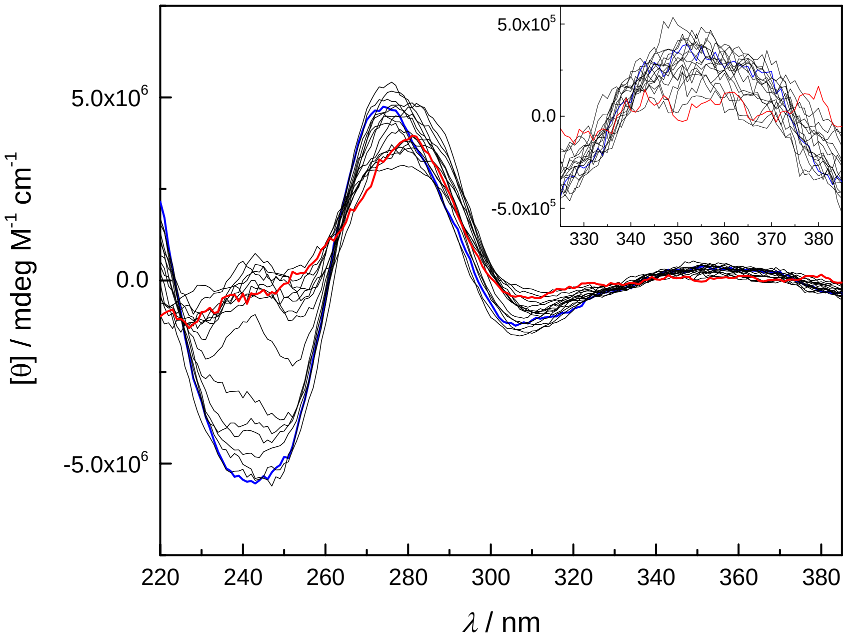

Figure S62. CD spectra of duplex ON1pp•ON2s, recorded at 5 °C intervals between 10 and 90 °C; [oligonucleotides] = 1.0 μM; pH = 7.4 (20 mM cacodylate buffer); *I* = 0.10 M (adjusted with $NaClO_4$). The spectra obtained at 10 and 90 °C are represented by thicker blue and red lines, respectively.

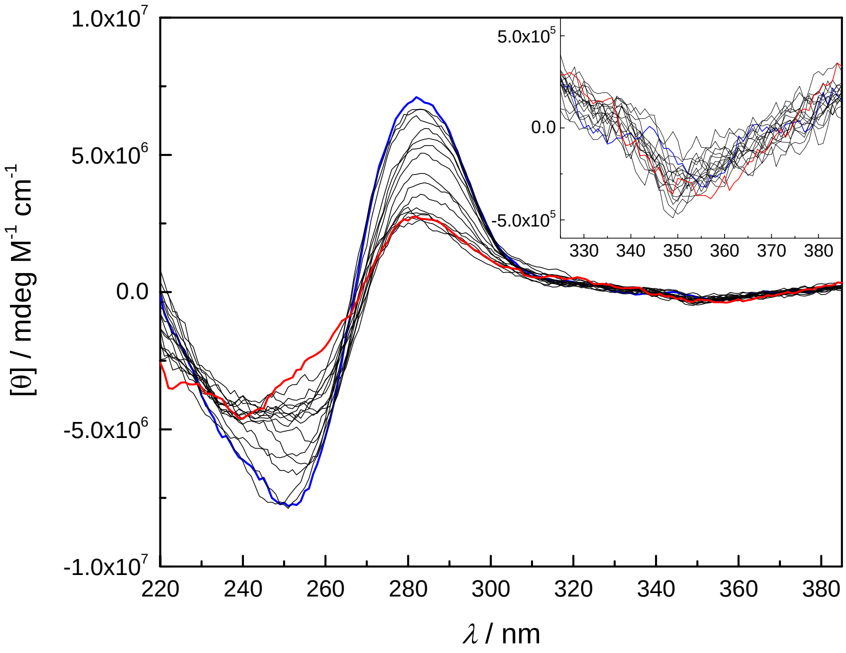

Figure S63. CD spectra of duplex ON1pp-Pd•ON2a, recorded at 5 °C intervals between 10 and 90 °C; [oligonucleotides] = 1.0 μM; pH = 7.4 (20 mM cacodylate buffer); *I* = 0.10 M (adjusted with $NaClO_4$). The spectra obtained at 10 and 90 °C are represented by thicker blue and red lines, respectively.

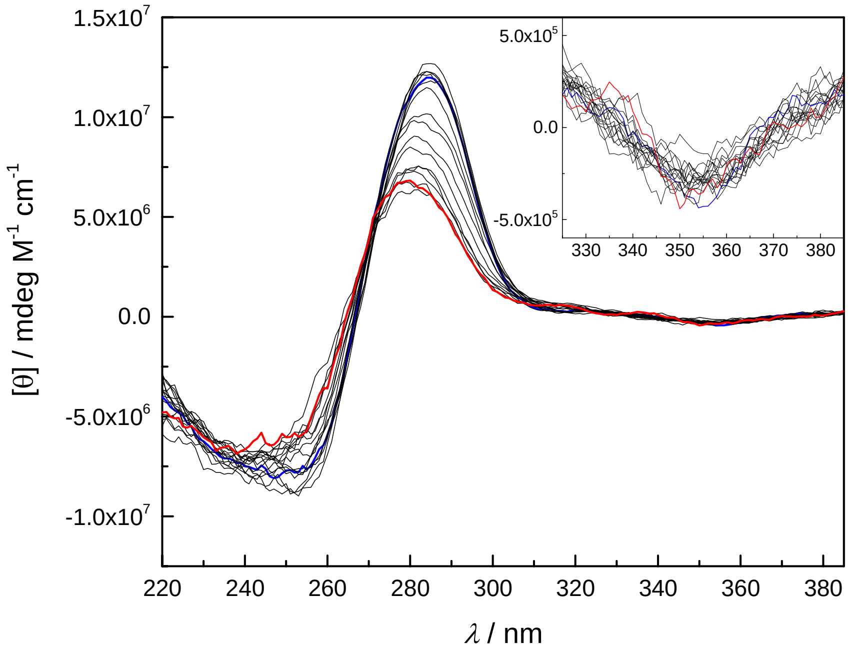

Figure S64. CD spectra of duplex ON1pp-Pd•ON2c, recorded at 5 °C intervals between 10 and 90 °C; [oligonucleotides] = 1.0 μM; pH = 7.4 (20 mM cacodylate buffer); *I* = 0.10 M (adjusted with $NaClO_4$). The spectra obtained at 10 and 90 °C are represented by thicker blue and red lines, respectively.

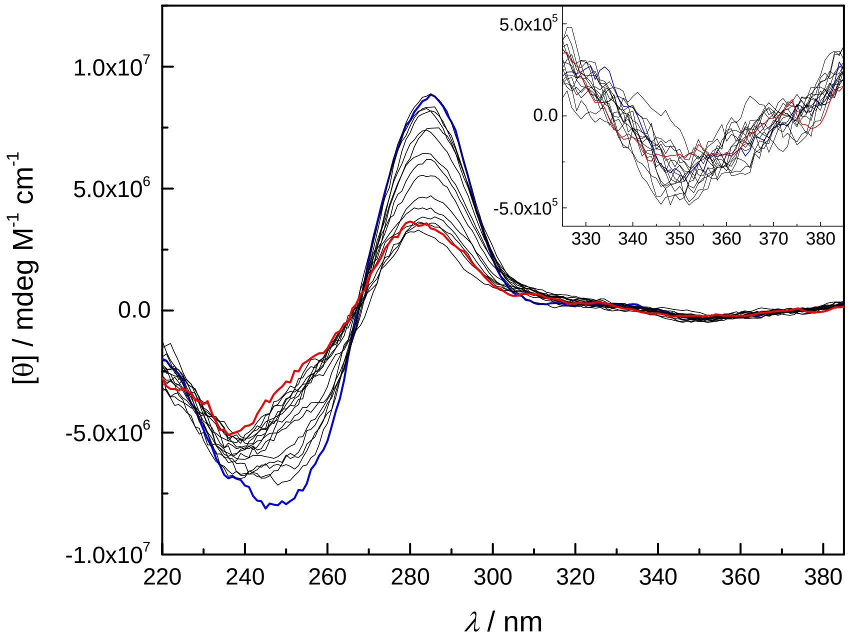

Figure S65. CD spectra of duplex ON1pp-Pd•ON2g, recorded at 5 °C intervals between 10 and 90 °C; [oligonucleotides] = 1.0 μM; pH = 7.4 (20 mM cacodylate buffer); *I* = 0.10 M (adjusted with $NaClO_4$). The spectra obtained at 10 and 90 °C are represented by thicker blue and red lines, respectively.

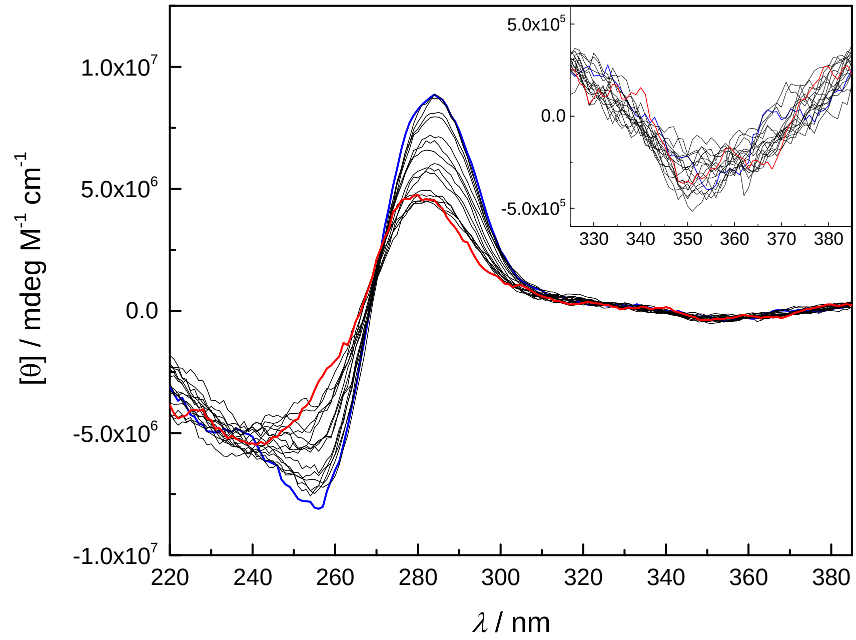

Figure S66. CD spectra of duplex ON1pp-Pd•ON2t, recorded at 5 °C intervals between 10 and 90 °C; [oligonucleotides] = 1.0 µM; pH = 7.4 (20 mM cacodylate buffer); *I* = 0.10 M (adjusted with $NaClO_4$). The spectra obtained at 10 and 90 °C are represented by thicker blue and red lines, respectively.

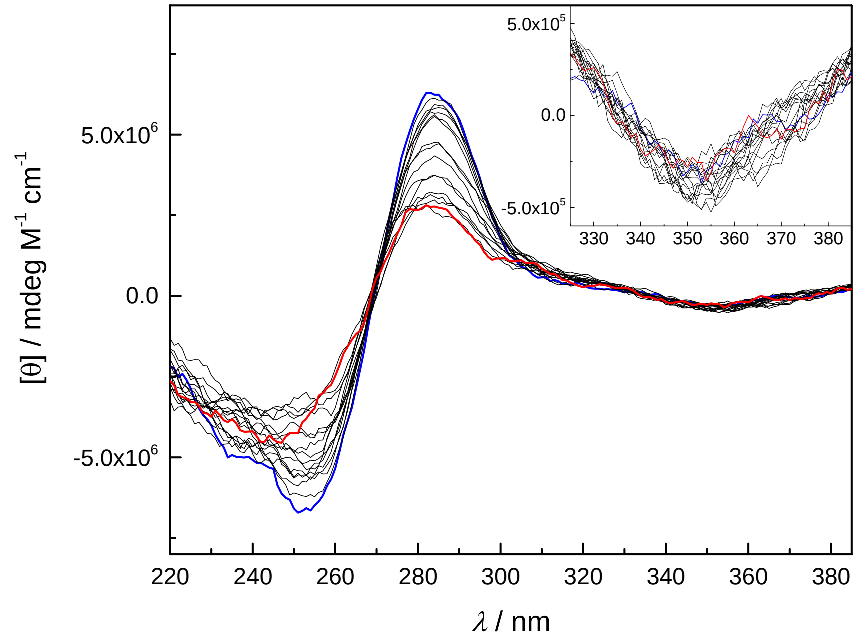

Figure S67. CD spectra of duplex ON1pp-Pd•ON2s, recorded at 5 °C intervals between 10 and 90 °C; [oligonucleotides] = 1.0 µM; pH = 7.4 (20 mM cacodylate buffer); *I* = 0.10 M (adjusted with $NaClO_4$). The spectra obtained at 10 and 90 °C are represented by thicker blue and red lines, respectively.

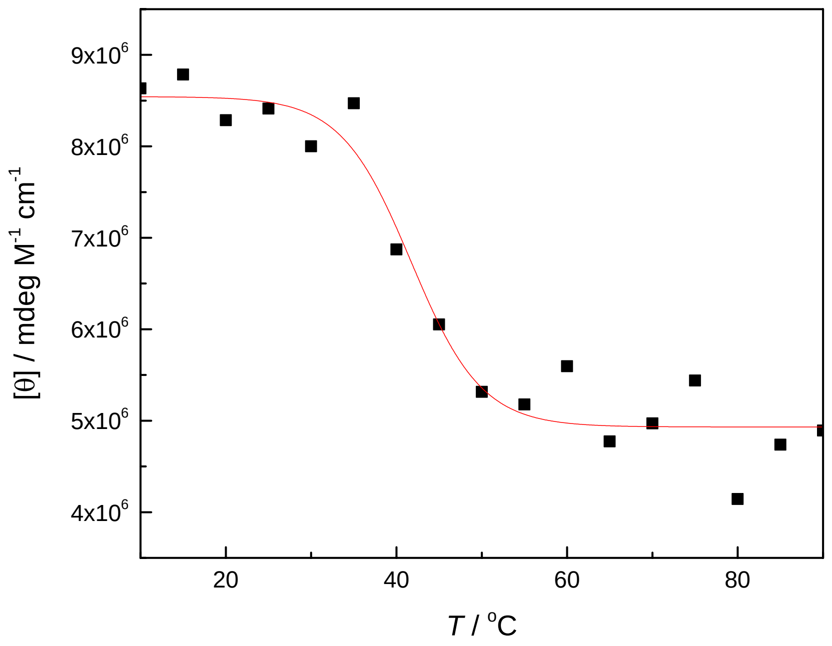

Figure S68. CD melting profile (■) and fitting to Equation 1 (red line) for duplex ON1pp•ON2a; [oligonucleotides] = 1.0 µM; pH = 7.4 (20 mM cacodylate buffer); *I* = 0.10 M (adjusted with $NaClO_4$); λ = 275 nm.

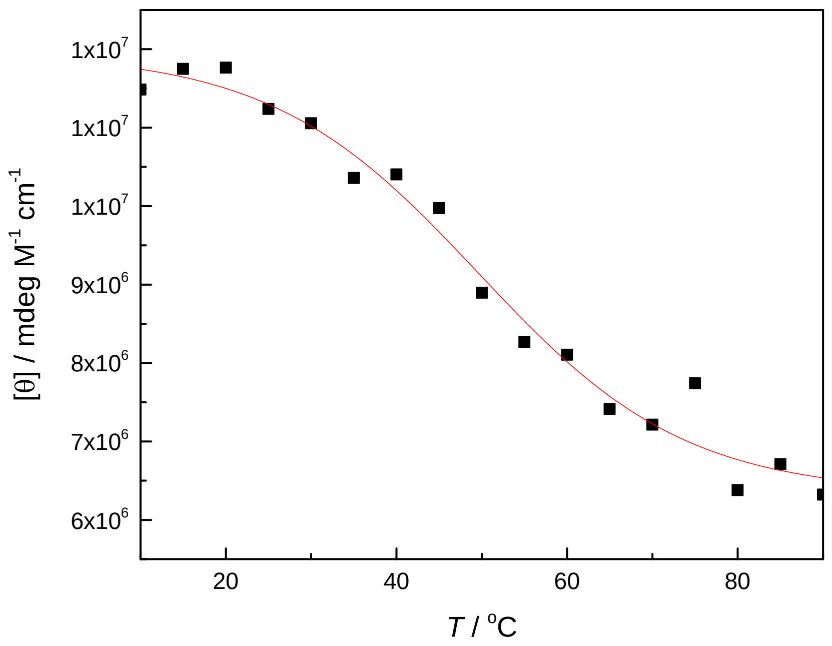

Figure S69. CD melting profile (■) and fitting to Equation 1 (red line) for duplex ON1pp•ON2c; [oligonucleotides] = 1.0 µM; pH = 7.4 (20 mM cacodylate buffer); *I* = 0.10 M (adjusted with $NaClO_4$); λ = 275 nm.

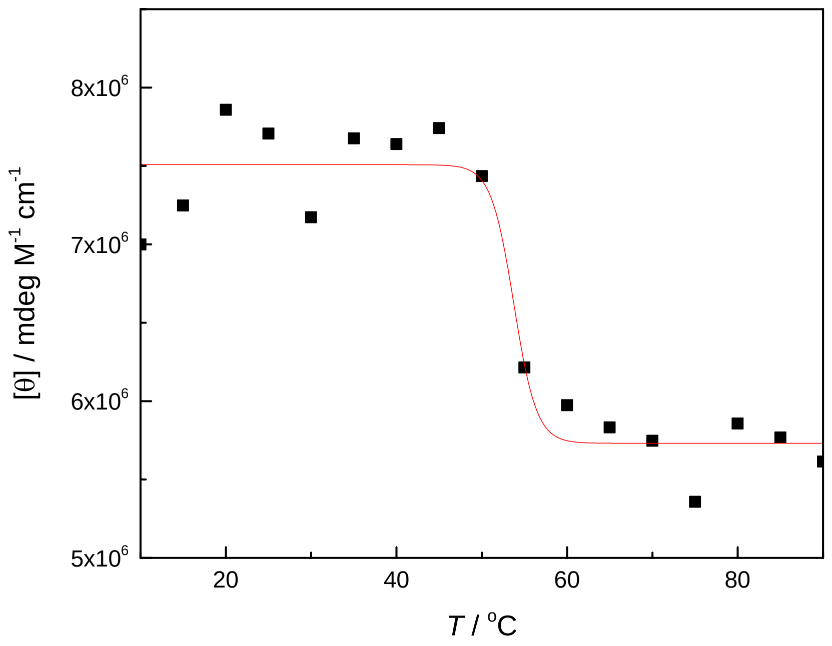

Figure S70. CD melting profile (■) and fitting to Equation 1 (red line) for duplex ON1pp•ON2g; [oligonucleotides] = 1.0 µM; pH = 7.4 (20 mM cacodylate buffer); I = 0.10 M (adjusted with $NaClO_4$); λ = 275 nm.

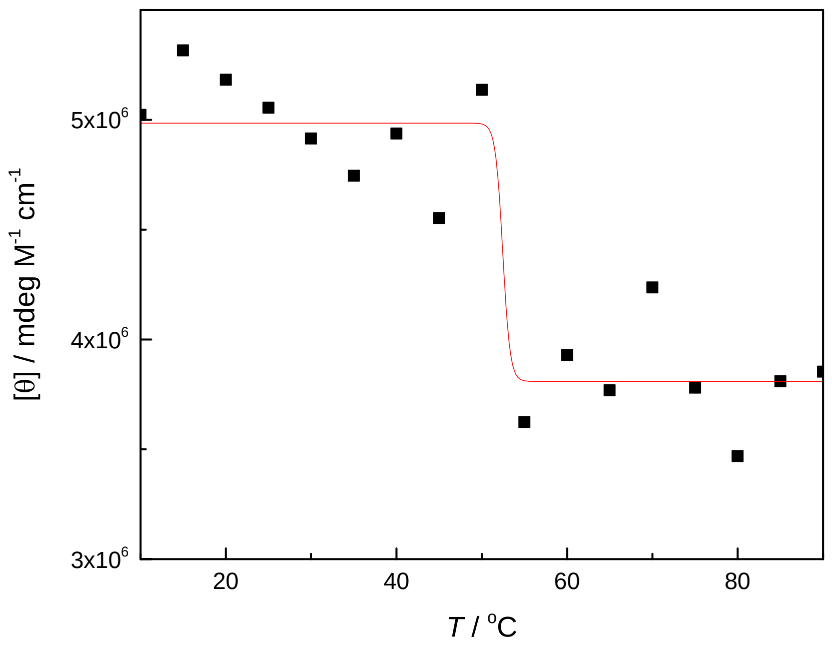

Figure S71. CD melting profile (■) and fitting to Equation 1 (red line) for duplex ON1pp•ON2t; [oligonucleotides] = 1.0 µM; pH = 7.4 (20 mM cacodylate buffer); I = 0.10 M (adjusted with $NaClO_4$); λ = 275 nm.

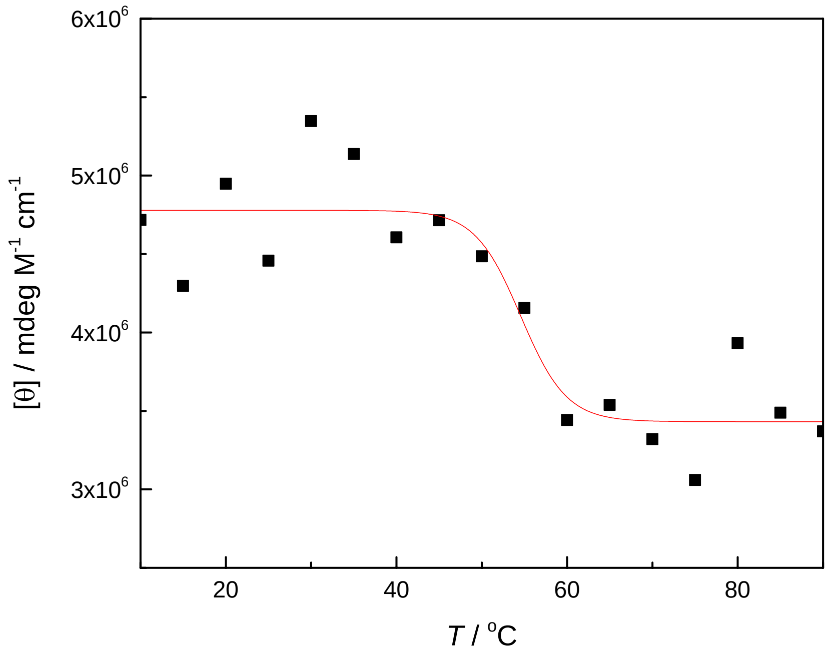

Figure S72. CD melting profile (■) and fitting to Equation 1 (red line) for duplex ON1pp•ON2s; [oligonucleotides] = 1.0 µM; pH = 7.4 (20 mM cacodylate buffer); I = 0.10 M (adjusted with $NaClO_4$); λ = 275 nm.

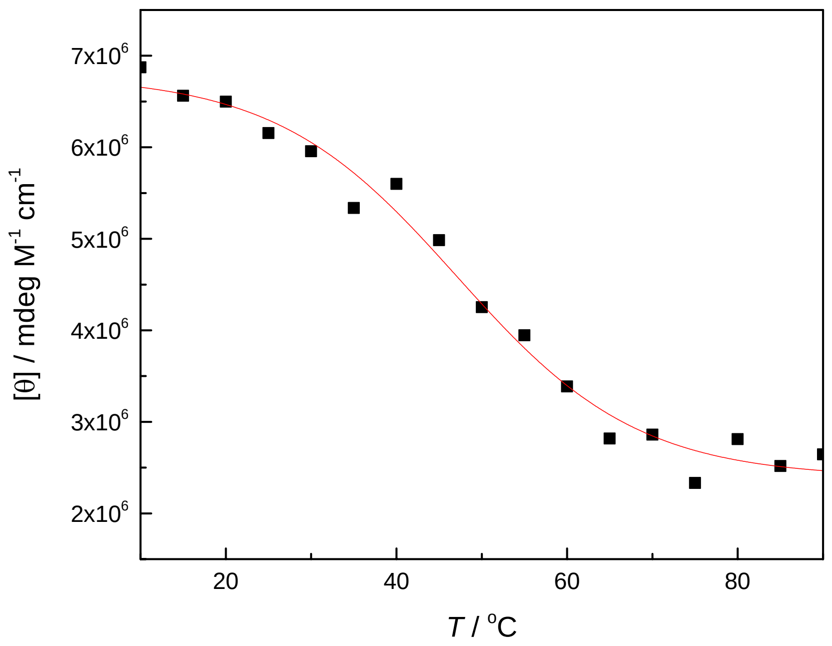

Figure S73. CD melting profile (■) and fitting to Equation 1 (red line) for duplex ON1pp-Pd•ON2a; [oligonucleotides] = 1.0 µM; pH = 7.4 (20 mM cacodylate buffer); I = 0.10 M (adjusted with $NaClO_4$); λ = 285 nm.

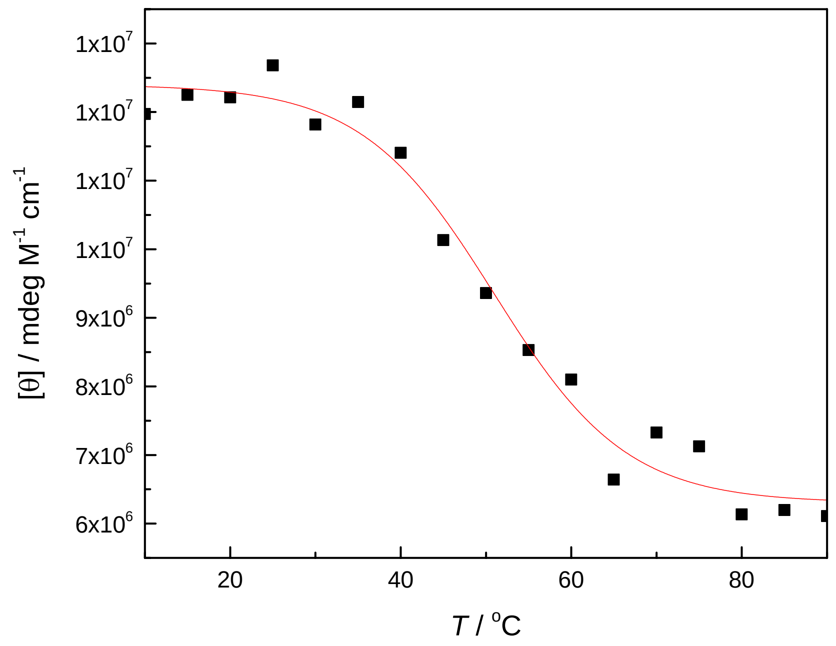

Figure S74. CD melting profile (■) and fitting to Equation 1 (red line) for duplex ON1pp-Pd•ON2c; [oligonucleotides] = 1.0 µM; pH = 7.4 (20 mM cacodylate buffer); I = 0.10 M (adjusted with $NaClO_4$); λ = 285 nm.

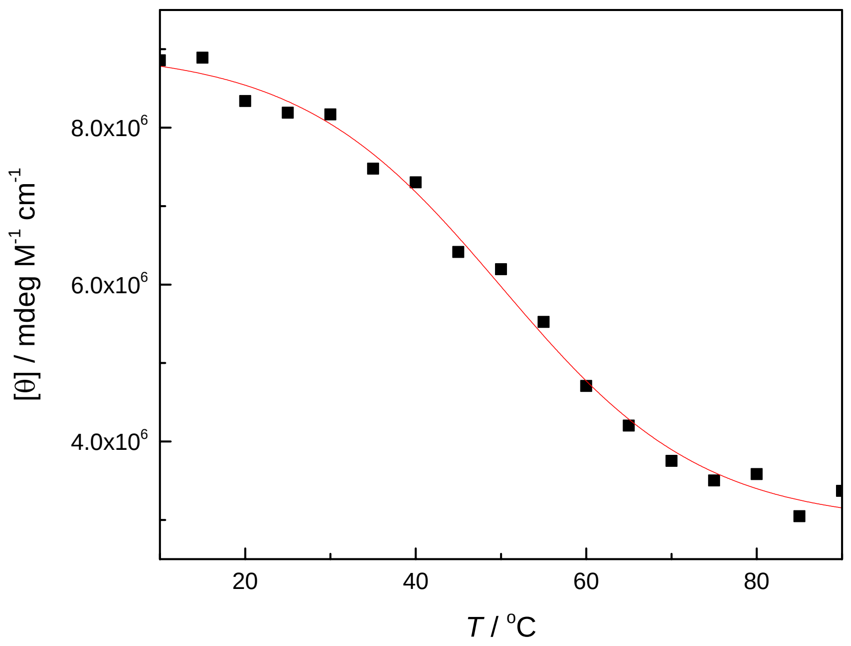

Figure S75. CD melting profile (■) and fitting to Equation 1 (red line) for duplex ON1pp-Pd•ON2g; [oligonucleotides] = 1.0 µM; pH = 7.4 (20 mM cacodylate buffer); I = 0.10 M (adjusted with $NaClO_4$); λ = 285 nm.

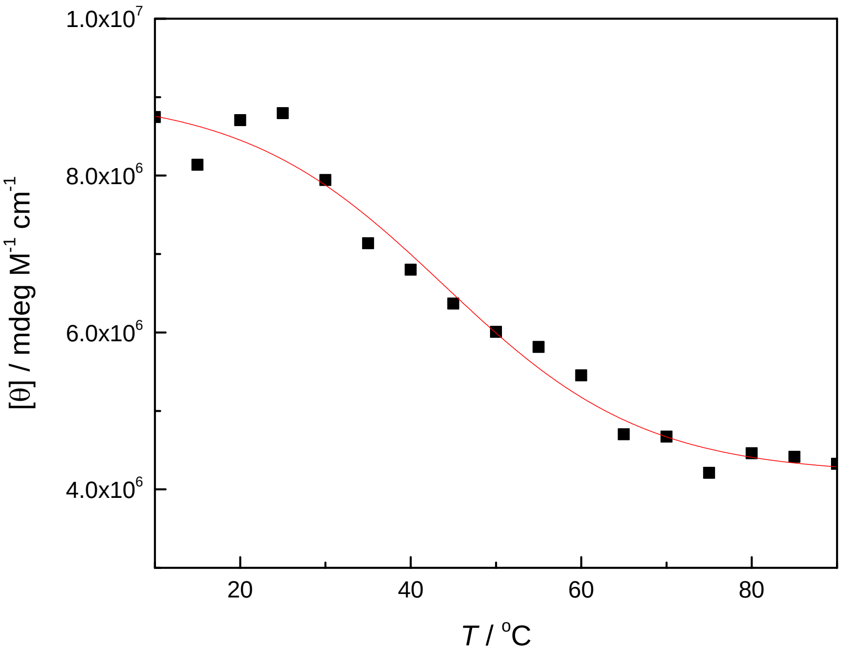

Figure S76. CD melting profile (■) and fitting to Equation 1 (red line) for duplex ON1pp-Pd•ON2t; [oligonucleotides] = 1.0 μM; pH = 7.4 (20 mM cacodylate buffer); *I* = 0.10 M (adjusted with $NaClO_4$); λ = 285 nm.

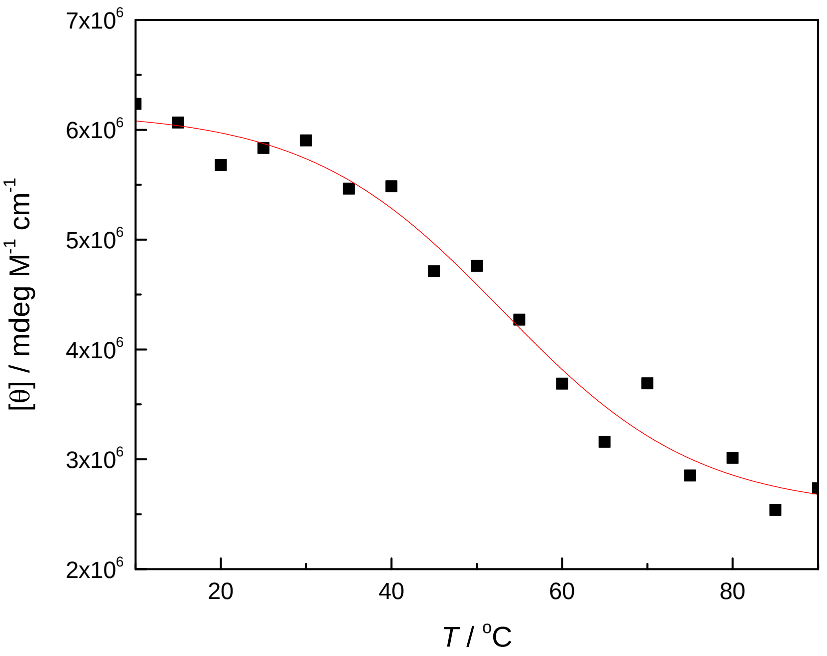

Figure S77. CD melting profile (■) and fitting to Equation 1 (red line) for duplex ON1pp-Pd•ON2s; [oligonucleotides] = 1.0 μM; pH = 7.4 (20 mM cacodylate buffer); *I* = 0.10 M (adjusted with $NaClO_4$); λ = 285 nm.

Table S1. UV and CD melting temperatures and selected photophysical properties of the various metal-free and palladacyclic duplexes; [oligonucleotides] = 1.0 µM (UV and CD measurements) / 50 nM (fluorescence measurements); pH = 7.4 (20 mM cacodylate buffer); I = 0.10 M (adjusted with $NaClO_4$).

Duplex	T_m / °C			ε(365 nm) / L mol^{-1} cm^{-1}	Φ
	UV (260 nm)	UV (425 nm)	CD (275 / 285 nm)		
ON1pp•ON2a	38.5 ± 0.2[b]<br>41.7 ± 0.2[c]	n.a.[a]	42 ± 2	87500	0.83
ON1pp•ON2c	44.0 ± 0.1[b]<br>44.6 ± 0.1[c]	n.a.[a]	50 ± 2	116000	0.92
ON1pp•ON2g	40.3 ± 0.2[b]<br>43.2 ± 0.1[c]	n.a.[a]	54 ± 1	118000	0.71
ON1pp•ON2t	40.6 ± 0.1[b]<br>41.3 ± 0.1[c]	n.a.[a]	50 ± 20	104000	0.19
ON1pp•ON2s	43.4 ± 0.1[b]<br>44.4 ± 0.1[c]	n.a.[a]	55 ± 3	164000	0.32
ON1pp-Pd•ON2a	17 ± 1[b]<br>21.7 ± 0.1[c]	56.1 ± 0.6[b]<br>56.9 ± 0.1[c]	47 ± 2	48100	0.15
ON1pp-Pd•ON2c	9 ± 2[b]<br>3 ± 4[c]	54.0 ± 0.6[b]<br>59.0 ± 0.1[c]	51 ± 2	55300	0.23
ON1pp-Pd•ON2g	23 ± 2[b]<br>23.1 ± 0.2[c]	56.7 ± 0.8[b]<br>60.0 ± 0.1[c]	50 ± 1	45400	0.17
ON1pp-Pd•ON2t	33 ± 2[b]<br>18.0 ± 0.2[c]	56.8 ± 0.9[b]<br>62.4 ± 0.1[c]	44 ± 3	48700	0.17
ON1pp-Pd•ON2s	27 ± 1[b]<br>19.9 ± 0.1[c]	57.0 ± 0.9[b]<br>62.6 ± 0.1[c]	53 ± 3	30200	0.26

[a] No absorbance at this wavelength.
[b] By fitting to Equation 1.
[c] By Gaussian fitting to the first-derivative curve. In case of multiple maxima, the highest one was selected for fitting.
